# Orange/Red Benzo[1,2-*b*:4,5-*b*′]dithiophene 1,1,5,5-Tetraoxide-Based Emitters for Luminescent Solar Concentrators: Effect of Structures on Fluorescence Properties and Device Performances

**DOI:** 10.1021/acsaem.3c00362

**Published:** 2023-04-20

**Authors:** Matteo Bartolini, Cosimo Micheletti, Alberto Picchi, Carmen Coppola, Adalgisa Sinicropi, Mariangela Di Donato, Paolo Foggi, Alessandro Mordini, Gianna Reginato, Andrea Pucci, Lorenzo Zani, Massimo Calamante

**Affiliations:** † Institute of Chemistry of Organometallic Compounds (CNR-ICCOM), Via Madonna del Piano 10, 50019 Sesto Fiorentino, Italy; ‡ Department of Chemistry and Industrial Chemistry, University of Pisa, Via G. Moruzzi 13, 56124 Pisa, Italy; § Department of Biotechnology, Chemistry and Pharmacy, R^2^ES Lab, University of Siena, Via A. Moro 2, 53100 Siena, Italy; ∥ CSGI, Consorzio per lo Sviluppo dei Sistemi a Grande Interfase, Via della Lastruccia 3, 50019 Sesto Fiorentino, Italy; ⊥ LENS, European Laboratory for Non-Linear Spectroscopy, Via N. Carrara 1, 50019 Sesto Fiorentino, Italy; # Department of Chemistry, Biology and Biotechnology, University of Perugia, Via Elce di Sotto 8, 06123 Perugia, Italy; ¶ Department of Chemistry “U. Schiff”, University of Florence, Via della Lastruccia 13, 50019 Sesto Fiorentino, Italy; ∇ National Institute of Optics (CNR-INO), Via N. Carrara 1, 50019 Sesto Fiorentino, Italy

**Keywords:** luminescent solar concentrators, organic emitters, benzo[1,2-*b*:4,5-*b*′]dithiophene 1,1,5,5-tetraoxide, DFT calculations, transient absorption spectroscopy, photovoltaics

## Abstract

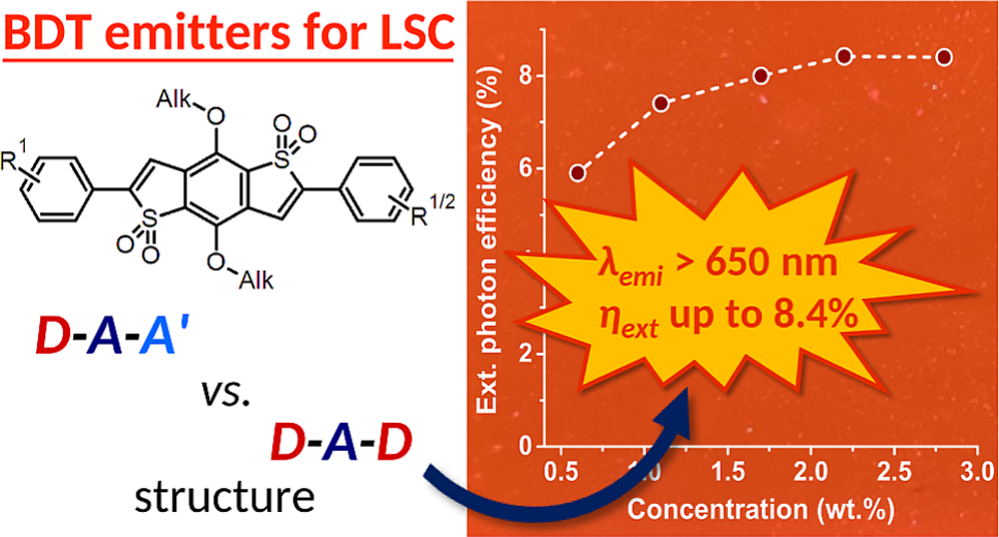

Luminescent solar concentrators (LSCs) are a class of optical devices able to harvest, downshift, and concentrate sunlight, thanks to the presence of emitting materials embedded in a polymer matrix. Use of LSCs in combination with silicon-based photovoltaic (PV) devices has been proposed as a viable strategy to enhance their ability to harvest diffuse light and facilitate their integration in the built environment. LSC performances can be improved by employing organic fluorophores with strong light absorption in the center of the solar spectrum and intense, red-shifted emission. In this work, we present the design, synthesis, characterization, and application in LSCs of a series of orange/red organic emitters featuring a benzo[1,2-*b*:4,5-*b*′]dithiophene 1,1,5,5-tetraoxide central core as an acceptor (A) unit. The latter was connected to different donor (D) and acceptor (A′) moieties by means of Pd-catalyzed direct arylation reactions, yielding compounds with either symmetric (D–A–D) or non-symmetric (D–A–A′) structures. We found that upon light absorption, the compounds attained excited states with a strong intramolecular charge-transfer character, whose evolution was greatly influenced by the nature of the substituents. In general, symmetric structures showed better photophysical properties for the application in LSCs than their non-symmetric counterparts, and using a donor group of moderate strength such as triphenylamine was found preferable. The best LSC built with these compounds presented photonic (external quantum efficiency of 8.4 ± 0.1%) and PV (device efficiency of 0.94 ± 0.06%) performances close to the state-of-the-art, coupled with a sufficient stability in accelerated aging tests.

## 1 Introduction

Luminescent solar concentrators (LSCs) are a class of optical devices invented in the late 1970s,^1,2^ able to harvest, downshift, and concentrate sunlight on a small-surface area.^3^ Being capable of decoupling the emission direction from the incident light direction, while simultaneously achieving high concentration gains, they have recently been employed for several different purposes, including lighting, driving of chemical reactions, and optical communication.^4^ Besides that, they have also been extensively examined for the application in the field of photovoltaics (PVs), thanks to the possibility of coupling them with crystalline silicon solar cells, as well as other PV technologies.^5,6^ Indeed, based on their ability to harvest diffuse or indirect light,^7^ accompanied by their colorful appearance and light weight, LSCs have been proposed as a potential solution to overcome the traditional limitations of Si-based PV technologies, such as their bulky and heavy structure or the need for direct illumination, and foster their diffusion in the built environment.^8^

An LSC is usually made of a sheet of a common plastic material [e.g., poly(methyl methacrylate) PMMA] containing a fluorescent dopant, able to absorb sunlight and emit it at longer wavelengths. Typically used emitters comprise quantum dots, perovskites, rare-earth complexes, and organic compounds.^3^ Due to the different refractive indexes of air and the plastic material, the emitted radiation is mainly concentrated, via total internal reflection, at the edges of the panel, where Si PV cells can be placed. Clearly, LSC performances are critically related to the nature and properties of their constituting materials, among which the fluorescent emitter plays a key role.

The most important properties an emitter should have for a fruitful application in LSCs are a strong light harvesting ability, a high fluorescence quantum yield (FQY, Φ_f_), and a large Stokes shift to minimize re-absorption of emitted light, coupled with a good compatibility with the chosen polymer matrix.^3^ In recent years, the use of organic fluorescent compounds as emitters for LSCs has been intensely investigated since they allow the tuning of the abovementioned photophysical properties by careful structural modifications, while keeping relatively simple synthetic procedures and low cost.^9^ Most organic emitters present a molecular structure characterized by alternating electron-donating (D) and -accepting (A) moieties since such arrangement usually favors strong intramolecular charge transfer (ICT) processes upon photoexcitation, helping achieve the desired optical properties.^10,11^

Notably, since LSCs are designed to work under natural sunlight, an optimal organic emitter should absorb light in the 500–600 nm range, where solar radiation presents the maximum irradiance,^12^ and display a Stokes shift large enough to induce fluorescence emission in the red/NIR region of the spectrum. The latter would be beneficial to improve the match with the absorption of Si-based solar cells, limiting energy losses due to thermalization and improving the overall power conversion efficiency (PCE) of the system. Nevertheless, achieving high FQY with this kind of emitters has proven challenging, due to the so-called “energy gap law”, stating that nonradiative recombination becomes more and more likely as the energy gap between the first excited state and the ground state becomes smaller.^13^ Therefore, careful structural design is mandatory to achieve the best possible compromise between all these different parameters, while at the same time ensuring sufficient solubility and good processability in the conditions of use.

**Figure 1:**
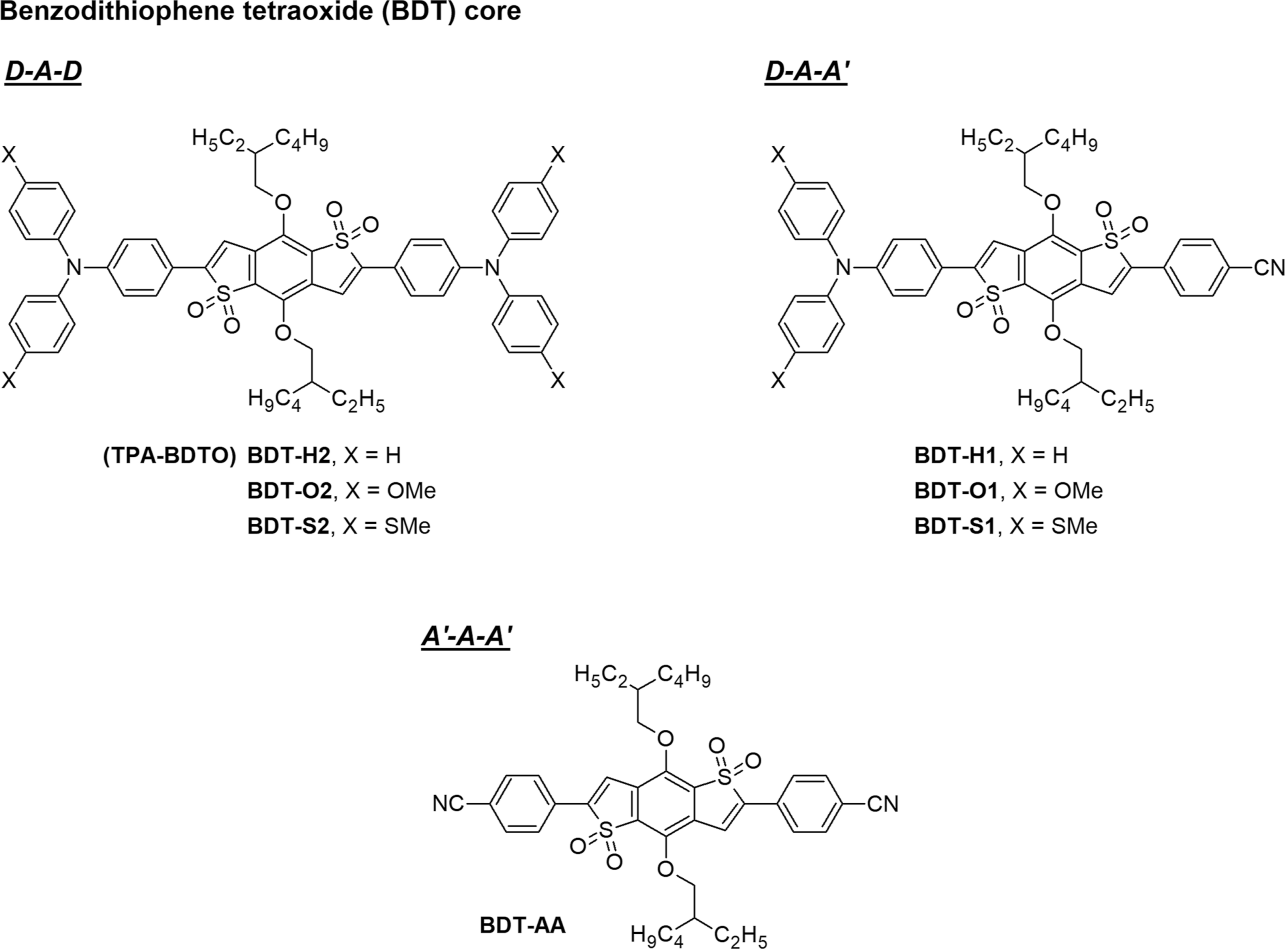
Structures of organic emitter **BDT-H2** (**TPA-BDTO**) and the other compounds designed in this work.

In continuation of our recent activity dedicated to the investigation of new organic emitters for LSCs,^14−17^ we therefore sought to identify suitable heterocyclic structures that could potentially combine all the required optical and physico-chemical properties. Upon careful inspection of the literature, we identified a series of fluorophores containing the strongly electron-withdrawing benzo[1,2-*b*:4,5-*b*′]dithiophene 1,1,5,5-tetraoxide core (BDT-tetraoxide) flanked by different electron-donating groups, originally reported by Tang and co-workers for applications in bio-imaging technologies.^18^ In particular, among the compounds described in the paper, we focused on the **TPA-BDTO** emitter (Figure 1, here also denoted as **BDT-H2**, see below) because besides showing aggregation-induced emission properties,^19^ it displayed the most red-shifted emission spectra both in solution and in the solid state, with maxima well beyond 600 nm. Indeed, thanks to their excellent emission features, compound **TPA-BDTO** and close derivatives thereof were widely applied by Tang’s and other groups in the bio-diagnostic and theranostic fields,^20−24^ as well as in high FQY PMMA dispersions for the fabrication of microlasers to be used in tensile strain sensors.^25^ On the other hand, compounds incorporating the BDT-tetraoxide core have never been reported as emitters for LSCs,^26^ and only the use of fluorophores based on the reduced BDT unit was described in the literature.^27^ For the above reasons, **TPA-BDTO** appeared as a promising candidate for use in LSCs, as well as an ideal starting point for developing other LSC fluorophores by introducing targeted modifications in its molecular structure.

Accordingly, in this paper, we report the design, synthesis, and spectroscopic characterization of a series of organic emitters featuring a benzo[1,2-*b*:4,5-*b*′]dithiophene 1,1,5,5-tetraoxide system as a central acceptor unit connected to different electron-donating and electron-withdrawing substituents. The spectroscopic properties of the compounds in organic solutions were investigated both in static conditions and by means of transient absorption techniques, to monitor the evolution of their excited states. Selected members of the series were then dispersed in PMMA films at different concentrations, and the optical and PV performances of the corresponding LSC were determined, together with a preliminary stability assessment by means of an accelerated aging protocol.

## 2 Experimental Section

### 2.1 General Synthetic and Experimental Remarks

All reactions carried out under an inert nitrogen atmosphere were performed in flame- or oven-dried glassware using Schlenk techniques. Tetrahydrofuran (THF) was purified by distillation over metallic sodium in the presence of benzophenone as an indicator, while aniline was distilled over KOH at 5 × 10^–2^ atm of pressure. Toluene, dichloromethane, *N*,*N*-dimethylformamide (DMF), diethylether, and acetonitrile were dried on a resin exchange Solvent Purification System (Innovative Technology) and stored under nitrogen over 4 Å molecular sieves. Degassing of solvents was carried out using the “freeze–pump–thaw” method. Compound **2b** was prepared according to a known procedure.^28^ Unless otherwise stated, all other chemicals employed were commercially available and were used as received. Petroleum ether was the 40–60 °C boiling fraction. Thin-layer chromatography (TLC) analyses were carried out using aluminum-supported silica gel plates containing a fluorescent indicator, and detection was carried out by exposure to UV light (λ = 254 and 365 nm) and/or treatment with permanganate or *p*-anisaldehyde solutions followed by heating. Flash column chromatography was performed using Merck Kieselgel 60 (300–400 mesh) as the stationary phase. ^1^H NMR spectra were recorded at 200, 300, or 400 MHz, and ^13^C NMR spectra were recorded at 100.6 MHz, with Bruker Avance or Varian Mercury series instruments. Chemical shifts are referenced to the residual solvent peak (CHCl_3_, δ 7.26 ppm for ^1^H NMR and δ 77.0 ppm for ^13^C NMR; THF-*d*_8_, δ 1.72 and 3.58 ppm for ^1^H NMR and δ 67.21 and 25.31 ppm for ^13^C NMR). Coupling constants (*J*) are expressed in Hz, while the used abbreviations are s (singlet), d (doublet), dd (doublet of doublets), t (triplet), td (triplet of doublets), and m (multiplet). Multiplets are indicated as chemical shift intervals. ESI-MS spectra were obtained by the direct injection of the sample solution, with a Thermo Scientific LCQ-FLEET instrument and are reported in the form *m*/*z*. UV/vis spectra in different solvents were recorded with a Shimadzu UV-2600 spectrometer. Fluorescence spectra on solutions were measured at room temperature with a Jasco FP-8300 spectrofluorometer equipped with a 450 W Xenon arc lamp, while quantum yield analysis was conducted using an ILF-835 integrating sphere (*ø* = 100 mm) connected to the instrument.

### 2.2 Experimental Synthetic Procedures

#### 2.2.1 4-Bromo-*N*,*N*-bis(4-(methylthio)phenyl)aniline (**2c**)

First step: In a Schlenk tube, Pd(OAc)_2_ (7 mg, 0.031 mmol, 0.03 equiv) and dppf (18 mg, 0.031 mmol, 0.03 equiv) were dissolved in dry toluene (10 mL), and the solution was kept stirring for 10 min under an inert atmosphere, until it turned orange. Then, aniline (100 mg, 1.04 mL, 1.1 mmol, 1.0 equiv), 4-bromothioanisole (478 mg, 2.32 mmol, 2.2 equiv) and, finally, *t*-BuONa (309 mg, 3.2 mmol, 3.0 equiv) were added. The resulting dark blue solution was heated up to 135 °C and stirred for 16 h, during which it turned yellow. The reaction mixture was cooled to room temperature, diluted with CH_2_Cl_2_, and washed with H_2_O (20 mL) and brine (20 mL). The combined organic layers were dried over Na_2_SO_4_ and concentrated under vacuum. The residue was purified by flash column chromatography (SiO_2_, petroleum ether/CH_2_Cl_2_ 6:1 to 4:1) to yield pure *N*,*N*-bis(4-(methylthio)phenyl)aniline (260 mg, 0.77 mmol, 70%) as a colorless oil. ^1^H NMR (CDCl_3_, 200 MHz): δ 7.22–7.25 (m, 2 H), 7.16 (dd, *J* = 8.6, 2.0 Hz, 4 H), 6.97–7.10 (m, 7 H), 2.47 (s, 6 H) ppm. The analytical data are in agreement with those reported in the literature.^29^

Second step: In a round-bottom flask, *N*,*N*-bis(4-(methylthio)phenyl)aniline (260 mg, 0.77 mmol, 1.0 equiv) was dissolved in DMF (5 mL), and the mixture was cooled to 0 °C. Then, a solution of *N*-bromosuccinimide (NBS, 137 mg, 0.77 mmol, 1.0 equiv) in DMF (5 mL) was added dropwise, and the resulting mixture was allowed to warm up to room temperature while shielded from the light. After 3 h, the reaction was quenched with H_2_O and extracted with EtOAc (3 × 10 mL). The combined organic layers were dried over Na_2_SO_4_ and concentrated under vacuum. The residue was purified by flash column chromatography (SiO_2_, petroleum ether/CH_2_Cl_2_ 6:1 to 4:1) to yield pure product **2c** (221 mg, 0.53 mmol, 69%) as a white solid. ^1^H NMR (CDCl_3_, 200 MHz): δ 7.32 (d, *J* = 8.7 Hz, 2 H), 7.17 (d, *J* = 8.7 Hz, 4 H), 6.99 (d, *J* = 8.7 Hz, 4 H), 6.92 (d, *J* = 8.7 Hz, 2 H), 2.47 (s, 6 H) ppm. The analytical data are in agreement with those reported in the literature.^29^

#### 2.2.2 General Procedure for the Symmetric Direct Arylation of Compound **1**

In a Schlenk tube, Pd_2_(dba)_3_ (0.05 equiv), P(*o*-MeOPh)_3_ (0.10 equiv), and pivalic acid (0.3 equiv) were dissolved in dry toluene (10 mL per 0.1 mmol of the substrate), and the mixture was kept under stirring for 10 min under an inert N_2_ atmosphere. Then, compound **1** (1.0 equiv), the appropriate aryl halide (**2a–c**, 2.2–4.0 equiv), and Cs_2_CO_3_ (2.4 equiv) were added, and the resulting suspension was heated up to 110 °C and stirred for 16 h. The reaction mixture was cooled to room temperature and filtered over a short pad of Celite^©^. Then, the solution was diluted with CH_2_Cl_2_ (equal volume of the reaction solvent) and washed with H_2_O (2 × volume of the reaction solvent) and brine (2 × volume of the reaction solvent). The combined organic layers were dried over Na_2_SO_4_ and concentrated under vacuum. The residue was purified by flash column chromatography to yield the pure product.

##### 2.2.2.1 2,6-Bis(4-(diphenylamino)phenyl)-4,8-bis((2-ethylhexyl)oxy)benzo[1,2-*b*:4,5-*b*′]dithiophene 1,1,5,5-Tetraoxide (**BDT-H2**)

Compound **BDT-H2** was synthesized from **1** (207 mg, 0.41 mmol) and 4-bromophenyl(diphenyl)amine (**2a**, 289 mg, 0.89 mmol) in accordance with the general procedure. The crude product was purified by flash column chromatography (SiO_2_, petroleum ether/CH_2_Cl_2_ 2:1 to 1:1), after which the desired compound was still contaminated by ca. 5% dibenzylideneacetone (dba). Recrystallization from a petroleum ether/CH_2_Cl_2_ 20:1 mixture yielded the pure product **BDT-H2** (227 mg, 0.23 mmol, 56%) as a deep purple solid. ^1^H NMR (CDCl_3_, 400 MHz): δ 7.65 (d, *J* = 8.6 Hz, 4 H), 7.28–7.35 (m, 8 H), 7.24 (s, 2 H), 7.05–7.19 (m, 16 H), 4.39 (d, *J* = 5.1 Hz, 4 H), 1.79–1.89 (m, 2 H), 1.46–1.67 (m, 8 H), 1.32–1.41 (m, 8 H), 1.00 (t, *J* = 7.4 Hz, 6 H), 0.92 (t, *J* = 6.4 Hz, 6 H) ppm. ^13^C NMR (CDCl_3_, 100 MHz): δ 150.0, 146.6, 145.0, 142.2, 131.3, 129.5, 127.6, 127.4, 125.6, 124.4, 121.7, 119.3, 115.0, 79.0, 40.4, 30.3, 29.0, 23.7, 23.0, 14.1, 11.2 ppm. ESI-MS: *m*/*z* calcd for C_62_H_64_N_2_O_6_S_2_ [M^+•^], 996.4; found, 996.1. The analytical data are in agreement with those reported in the literature.^18^

##### 2.2.2.2 2,6-Bis(4-(bis(4-methoxyphenyl)amino)phenyl)-4,8-bis((2-ethylhexyl)oxy)benzo[1,2-*b*:4,5-*b*′]dithiophene 1,1,5,5-Tetraoxide (**BDT-O2**)

Compound **BDT**-**O2** was synthesized from **1** (30 mg, 0.06 mmol) and **2b** (50 mg, 0.13 mmol) in accordance with the general procedure. The crude product was purified by flash column chromatography (SiO_2_, petroleum ether/CH_2_Cl_2_ 2:1 to 1:1) to yield pure product **BDT-O2** (84 mg, 0.075 mmol, 58%) as a deep purple solid. ^1^H NMR (CDCl_3_, 400 MHz): δ 7.58 (d, *J* = 8.9 Hz, 4 H), 7.16 (s, 2 H), 7.10 (d, *J* = 8.5 Hz, 8 H), 6.91 (d, *J* = 8.5 Hz, 4 H), 6.86 (d, *J* = 8.9 Hz, 8 H), 4.36 (d, *J* = 5.5 Hz, 4 H), 3.82 (s, 12 H), 1.79–1.89 (m, 2 H), 1.53–1.65 (m, 8 H), 1.31–1.40 (m, 8 H), 0.98 (t, *J* = 7.4 Hz, 6 H), 0.90 (t, *J* = 7.4 Hz, 6 H) ppm. ^13^C NMR (CDCl_3_, 100 MHz): δ 156.8, 143.3, 139.4, 133.5, 130.5, 129.0, 128.4, 127.5, 126.6, 125.4, 118.8, 117.5, 114.9, 78.9, 55.5, 40.4, 30.2, 29.7, 29.0, 23.7, 23.0, 14.1, 11.2 ppm. ESI-MS: *m*/*z* calcd for C_66_H_72_N_2_O_10_S_2_ [M^+•^], 1116.5; found, 1116.1.

##### 2.2.2.3 2,6-Bis(4-(bis(4-(methylthio)phenyl)amino)phenyl)-4,8-bis((2-ethylhexyl)oxy)benzo[1,2-*b*:4,5-*b*′]dithiophene 1,1,5,5-Tetraoxide (**BDT-S2**)

Compound **BDT-S2** was synthesized from **1** (50 mg, 0.10 mmol) and **2c** (92 mg, 0.22 mmol) in accordance with the general procedure. The crude product was purified by flash column chromatography (SiO_2_, petroleum ether/CH_2_Cl_2_ 2:1 to 1:2) to yield pure product **BDT-S2** (83 mg, 0.07 mmol, 70%) as a deep purple solid. ^1^H NMR (THF-*d*_8_, 400 MHz): δ 7.73 (d, *J* = 8.9 Hz, 4 H), 7.42 (s, 2 H), 7.24 (d, *J* = 8.6 Hz, 8 H), 7.09 (d, *J* = 8.5 Hz, 8 H), 7.06 (d, *J* = 8.9 Hz, 4 H), 4.41 (d, *J* = 5.8 Hz, 4 H), 2.46 (s, 12 H), 1.85–1.92 (m, 2 H), 1.54–1.60 (m, 8 H), 1.35–1.41 (m, 8 H), 1.01 (t, *J* = 7.4 Hz, 6 H), 0.84–0.96 (m, 6 H) ppm. ^13^C NMR (THF-*d*_8_, 100 MHz): δ 150.5, 145.8, 144.7, 143.4, 135.3, 132.9, 128.8, 128.4, 128.2, 126.6, 121.9, 120.6, 115.6, 79.6, 41.2, 32.7, 30.4, 24.3, 23.7, 15.9, 14.2, 11.3 ppm. ESI-MS: *m*/*z* calcd for C_66_H_73_N_2_O_6_S_6_ [M + H^+•^], 1181.4; found, 1181.3.

##### 2.2.2.4 4,4′-(4,8-Bis((2-ethylhexyl)oxy)-1,1,5,5-tetraoxidobenzo[1,2-*b*:4,5-*b*′]dithiophene-2,6-diyl)dibenzonitrile (**BDT-AA**)

Compound **BDT-AA** was synthesized from **1** (50 mg, 0.10 mmol) and 4-bromobenzonitrile (**3**, 72 mg, 0.40 mmol) in accordance with the general procedure. The crude product was purified by flash column chromatography (SiO_2_, petroleum ether/CH_2_Cl_2_ 1:1 to sole CH_2_Cl_2_) to yield product **BDT-AA** (50 mg, 0.07 mmol, 70%) as an orange solid. ^1^H NMR (CDCl_3_, 400 MHz): δ 7.92 (d, *J* = 8.4 Hz, 4 H), 7.79 (d, *J* = 8.4 Hz, 4 H), 7.57 (s, 2 H), 4.47 (d, *J* = 5.5 Hz, 4 H), 1.84–1.92 (m, 2 H), 1.46–1.66 (m, 8 H), 1.33–1.43 (m, 8 H), 1.01 (t, *J* = 7.4 Hz, 6 H), 0.93 (t, *J* = 7.1 Hz, 6 H) ppm. ^13^C NMR (CDCl_3_, 100 MHz): δ 145.4, 140.9, 133.0, 131.2, 127.5, 127.2, 121.1, 117.9, 114.2, 110.0, 79.2, 40.3, 30.3, 29.0, 23.7, 23.0, 14.1, 11.1 ppm. ESI-MS: *m*/*z* calcd for C_80_H_88_N_4_NaO_12_S_4_ [2M + Na^+•^], 1447.5; found, 1446.8.

#### 2.2.3 2-(4-(Diphenylamino)phenyl)-4,8-bis((2-ethylhexyl)oxy)benzo[1,2-*b*:4,5-*b*′]dithiophene 1,1,5,5-Tetraoxide (**4a**)

In a Schlenk tube, Pd_2_(dba)_3_ (4 mg, 0.004 mmol, 0.05 equiv), P(*o*-MeOPh)_3_ (6 mg, 0.016 mmol, 0.2 equiv), and pivalic acid (2 mg, 0.023 mmol, 0.3 equiv) were dissolved in dry toluene (5 mL), and the mixture was kept stirring for 10 min under an inert atmosphere. Then, **1** (40 mg, 0.078 mmol, 1.0 equiv), **2a** (25 mg, 0.078 mmol, 1.0 equiv), and Cs_2_CO_3_ (31 mg, 0.094 mmol, 1.2 equiv) were added, and the resulting suspension was heated up to 80 °C. After 16 h, the suspension was diluted with CH_2_Cl_2_ (10 mL) and washed with H_2_O (2 × 10 mL) and brine (10 mL). The combined organic layers were dried over Na_2_SO_4_ and concentrated under vacuum. The crude product was purified by flash column chromatography (SiO_2_, petroleum ether/CH_2_Cl_2_ 2:1 to 1:1) to yield product **4a** (20 mg, 0.027 mmol, 34%) as a deep purple solid. ^1^H NMR (CDCl_3_, 400 MHz): δ 7.65 (d, *J* = 8.6 Hz, 2 H), 7.38 (d, *J* = 7.0 Hz, 1 H), 7.27–7.35 (m, 4 H), 7.23 (s, 1 H), 7.03–7.20 (m, 8 H), 6.65 (d, *J* = 7.0 Hz, 1 H), 4.39 (d, *J* = 5.6 Hz, 2 H), 4.36 (d, *J* = 5.6 Hz, 2 H), 1.76–1.85 (m, 2 H), 1.42–1.63 (m, 8 H), 1.31–1.38 (m, 8 H), 0.95–1.02 (m, 6 H), 0.87–0.95 (m, 6 H) ppm. ^13^C NMR (CDCl_3_, 100 MHz): δ 150.3, 146.6, 145.3, 145.0, 143.1, 131.4, 131.2, 130.0, 129.8, 129.7, 127.8, 127.5, 126.0, 125.8, 124.6, 121.6, 119.1, 114.5, 79.1, 78.9, 40.5, 30.4, 30.3, 29.2, 29.1, 23.9, 23.8, 23.1, 14.2, 11.3 ppm. ESI-MS: *m*/*z* calcd for C_44_H_51_NO_6_S_2_ [M + H^+•^], 754.3; found, 754.3.

#### 2.2.4 4-(6-(4-(Diphenylamino)phenyl)-4,8-bis((2-ethylhexyl)oxy)-1,1,5,5-tetraoxidobenzo[1,2-*b*:4,5-*b*′]dithiophen-2-yl)benzonitrile (**BDT-H1**)

In a Schlenk tube, Pd_2_(dba)_3_ (1.4 mg, 0.0013 mmol, 0.05 equiv), P(*o*-MeOPh)_3_ (2 mg, 0.0054 mmol, 0.2 equiv), and pivalic acid (1 mg, 0.008 mmol, 0.3 equiv) were dissolved in dry toluene (5 mL), and the mixture was stirred for 10 min under an inert atmosphere. Then, compound **4a** (20 mg, 0.027 mmol, 1.0 equiv), 4-bromobenzonitrile (**3**, 6 mg, 0.032 mmol, 1.2 equiv), and Cs_2_CO_3_ (10 mg, 0.032 mmol, 1.2 equiv) were added, and the resulting suspension was heated up to 110 °C and stirred under an inert atmosphere for 16 h. The suspension was diluted with CH_2_Cl_2_ (10 mL) and washed with H_2_O (2 × 10 mL) and brine (10 mL). The combined organic layers were dried over Na_2_SO_4_ and concentrated under vacuum. The crude product was purified by flash column chromatography (SiO_2_, petroleum ether/CH_2_Cl_2_ 2:1 to 1:1) to yield product **BDT-H1** (6 mg, 0.007 mmol, 26%) as a deep purple solid. ^1^H NMR (CDCl_3_, 400 MHz): δ 7.91 (d, *J* = 8.6 Hz, 2 H), 7.77 (d, *J* = 8.2 Hz, 2 H), 7.65 (d, *J* = 8.6 Hz, 2 H), 7.55 (s, 1 H), 7.29–7.35 (m, 4 H), 7.24 (s, 1 H), 7.05–7.18 (m, 8 H), 4.46 (d, *J* = 5.5 Hz, 2 H), 4.39 (d, *J* = 5.5 Hz, 2 H), 1.82–1.91 (m, 2 H), 1.54–1.65 (m, 8 H), 1.32–1.44 (m, 8 H), 1.01 (t, *J* = 7.4 Hz, 3 H), 1.00 (t, *J* = 7.4 Hz, 3 H), 0.88–0.98 (m, 6 H) ppm. ^13^C NMR (CDCl_3_, 100 MHz): δ 150.4, 146.4, 145.6, 144.8, 143.4, 143.1, 139.9, 133.0, 131.2, 130.5, 129.6, 129.0, 128.4, 127.8, 127.0, 125.8, 125.1, 124.6, 121.6, 121.4, 118.7, 118.1, 114.2, 79.1, 79.0, 40.4, 30.3, 29.0, 23.7, 23.0, 14.1, 11.2 ppm. ESI-MS: *m*/*z* calcd for C_51_H_54_N_2_O_6_S_2_ [M^+•^], 854.3; found, 854.1.

#### 2.2.5 4-(6-(4-(Diphenylamino)phenyl)-4,8-bis((2-ethylhexyl)oxy)-1,1,5,5-tetraoxidobenzo[1,2-*b*:4,5-*b*′]dithiophen-2-yl)benzonitrile (**BDT-H1**)—One-Pot Procedure

In a Schlenk tube, Pd_2_(dba)_3_ (10 mg, 0.009 mmol, 0.05 equiv), P(*o*-MeOPh)_3_ (6 mg, 0.018 mmol, 0.10 equiv), and pivalic acid (6 mg, 0.054 mmol, 0.3 equiv) were dissolved in dry toluene (5 mL), and the resulting mixture was stirred for 10 min under an inert atmosphere. Then, **1** (94 mg, 0.18 mmol, 1.0 equiv), 4-bromophenyl(diphenyl)amine (**2a**, 60 mg, 0.18 mmol, 1.0 equiv), and Cs_2_CO_3_ (72 mg, 0.22 mmol, 1.2 equiv) were added, and the resulting suspension was heated up to 80 °C. After the aryl halide was consumed according to a TLC check (approx. 16 h of reaction, eluent petroleum ether/CH_2_Cl_2_ 2:1 to 1:1), a second aliquot of Cs_2_CO_3_ (72 mg, 0.22 mmol, 1.2 equiv) was added to the mixture, followed by 4-bromobenzonitrile (**3**, 50 mg, 0.28 mmol, 1.5 equiv). The temperature was raised to 110 °C, and the reaction was stirred under an inert atmosphere for an additional 16 h. Then, the suspension was diluted with CH_2_Cl_2_ (10 mL) and washed with H_2_O (2 × 10 mL) and brine (10 mL). The combined organic layers were dried over Na_2_SO_4_ and concentrated under vacuum. The residue was purified by flash column chromatography (SiO_2_, petroleum ether/CH_2_Cl_2_ 2:1 to 1:1) to yield product **BDT-H1** (43 mg, 0.05 mmol, 28%) as a deep purple solid. The spectroscopic characterization was identical to that of the product obtained via the two-step procedure (see above, paragraph 2.2.4).

#### 2.2.6 2-(4-(Bis(4-methoxyphenyl)amino)phenyl)-4,8-bis((2-ethylhexyl)oxy)benzo[1,2-*b*:4,5-*b*′]dithiophene 1,1,5,5-Tetraoxide (**4b**)

In a Schlenk tube, Pd_2_(dba)_3_ (3 mg, 0.003 mmol, 0.05 equiv), P(*o*-MeOPh)_3_ (4 mg, 0.012 mmol, 0.2 equiv), and pivalic acid (2 mg, 0.018 mmol, 0.3 equiv) were dissolved in dry toluene (5 mL), and the mixture was kept stirring for 10 min under an inert atmosphere. Then, **1** (30 mg, 0.06 mmol, 1.0 equiv), **2b** (23 mg, 0.06 mmol, 1.0 equiv), and Cs_2_CO_3_ (23 mg, 0.07 mmol, 1.2 equiv) were added, and the resulting suspension was heated up to 80 °C. After 16 h, the suspension was diluted with CH_2_Cl_2_ (10 mL) and washed with H_2_O (2 × 10 mL) and brine (10 mL). The combined organic layers were dried over Na_2_SO_4_ and concentrated under vacuum. The crude product was purified by flash column chromatography (SiO_2_, petroleum ether/CH_2_Cl_2_ 2:1 to 1:1) to yield product **4b** (19 mg, 0.023 mmol, 38%) as a deep purple solid. ^1^H NMR (CDCl_3_, 400 MHz): δ 7.59 (d, *J* = 8.4 Hz, 2 H), 7.37 (d, *J* = 6.9 Hz, 1 H), 7.15 (s, 1 H), 7.02–7.13 (m, 4 H), 6.80–7.00 (m, 6 H), 6.64 (d, *J* = 6.9 Hz, 1 H), 4.38 (d, *J* = 5.4 Hz, 2 H), 4.34 (d, *J* = 5.4 Hz, 2 H), 3.81 (s, 6 H), 1.76–1.85 (m, 2 H), 1.46–1.63 (m, 8 H), 1.29–1.41 (m, 8 H), 0.98 (t, *J* = 7.4 Hz, 3 H), 0.97 (t, *J* = 7.4 Hz, 3 H), 0.87–0.93 (m, 6 H) ppm. ^13^C NMR (CDCl_3_, 100 MHz): δ 157.0, 151.1, 145.3, 144.9, 143.5, 139.3, 131.4, 131.1, 130.7, 130.2, 129.8, 129.1, 128.5, 127.7, 125.6, 118.9, 115.0, 113.3, 79.1, 78.9, 55.6, 40.5, 31.1, 30.4, 29.2, 23.8, 23.2, 14.2, 11.3 ppm. ESI-MS: *m*/*z* calcd for C_46_H_56_NO_8_S_2_ [M + H^+•^], 814.3; found, 814.2.

#### 2.2.7 4-(6-(4-(Bis(4-methoxyphenyl)amino)phenyl)-4,8-bis((2-ethylhexyl)oxy)-1,1,5,5-tetraoxidobenzo[1,2-*b*:4,5-*b*′]dithiophen-2-yl)benzonitrile (**BDT-O1**)

In a Schlenk tube, Pd_2_(dba)_3_ (6 mg, 0.006 mmol, 0.05 equiv), P(*o*-MeOPh)_3_ (8 mg, 0.024 mmol, 0.2 equiv), and pivalic acid (4 mg, 0.035 mmol, 0.3 equiv) were dissolved in dry toluene (10 mL), and the mixture was stirred for 10 min under an inert atmosphere. Then, compound **4b** (94 mg, 0.12 mmol, 1.0 equiv), 4-bromobenzonitrile (**3**, 33 mg, 0.18 mmol, 1.5 equiv), and Cs_2_CO_3_ (16 mg, 0.048 mmol, 2.4 equiv) were added, and the resulting suspension was heated up to 110 °C and stirred under an inert atmosphere for 16 h. Then, the suspension was diluted with CH_2_Cl_2_ (10 mL) and washed with H_2_O (2 × 10 mL) and brine (10 mL). The combined organic layers were dried over Na_2_SO_4_ and concentrated under vacuum. The crude product was purified by flash column chromatography (SiO_2_, petroleum ether/CH_2_Cl_2_ 2:1 to 1:1) to yield product **BDT-O1** (80 mg, 0.088 mmol, 73%) as a deep purple solid. ^1^H NMR (CDCl_3_, 400 MHz): δ 7.90 (d, *J* = 8.6 Hz, 2 H), 7.75–7.78 (m, 2 H), 7.72 (s, 1 H), 7.58–7.66 (m, 4 H), 7.54 (s, 1 H), 7.38–7.48 (m, 4), 6.86–6.92 (m, 4 H), 4.46 (d, *J* = 5.5 Hz, 2 H), 4.37 (d, *J* = 5.5 Hz, 2 H), 3.84 (bs, 6 H), 1.81–1.90 (m, 2 H), 1.48–1.64 (m, 8 H), 1.33–1.42 (m, 8 H), 0.98–1.01 (m, 6 H), 0.90–0.94 (m, 6 H) ppm. ^13^C NMR (CDCl_3_, 100 MHz): δ 145.6, 143.3, 139.8, 134.9, 134.8, 132.9, 131.3, 130.5, 129.0, 128.4, 127.8, 127.6, 127.0, 125.4, 124.7, 121.6, 118.7, 118.1, 115.0, 114.9, 114.8, 113.6, 113.0, 79.1, 79.0, 55.5, 40.4, 30.3, 29.7, 29.0, 23.7, 23.0, 14.1, 11.2 ppm. ESI-MS: *m*/*z* calcd for C_53_H_58_N_2_O_8_S_2_ [M^+•^], 914.4; found, 914.1.

#### 2.2.8 2-(4-(Bis(4-(methylthio)phenyl)amino)phenyl)-4,8-bis((2-ethylhexyl)oxy)benzo[1,2-*b*:4,5-*b*′]dithiophene 1,1,5,5-Tetraoxide (**4c**)

In a Schlenk tube, Pd_2_(dba)_3_ (5 mg, 0.005 mmol, 0.05 equiv), P(*o*-MeOPh)_3_ (7 mg, 0.02 mmol, 0.2 equiv), and pivalic acid (3 mg, 0.03 mmol, 0.3 equiv) were dissolved in dry toluene (5 mL), and the mixture was stirred for 10 min under an inert atmosphere. Then, **1** (50 mg, 0.1 mmol, 1.0 equiv), **2c** (42 mg, 0.1 mmol, 1.0 equiv), and Cs_2_CO_3_ (65 mg, 0.2 mmol, 2.0 equiv) were added, and the resulting suspension was heated up to 80 °C and stirred for 16 h. Then, the suspension was diluted with CH_2_Cl_2_ (10 mL) and washed with H_2_O (2 × 10 mL) and brine (2 × 10 mL). The combined organic layers were dried over Na_2_SO_4_ and concentrated under vacuum. The residue was purified by flash column chromatography (SiO_2_, petroleum ether/CH_2_Cl_2_ 2:1 to 1:2) to yield product **4c** (31 mg, 0.036 mmol, 36%) as a deep purple solid. ^1^H NMR (CDCl_3_, 400 MHz): δ 7.63 (d, *J* = 9.0 Hz, 2 H), 7.37 (d, *J* = 6.6 Hz, 1 H), 7.22 (s, 1 H), 7.20 (d, *J* = 8.6 Hz, 4 H), 7.03–7.08 (m, 6 H), 6.66 (d, *J* = 7.0 Hz, 1 H), 4.38 (d, *J* = 5.5 Hz, 2 H), 4.35 (d, *J* = 5.5 Hz, 2 H), 2.48 (s, 6 H), 1.76–1.85 (m, 2 H), 1.43–1.62 (m, 8 H), 1.31–1.39 (m, 8 H), 0.98 (t, *J* = 7.4 Hz, 3 H), 0.97 (t, *J* = 7.4 Hz, 3 H), 0.87–0.93 (m, 6 H) ppm. ^13^C NMR (CDCl_3_, 100 MHz): δ 149.8, 145.2, 144.9, 143.7, 142.9, 134.1, 131.2, 131.0, 129.8, 129.7, 128.2, 127.8, 127.4, 126.0, 125.9, 121.3, 119.0, 114.5, 79.0, 78.9, 40.3, 30.3, 30.2, 29.01, 28.96, 23.7, 23.6, 23.0, 16.4, 14.10, 14.07, 11.1 ppm. ESI-MS: *m*/*z* calcd for C_46_H_55_NO_6_S_4_ [M^+•^], 845.3; found, 845.0.

#### 2.2.9 4-(6-(4-(Bis(4-(methylthio)phenyl)amino)phenyl)-4,8-bis((2-ethylhexyl)oxy)-1,1,5,5-tetraoxidobenzo[1,2-*b*:4,5-*b*′]dithiophen-2-yl)benzonitrile (**BDT-S1**)

In a Schlenk tube, Pd_2_(dba)_3_ (6 mg, 0.006 mmol, 0.05 equiv), P(*o*-MeOPh)_3_ (8 mg, 0.024 mmol, 0.20 equiv), and pivalic acid (4 mg, 0.036 mmol, 0.3 equiv) were dissolved in dry toluene (10 mL), and the mixture was stirred for 10 min under an inert atmosphere. Then, **4c** (100 mg, 0.12 mmol, 1.0 equiv), 4-bromobenzonitrile (**3**, 88 mg, 0.48 mmol, 0.4 equiv), and Cs_2_CO_3_ (78 mg, 0.24 mmol, 2.0 equiv) were added, and the resulting suspension was heated up to 110 °C and stirred under an inert atmosphere for 16 h. Then, the suspension was diluted with CH_2_Cl_2_ (10 mL) and washed with H_2_O (2 × 10 mL) and brine (2 × 10 mL). The combined organic layers were dried over Na_2_SO_4_ and concentrated under vacuum. The residue was purified by flash column chromatography (SiO_2_, petroleum ether/CH_2_Cl_2_ 2:1 to 1:3) to yield product **BDT-S1** (80 mg, 0.085 mmol, 70%) as a deep purple solid. ^1^H NMR (CDCl_3_, 400 MHz): δ 7.91 (d, *J* = 8.6 Hz, 2 H), 7.77 (d, *J* = 8.6 Hz, 2 H), 7.61–7.69 (m, 2 H), 7.55 (s, 1 H), 7.17–7.25 (m, 5 H), 6.95–7.13 (m, 6 H), 4.45 (d, *J* = 5.5 Hz, 2 H), 4.38 (d, *J* = 5.5 Hz, 2 H), 2.51 (bs, 6 H), 1.81–1.91 (m, 2 H), 1.50–1.65 (m, 8 H), 1.33–1.41 (m, 8 H), 1.00 (t, *J* = 7.4 Hz, 3 H), 0.99 (t, *J* = 7.4 Hz, 3 H), 0.88–0.95 (m, 6 H) ppm. ^13^C NMR (CDCl_3_, 100 MHz): δ 145.6, 144.8, 143.4, 139.9, 132.9, 131.3, 131.2, 130.9, 130.5, 129.7, 128.9, 128.4, 128.0, 127.9, 127.7, 126.9, 126.2, 125.4, 125.2, 121.5, 118.1, 113.7, 108.5, 79.1, 78.9, 40.3, 30.2, 29.0, 23.7, 23.1, 16.3, 14.1, 11.2 ppm. ESI-MS: *m*/*z* calcd for C_53_H_58_N_2_O_6_S_4_ [M^+•^], 946.3; found, 946.3.

### 2.3 Femtosecond Transient Absorption Spectroscopy Experiments

The apparatus used for the transient absorption spectroscopy (TAS) measurements has been described in detail before. Briefly, 40 fs pulses centered at 800 nm were produced by an integrated Ti/sapphire oscillator (Micra-Coherent) coupled with a regenerative amplifier system (Legend-Coherent). The excitation wavelength was set at 500 or 400 nm, and excitation power was set at about 200 nJ for all measurements. Visible pulses at 500 nm were generated by frequency mixing the idler and signal output produced by pumping a commercial optical parametric amplifier (TOPAS, Light Conversion) with a portion of the fundamental 800 nm radiation in a β-barium borate (BBO) crystal. Excitation pulses at 400 nm were obtained by the second harmonic generation of the fundamental laser radiation using a 2 mm BBO crystal. The pump beam polarization has been set to magic angle with respect to the probe beam by rotating a λ/2 plate to exclude rotational contributions.^30−32^ The white light probe pulse was generated by focusing a small portion of the fundamental laser radiation on a 3 mm thick CaF_2_ window. A portion of the generated white light was sent to the sample through a different path and used as a reference signal. After passing through the sample, the white light probe and reference pulses were both directed to a flat field monochromator coupled to a home-made detector. Transient signals were acquired in a time interval spanning up to 1500 ps. The sample was contained in a 2 mm quartz cuvette, mounted on a movable holder in order to minimize photodegradation. Measurements were performed at room temperature. Concentrations were adjusted to an absorbance of 0.9–1.0 OD (for the respective optical path) at the absorption maximum which amounted to about 0.3–0.5 OD at the excitation wavelength. Before and after the measurements, the integrity of the sample was checked on a PerkinElmer LAMBDA 950 spectrophotometer. Data analysis has been performed applying a global analysis procedure^33^ using software GLOTARAN^34^ and employing a linear unidirectional kinetic scheme. Global analysis allows the simultaneous fit of all the measured wavelengths with a combination of exponential decay functions and retrieves the kinetic constants describing the dynamic evolution of the system and the corresponding spectral component, called evolution-associated difference spectra (EADS).

### 2.4 LSC Preparation and Film Characterization

Fluorophore/PMMA thin films were prepared by drop casting according to the following procedure. Chloroform solutions (1.5 mL) containing 60 mg of the polymer and the proper amount of the fluorophore to obtain concentrations in the range 0.4–2.8 wt % were poured on a 50 × 50 × 3 mm^3^ optically pure glass substrate (Edmund Optics Ltd. BOROFLOAT window 50 × 50 TS). The glass slides were cleaned with chloroform and immersed in 6 M HCl for at least 12 h, and were then rinsed with water, acetone, and isopropanol. The resulting LSCs were stored for 24 h to allow the solvent to evaporate completely. The film thickness was measured by a Starrett micrometer to be 25 ± 5 μm. After LSC characterization, the polymer films were carefully detached from the glass surface by immersing the LSC in water, stored in a desiccator, and then analyzed by means of absorption and emission spectroscopies.

UV/vis spectra were recorded on polymer films at room temperature with a Cary 5000 UV–Vis–NIR spectrophotometer (Agilent). The fluorescence spectra were measured on polymer films at room temperature with a Fluorolog-3 spectrofluorometer (Horiba Jobin-Yvon, Horiba Italy) equipped with a 450 W Xenon arc lamp and double-grating excitation and single-grating emission monochromators. Quantum yield measurements were carried out using an external integration sphere (Quanta-ϕ F-3029, Horiba), equipped with solid or liquid sample holder, and connected to the spectrofluorometer by optical fibers and a fiber-optics adaptor (FL-3000, Horiba). Photographs of the film emissions under illumination were obtained by placing them in a Dark Reader 46B transilluminator equipped with a 450 nm LED source (Clare Chemical Research).

### 2.5 Optical Efficiency

Internal (η_int_) and external (η_ext_) photon and device (η_dev_) efficiencies were determined according to recently agreed protocols, and details are reported in the Supporting Information.^35,36^ η_int_ and η_ext_ were measured by using a commercially available system (Arkeo, Cicci research *s.r.l.*, Grosseto, Italy) containing a complementary metal-oxide semiconductor (CMOS)-based spectrometer with a symmetric Czerny–Turner optical bench connected to an integrating sphere as reported in a recent publication.^16^ As an illumination source, an ORIEL LCS-100 solar simulator 94011A S/N: 322 was utilized under controlled illumination (1 sun, AM 1.5G). For the determination of η_dev_, two PV cells IXYS KXOB25-12 × 1F (22 × 7 mm, *V*_oc_ = 0.69 V, *I*_sc_ = 46.7 mA, FF > 70%, and PCE = 25%) were connected in series, and the current/voltage characteristics were determined with a precision source/measure unit (B2900 Series, Keysight Technologies). Silicon was used to grease the LSC edge. The other three edges of the LSC were covered with a reflective aluminum tape. A black matte layer was placed beneath the LSC with an air gap of about 2.5 mm during the measurements.

## 3 Results and Discussion

### 3.1 Design of the Emitters

Before delving into the description of the emitters’ structures, we point out that, from here on, fluorophore **TPA-BDTO** will be denoted as **BDT-H2** so that a homogeneous nomenclature can be used across the entire series of compounds, facilitating the immediate identification of their structural differences.

The first set of structures was designed based on a symmetric architecture. Considering the marked electron-withdrawing character of the central BDT-tetraoxide core, attaching two electron-donating triarylamine (TAA) moieties on both of its sides gives rise to a D–A–D arrangement. We reasoned that the electron-donating ability of the TAA group could be modulated by the introduction of different substituents on the 4′-position of the terminal phenyl rings, namely, a methoxy- or a thiomethyl-group, giving compounds **BDT-O2** and **BDT-S2**, respectively (Figure 1, left). As mentioned earlier, D–A–D compounds of this kind were expected to give rise to an intramolecular charge transfer (ICT) upon photoexcitation, helping achieve a polar excited state with emission in the red/NIR region and a large Stokes shift.

We also envisioned a second array of compounds based on a non-symmetric structure, in which the same donor groups were introduced only on one side of the molecules, while on the other an additional electron-withdrawing cyanobenzene unit was placed, yielding a D–A–A′ architecture (**BDT-H1,O1,S1**, Figure 1, right). Such arrangement was investigated because it was reported to enhance the bathochromic shift of the emission, while retaining moderate-to-good FQY, at least in less polar solvents.^37,38^ Furthermore, although non-symmetric emitters have been previously employed in LSCs,^39−42^ to the best of our knowledge a direct comparison between the performances of devices built with symmetric and non-symmetric analogues, measured under the same conditions, is still missing, probably due to the more complicated synthetic procedures usually required to prepare the latter.

Finally, to check the effect of the foreseen ICT on the compound properties, a further symmetric emitter (named, **BDT-AA**, Figure 1, bottom) was also designed as a reference, i.e., by introducing two cyanobenzene units at the sides of the central BDT-tetraoxide system. Indeed, its peculiar A′–A–A′ arrangement should not be capable of yielding ICT transitions, thus providing a potentially interesting comparison to the other compounds featuring alternating donor–acceptor moieties.

### 3.2 Computational Investigation

**Figure 2:**
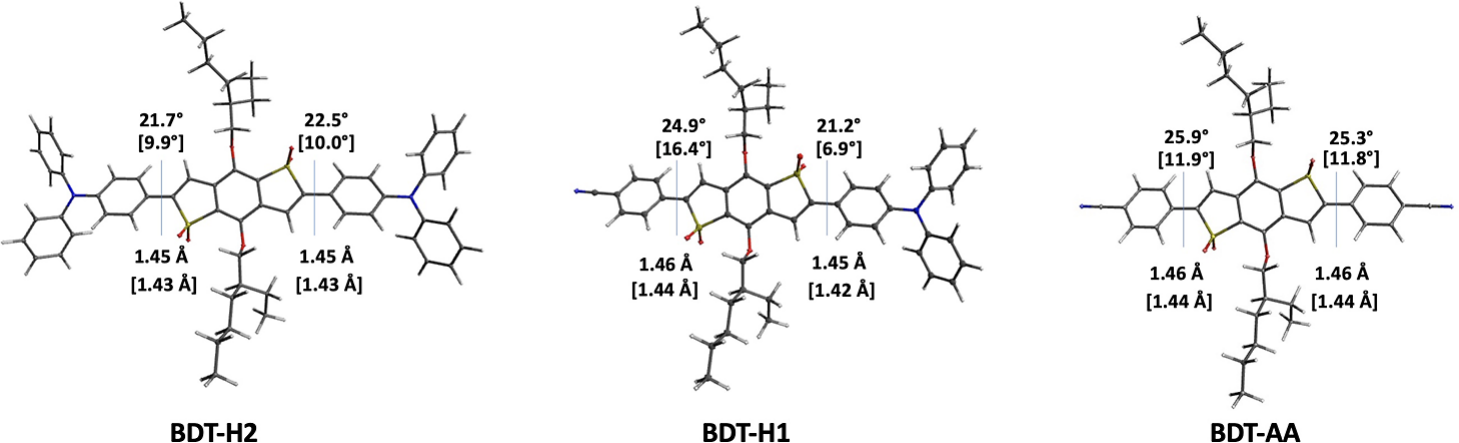
Bond lengths (Å) and dihedral angles (degrees) of S_0_- and S_1_ (in brackets)-optimized geometries of compounds **BDT-H2**, **BDT-H1**, and **BDT-AA**.

To assess if the photophysical properties of the designed compounds could be suitable for their employment in LSCs, we carried out a computational investigation based on density functional theory (DFT)^43,44^ and time-dependent DFT (TD-DFT)^45,46^ methods, using Gaussian 16, Revision C.01 suite of programs.^47^ The S_0_-optimized geometries have been obtained at the B3LYP/6-31G* level of theory^48,49^ in vacuo, while the S_1_-optimized geometries have been computed at the TD-CAM-B3LYP/6-31G* level of theory^50^ including the effects of the chosen solvent, toluene (Figure 2, where only those of compounds **BDT-H2**, **BDT-H1**, and **BDT-AA** are shown for brevity; for the complete series, see Figure S1, Supporting Information).

In the ground state, dihedral angles of 21–26° were found between the central oxidized BDT core and the flanking phenyl rings of all compounds, leading to a partial loss of co-planarity of their structures. This can explain the relatively small differences observed in the experimental absorption spectra of the compounds in different solvents (see below) as the torsion limits the dipole moment of the molecules in the ground state.^38^ As already observed for other classes of emitters,^15^ in the first excited state the torsional angles were reduced to values comprised between 6 and 17° and thus were expected to favor the electronic delocalization across the molecules, which should be visible in the corresponding emission spectra.

**Figure 3:**
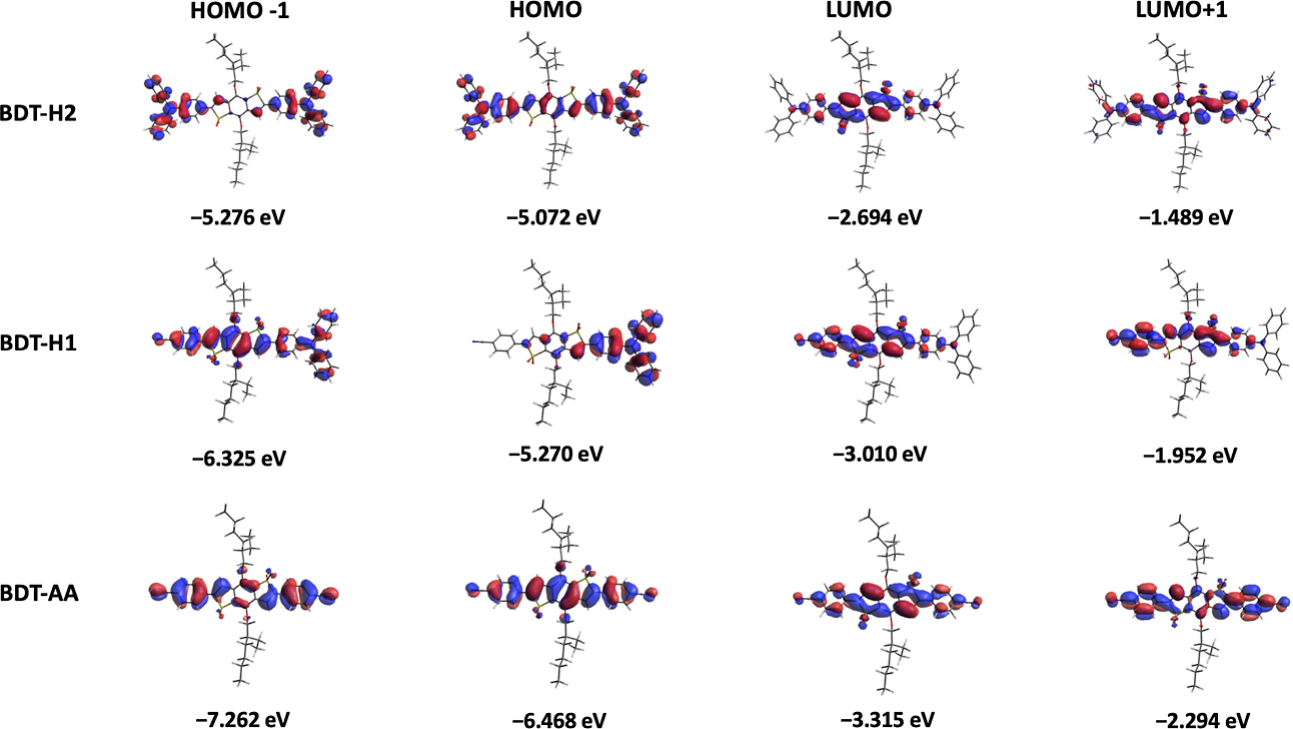
DFT (B3LYP/6-31G*) ground-state FMOs of **BDT-H2, H1**, and **AA** in toluene, with indication of their computed energies (see Table S1).

The ground-state energies of frontier molecular orbitals (FMOs) and their electron density distributions were assessed by means of DFT calculations at the B3LYP/6-31G* level of theory including the solvent effects (Table S1 and Figure 3, where, once again, only compounds **BDT-H2**, **BDT-H1**, and **BDT-AA** are shown; for the complete series, see Figure S2). In terms of energy, the destabilizing effect of the stronger donor groups on the occupied orbitals (and in particular the HOMOs) is evident in both the symmetric and non-symmetric emitter series, while the effect on the LUMOs was less pronounced. Remarkably, the energy levels of non-symmetric compounds were all clearly stabilized due to the presence of the additional benzonitrile acceptor; such an effect was even more pronounced for compound **BDT-AA** featuring two lateral EWG groups.

Concerning the electron density distribution, in the case of the D–A–D compounds, the HOMOs were mostly localized on the lateral donor groups, with a minor contribution of the central core, while the LUMOs were centered on the acceptor BDT unit. A similar tendency was also observed for the non-symmetric compounds, although in such cases, the HOMOs were mainly located on their single donor unit, while the LUMOs were widely delocalized over both the BDT central core and the additional acceptor. As expected, the frontier orbitals of **BDT-AA** did not present the same features since they all stretched along the entire conjugated scaffold of the molecule.

TD-MPW1K^51^/6-311+G(2d,p) absorption maxima (λ_cal_^abs,max^), vertical excitation (*E*_exc_) energies, oscillator strengths (*f*), and composition (%) in terms of molecular orbitals for the lowest singlet–singlet excitations (S_0_ → S_1_) were then calculated and are reported in Table 1. Solvent effects have been included by using the polarizable continuum model (PCM).^52^

**Table 1:** TD-MPW1K/6-311+G(2d,p) Absorption Maxima (λ_cal_^abs,max^ in nm), Excitation Energies (*E*_exc_ in eV), Oscillator Strengths (*f*), and Orbital Contributions (%) to the Lowest Energy Transition of **BDT** Compounds in Toluene Solution

compound	λ_cal_^abs,max^ [nm]	*E*_exc_ [eV]	*f*	contribution [%]
**BDT-H2**	501	2.48	1.64	85 (H → L)
**BDT-O2**	524	2.37	1.67	86 (H → L)
**BDT-S2**	521	2.38	1.70	83 (H → L)
**BDT-H1**	496	2.50	1.27	82 (H → L)
**BDT-O1**	525	2.36	1.22	84 (H → L)
**BDT-S1**	521	2.38	1.18	82 (H → L)
**BDT-AA**	420	2.95	1.21	94 (H → L)

For D–A–D and D–A–A′ compounds, λ_cal_^abs,max^ values were comprised between 496 and 525 nm, while a shorter wavelength was calculated for **BDT-AA**. Importantly, for all compounds the lowest energy excitations were largely associated with the HOMO–LUMO transitions (>80%). Given the overall FMOs electron density described above, this supported the hypothesis of an ICT photoexcitation process in the BDT series of compounds, except for **BDT-AA**, whose frontier orbitals were evenly distributed along the molecule due to its different structure.

To evaluate the emission behavior of the compounds, the first excited-state FMO energies and spatial distributions were then assessed in toluene at the TD-CAM-B3LYP/6-31G* level (Table S2 and Figure S3), revealing the same qualitative features already observed for the ground state, possibly with a slightly more pronounced delocalization, as a consequence of the more planar structures. Then, emission maxima (λ_cal_^emi,max^), vertical emission (*E*_emi_) energies, oscillator strengths (*f*), and composition (%) in terms of molecular orbitals for the lowest singlet–singlet emissions (S_1_ → S_0_) were calculated, following a previously developed computational protocol,^53^ at the TD-MPW1K/6-311+G(2d,p) level, using the linear-response implementation (LR-PCM) (Table 2).

**Table 2:** TD-MPW1K/6-311+G(2d,p) Emission Maxima (λ_cal_^emi,max^ in nm), Emission Energies (*E*_emi_ in eV), Oscillator Strengths (*f*), and Orbital Contributions (%) to the Lowest Energy Transition of **BDT** Compounds in Toluene Solution

compound	λ_cal_^emi,max^ [nm]	*E*_emi_ [eV]	*f*	contribution [%]
**BDT-H2**	644	1.92	1.66	92 (L → H)
**BDT-O2**	667	1.86	1.72	91 (L → H)
**BDT-S2**	663	1.87	1.78	86 (L → H)
**BDT-H1**	626	1.98	1.46	90 (L → H)
**BDT-O1**	653	1.90	1.45	88 (L → H)
**BDT-S1**	649	1.91	1.47	86 (L → H)
**BDT-AA**	571	2.17	1.23	96 (L → H)

Donor–acceptor compounds provided computed maximum emission values well above 600 nm, in agreement with expectations, with a very pronounced effect of the more electron-donating side groups. Once again, except for **BDT-AA**, the lowest energy emissions were mostly associated with ICT transitions involving the two frontier orbitals (LUMO → HOMO), in good agreement with the shifts in the maximum values and the solvatochromic effect observed experimentally (see below).

Based on the results of the computational investigation described above, we concluded that both the D–A–D and D–A–A′ series of BDT-containing compounds presented appropriate photophysical properties to work as emitters in LSCs, with emission in the red-orange part of the spectrum. Even **BDT-AA**, despite its different electronic properties, was computed to have a robust emission at around 570 nm, which made it potentially able to give devices with different color and light conversion ability. For this reason, all compounds described above were synthesized, and their photophysical properties were experimentally determined.

### 3.3 Synthesis

The synthesis of the new compounds started with the preparation of the oxidized central BDT core (**1**), which was carried out starting from commercially available benzo[1,2-*b*:4,5-*b*′]dithiophene-4,8-dione according to some previously reported procedures (Scheme S1, see Supporting Information for experimental details).

**Scheme 1:**
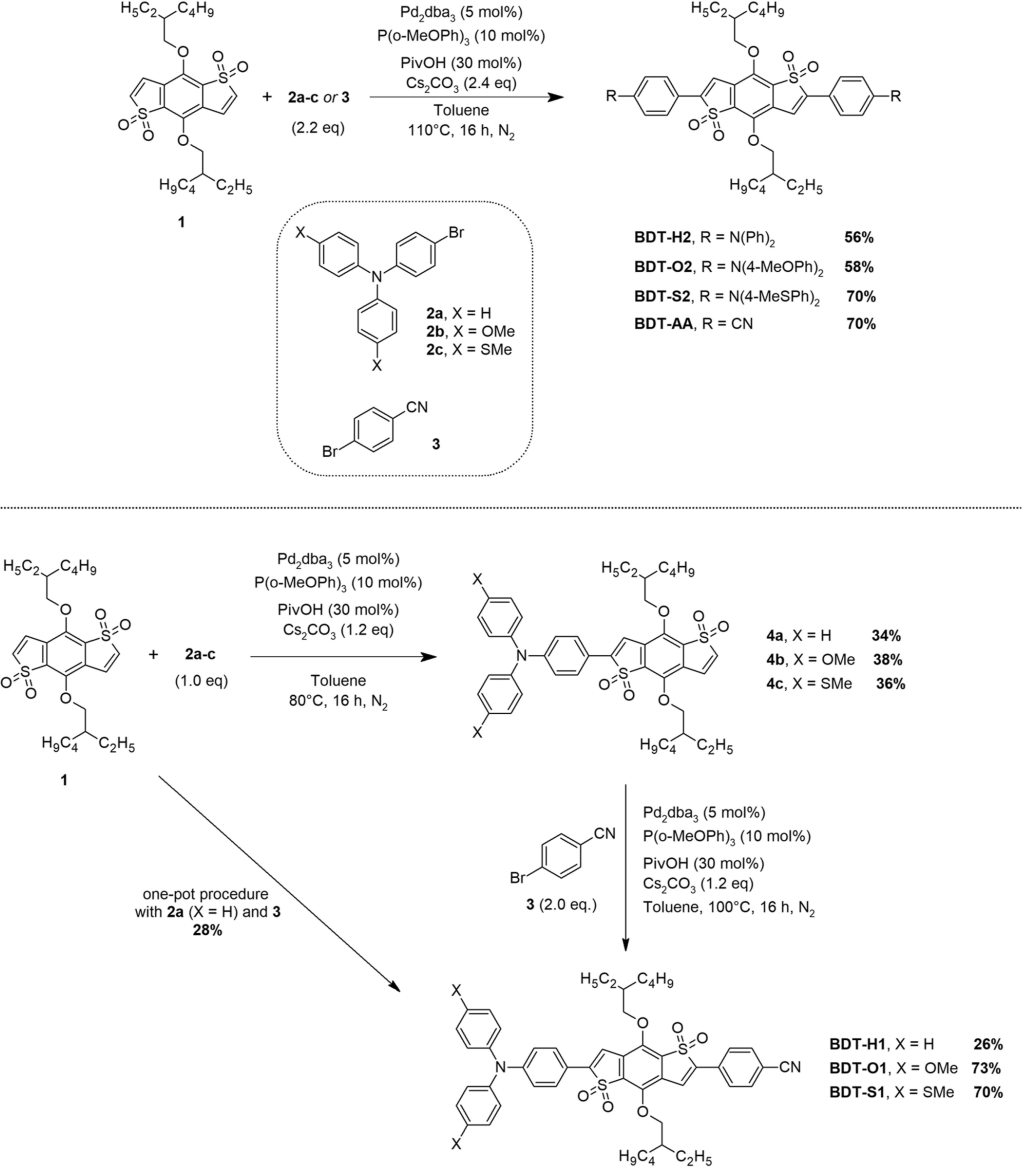
Synthesis of the New Symmetric and Non-symmetric Emitters

Since compound **1** is endowed with two oxidized thiophene rings with free 2-positions (Scheme 1), its further functionalization could be carried out by means of direct arylation procedures (differently from the original synthesis of **TPA-BDTO**, which was achieved by Suzuki–Miyaura cross-couplings).^18^ Indeed, direct arylation protocols were already shown to be effective on this class of heterocycles^54,55^ and have been employed several times by our research group to prepare highly conjugated photoactive compounds,^56−58^ allowing to shorten the synthetic sequences and reduce the amount of waste produced compared to traditional cross-coupling procedures.^59^

Considering the symmetric compounds first (Scheme 1, top), central core **1** was thus simply reacted with an excess of the required bromides **2a–c** or **3** in the presence of [Pd_2_(dba)_3_] as the catalyst precursor, P(2-MeOPh)_3_ as the ligand, PivOH as the acid additive, and Cs_2_CO_3_ as the base in refluxing toluene for 16 h. The desired compounds of the **BDT** series were obtained in 56–70% yield, with the MeS-substituted **BDT-S2** and **BDT-AA** providing the best results.

In the case of the non-symmetric derivatives, the synthesis required two consecutive direct arylation steps with different bromides (Scheme 1, bottom). First, central core **1** was desymmetrized by insertion of the three different donor groups seen above. Reaction conditions were analogous to those employed in the preparation of the symmetric emitters, except for the use of a strictly stoichiometric amount of bromides and application of a lower temperature, to minimize double arylation. Then, the isolated intermediates **4a–c** were reacted with an excess of 4-bromobenzonitrile (**3**) under similar conditions to provide the final products.

Interestingly, we could demonstrate that compound **BDT-H1** could be directly prepared in a moderate yield of 28% from central core **1** by means of a one-pot process without isolation of intermediate **4a**, as already shown by us for compounds with a different heterocyclic core.^56^ During optimization of this protocol, it was found that the yield of **BDT-H1** was limited by the need to stop the first arylation step before completion, as demonstrated by the concomitant formation of a certain amount of **BDT-AA**, clearly stemming from the double arylation of unreacted starting material **1** with 4-bromobenzonitrile (**3**).

Although some of the reactions described above proceeded with only moderate yields, all symmetric and non-symmetric compounds were obtained in quantities large enough for the subsequent characterization and device fabrication stages.

### 3.4 Steady-State Spectroscopic Characterization in Solution

**Figure 4:**
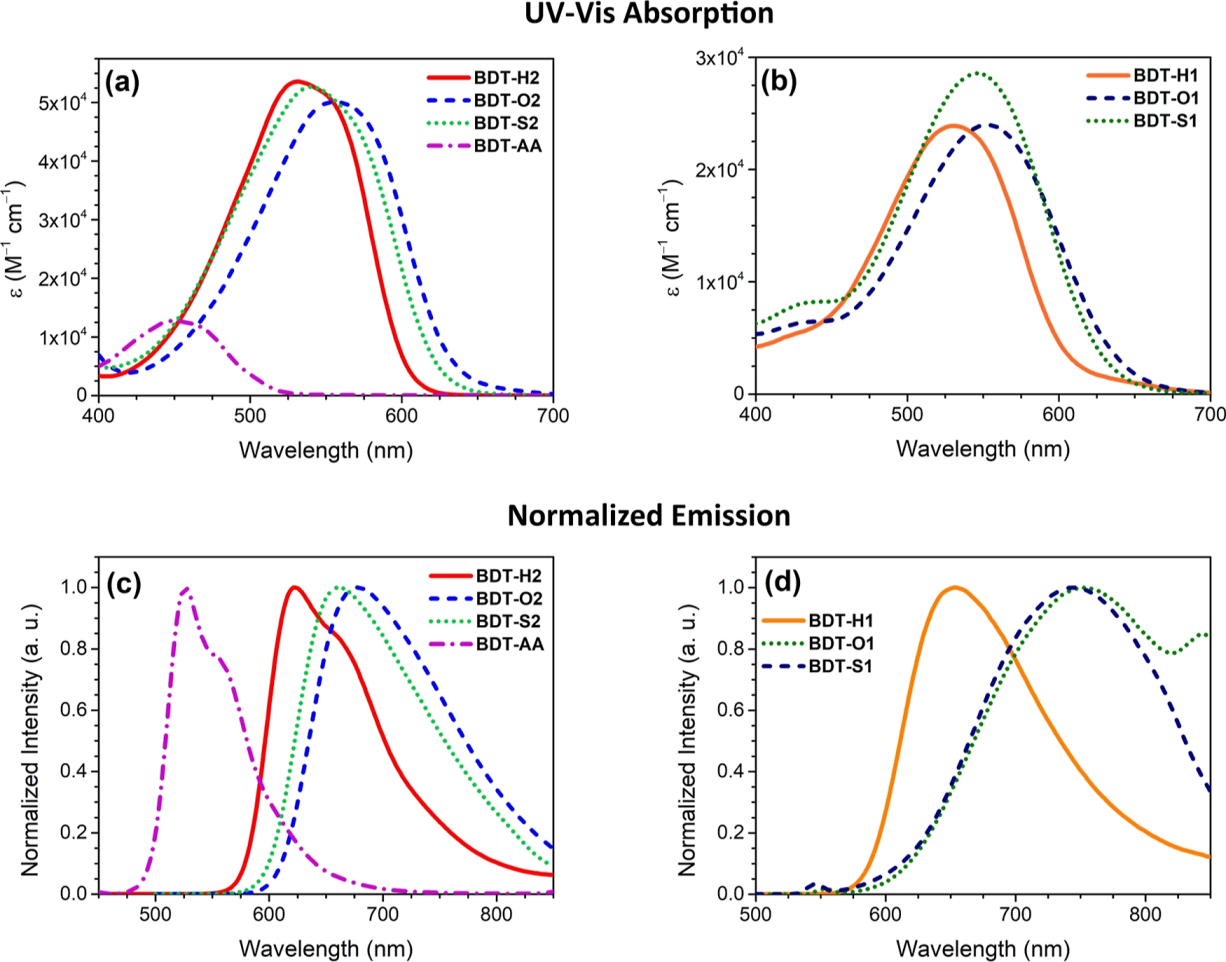
UV/vis absorption (a,b) and normalized fluorescence emission (c,d) spectra in toluene solution of the compounds prepared in this work.

The characterization of the new emitters started by recording the corresponding UV/vis absorption spectra in toluene solution (Table 3). This solvent was chosen because its refractive index (1.496) is similar to that of PMMA (1.491), which is the most commonly used polymer in LSCs, thus providing a relevant reference for the prospected application. Symmetric D–A–D compounds (Figure 4a) exhibited relatively broad absorption features, peaked above 500 nm, which, in agreement with the results of the computational investigation (see above), were attributed to the ICT transition from the donor moiety to the acceptor core. In particular, while **BDT-H2** presented a maximum absorption wavelength (λ_max_^abs^) at 530 nm, substituted analogues displayed 11–25 nm red-shifted maxima and absorption onsets in the order of donor group strength (**BDT-O2** > **BDT-S2**), with very similar molar attenuation coefficients around 5 × 10^4^ M^–1^ cm^–1^.

**Table 3:** Spectroscopic Properties of BDT-Series Compounds in Toluene Solution

compound	ε × 10^4^ [M^–^^1^ cm^–^^1^]	λ_max_^abs^ [nm]	λ_max_^emi^ [nm]	Φ_f_ [%]^b^	SS [nm] {eV}^c^
**BDT-H2**	5.36	530^a^	622^a^	77^a^	92 {0.35}
**BDT-O2**	5.01	555	678	31	123 {0.41}
**BDT-S2**	5.27	541	661	38	120 {0.42}
**BDT-H1**	2.39	531	654	68	123 {0.44}
**BDT-O1**	2.40	553	754^d^	2	201 {0.60}^d^
**BDT-S1**	2.85	546	744^d^	3	198 {0.60}^d^
**BDT-AA**	1.34	449	526	66	77 {0.40}

a In good agreement with the literature data.^18^

b Absolute QY determined using an integrating sphere, see the Experimental Section for details.

c Stokes shifts.

d For these compounds, characterized by low fluorescence QY and longer emission wavelengths, the λ_max_^emi^ and Stokes shift values are likely overestimated as a result of the application of the correction function necessary to compensate the sensitivity loss of the photomultiplier in the red wavelength region.

Symmetric compound **BDT-AA** presented a different absorption profile, with a maximum at 449 nm and a lower light harvesting ability, potentially related to its different photoexcitation process, as suggested above. As far as non-symmetric analogues are concerned (Figure 4b), the absorption properties were almost identical to those of the symmetric compounds, the only noticeable difference being a generalized reduction of molar attenuation coefficients, not exceeding 3 × 10^4^ M^–1^ cm^–1^. In general, the experimental findings were in reasonable agreement with the computed data, with differences in terms of *E*_exc_ smaller than 0.2 eV, and the same λ_max_^abs^ trend was observed within the two series of compounds (**H** < **S** < **O**).

Regarding emission spectra, parent symmetric compound **BDT-H2** presented a strong emission with maximum wavelength (λ_max_^emi^) well above 600 nm and high FQY (Φ_f_) of 77%, in good agreement with the literature data (Figure 4c).^18^ The presence of a shoulder at longer wavelength suggested the presence of different vibronic transitions. Compounds **BDT-O2** and **BDT-S2** gave rise to red-shifted emissions compared to **BDT-H2**, with λ_max_^emi^ differences of 56 and 39 nm, respectively, therefore larger than those found in the corresponding absorption spectra; moreover, the Φ_f_ was much smaller, being comprised in the 31–38% range. These observations are once again consistent with the attainment of a CT excited state upon light absorption. Indeed, in the presence of a very strong acceptor group such as BDT, the increased electron-donor capacity of the lateral groups of **BDT-S2** and, especially, **BDT-O2** compared to **BDT-H2** could further emphasize the degree of ICT of the S_0_ → S_1_ transition, with the consequent need of an extended structural reorganization of the excited state. The resulting energy loss due to vibrational relaxation could lead to the observed red shift and decrease of the quantum yield. Finally, compound **BDT-AA** presented a blue-shifted emission spectrum compared to the other symmetric species, accompanied by a good FQY of 66%.

The emission spectra of the non-symmetric derivatives presented some differences compared to those of the symmetric ones. First, compound **BDT-H1** displayed a largely red-shifted transition in comparison to **BDT-H2**, accompanied by a slightly decreased Φ_f_, probably as a result of the more pronounced CT character of the excited state imparted by the D–A–A′ architecture (Figure 4d).^37^ Due to the very similar light absorption properties of the two compounds, this resulted in a larger Stokes shift for compound **BDT-H1**, which is potentially beneficial to reduce the amount of self-absorption losses in LSC devices. Unfortunately, by changing the donor group from simple triphenylamine to the methoxy- and thiomethyl-substituted analogues, the same tendency toward a reduction of FQY observed above for the symmetric compounds was evidenced. In this case, however, it was much more pronounced, resulting in an almost complete emission quenching for compounds **BDT-O1** and **BDT-S1** in toluene solution (Table 3). It must be pointed out that, due to their weak emission, the λ_max_^emi^ values determined for these compounds are likely overestimated as a result of spectral broadening induced by the application of the correction function necessary to compensate for the sensitivity loss of the photomultiplier at long wavelengths. This is indirectly demonstrated by the large discrepancy observed between the experimental data and the computed emission maxima of **BDT-O1,S1** (see Table 2), while a much better agreement, with differences in transition energies <0.2 eV, was found in all other cases.

To further probe the photophysical properties of the new compounds, the absorption and emission spectra of **BDT-H2**, **BDT-H1**, and **BDT-AA**, as typical representatives of each series of emitters, were measured in solvents of different polarities (Figure S4, Table S3). In agreement with previous reports,^38^ the absorption spectra were generally consistent in all solvents, and a trend toward a slight red shift of the absorption maxima with increasing solvent polarity could be recognized only for compounds **BDT-H2** and **BDT-H1**, albeit with some exceptions (e.g., diethyl ether), possibly due to solubility reasons. The situation for the emission spectra was clearly different: as previously noted, **BDT-H2** presented a strong red shift of the emission in more polar solvents, coupled with progressively larger Stokes shifts and decreasing FQY values, all indicative of an excited state with a significant CT character. In the case of **BDT-H1**, this behavior was even emphasized, producing an emission maximum close to 700 nm and a Stokes shift >150 nm even in diethyl ether. Regrettably, in more polar solvents such as THF or CH_2_Cl_2_, the Φ_f_ values swiftly fell below 5%, causing the same issues seen above with the emission spectra of **BDT-O1**/**S1** in toluene. Finally, in the case of **BDT-AA**, no clear shift of the spectra with solvent polarity was observed, confirming the localized nature of the electronic transitions undergone by this compound.

Based on the results of the photophysical characterization in solution, all compounds presenting moderate to good FQY in toluene, namely, all symmetric derivatives (**BDT-H2**/**O2**/**S2**/**AA**) plus **BDT-H1**, were employed for the preparation of emissive PMMA films.

### 3.5 TAS Studies

Before starting the studies on the light-concentrating devices, we carried out some TAS experiments with sub-picosecond time resolution on selected compounds, in order to investigate the relaxation dynamics of their excited states in solution and, possibly, to rationalize the different emission quantum yields observed for some of them as a function of solvent polarity (see above). The measurements were carried out both in toluene and CH_2_Cl_2_ solution exciting the samples at 500 nm, with the exception of **BDT-AA**, whose excitation took place at 400 nm. To extract the kinetic constants describing the excited-state evolution of the samples, global analysis was used to interpret the data, employing a unidirectional linear decay scheme with three kinetic components (four in the case of **BDT-AA**). The analysis allows us to obtain both the excited-state lifetimes and the EADS, associated to each kinetic constant (see the Experimental Section for details).

**Figure 5:**
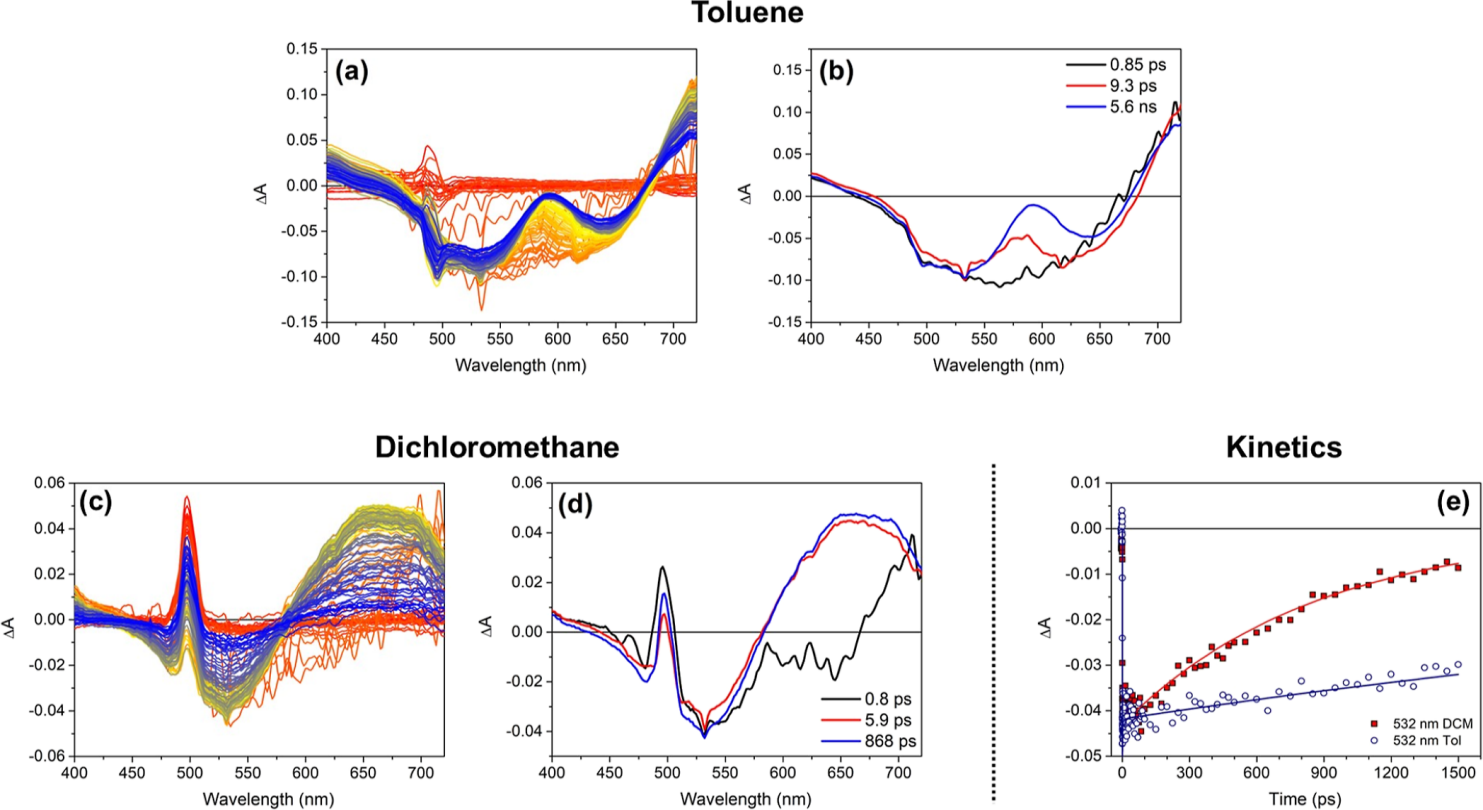
(a,c) Transient absorption spectra registered for compound **BDT-H1** in toluene and CH_2_Cl_2_, respectively [the peak at 500 nm in panel (c) is due to scattered pump light]; (b,d) EADS obtained from global analysis of the transient spectra reported in panel (a,c), respectively; and (e) kinetic traces at 532 nm registered for molecule **BDT-H1** in toluene (hollow circles) and CH_2_Cl_2_ (red squares). The continuous lines represent the fittings obtained by global analysis.

We begin our discussion with compound **BDT-H1** since it did show the largest differences in λ_max_^emi^ and Φ_f_ values in going from non-polar to polar solvents (Table S3). Its transient spectra in toluene and CH_2_Cl_2_ are reported in Figure 5, together with the EADS retrieved from global analysis.

In toluene, the transient spectrum of molecule **BDT-H1** presents two negative bands peaked at 540 and 630 nm (Figure 5a), which, by comparison with its steady-state absorption and emission spectra (Figure 4), can be assigned to ground-state bleaching (GSB) and stimulated emission (SE), respectively. Two excited-state absorption (ESA) bands can also be noted, one in the blue region of the investigated spectral interval (<470 nm) and the other peaked at about 750 nm. The comparison of the spectra measured for **BDT-H1** with those of compounds **BDT-H2** and **BDT-AA** (see below) allows us to assign the red-shifted ESA band to the presence of the nitrile-substituted benzene ring: this band is indeed observed in the case of **BDT-AA** but missing for **BDT-H2**.

The initial spectral component reported in Figure 5b, with a lifetime of 0.85 ps (black line), represents the transient spectrum of the system immediately after excitation. It presents a very broad negative signal, ascribed to the convolution of GSB and SE of the unrelaxed S_1_ excited state. The system rapidly evolves toward the second spectral component (red line). Here, we notice a red shift of the SE band, whose minimum moves toward 625 nm. This spectral evolution is interpreted in terms of a rapid stabilization of the excited state, whose electronic distribution evolves from that of the initially excited Frank–Condon state toward the minimum of the potential energy surface of the S_1_ state. From there, a further evolution is observed to occur in 9.3 ps, characterized by an additional red shift of the SE band (blue line). This further evolution can be interpreted in terms of a stabilization of the excited state associated to the solvent reorganization and to vibrational cooling. The lifetime of this final spectral component is longer than the investigated time range (>1.5 ns), which is consistent with the good FQY of **BDT-H1** in toluene.

The measurements were then repeated with **BDT-H1** dissolved in CH_2_Cl_2_, in which a significant decrease of FQY was observed (see above). The transient spectra registered in that solvent (Figure 5c) appear quite different from those measured in toluene. In this case, the SE band is not observed since an intense ESA band is present in the region between 550 and 750 nm. On the contrary, an intense bleaching band peaked at ca. 540 nm is observed also in this case, as well as a weak ESA band around 400 nm. Concerning the excited-state evolution, the initial EADS (Figure 5d, black line) somehow recalls that observed in toluene since it presents an intense bleaching band, a very weak SE band peaked at about 630 nm, and a further ESA band in the red spectral region. This EADS rapidly evolves in about 0.8 ps toward the second spectral component (red line). Here, we observe the appearance of an intense ESA peaked at about 650 nm, which completely compensates the emission band. Such a notable spectral evolution indicates that the system moves toward an excited state different from that reached immediately after light absorption. By combining this and all the previous observations, it can be inferred that the excited state reached in about 0.8 ps has a very strong CT character, with the electron density being mostly relocated on the acceptor groups present in the molecule. The following evolution (from red to blue line), occurring in 5.9 ps, can be interpreted in terms of relaxation and stabilization of such a CT state, causing a significant energy loss. In agreement with the observed evolution, the excited-state lifetime is clearly reduced in CH_2_Cl_2_ compared to toluene: in this case, the final spectral component (blue line) relaxes in about 870 ps. All these observations perfectly agree with the strong reduction of the FQY measured in CH_2_Cl_2_ as compared to toluene. The difference in the excited-state lifetimes of **BDT-H1** in the two solvents can also be noticed by comparing the kinetic traces measured on the maximum of the bleaching band, as reported in Figure 5e.

**Figure 6:**
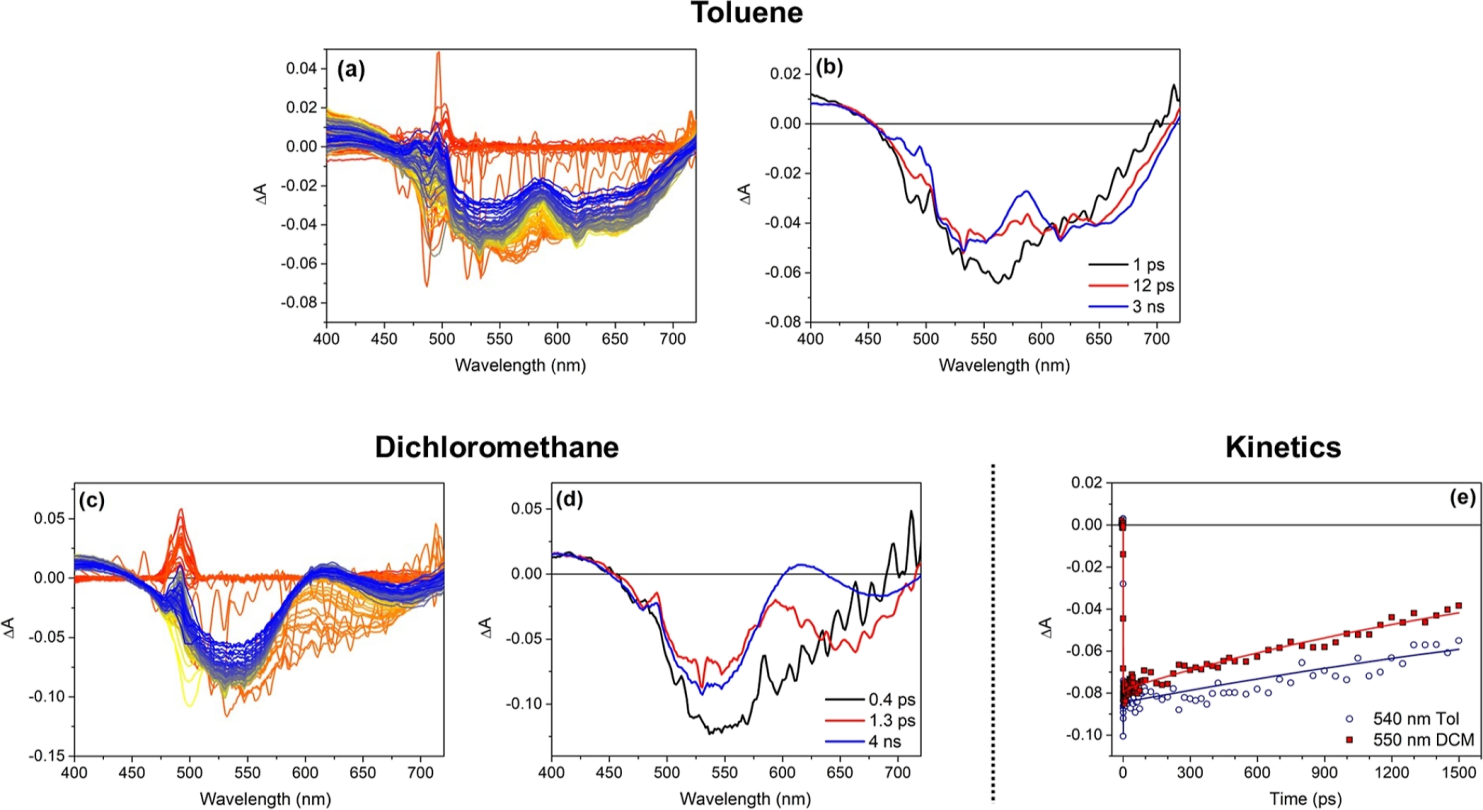
(a,c) Transient absorption spectra registered for compound **BDT-H2** in toluene and CH_2_Cl_2_, respectively [the peak at 500 nm in panel (c) is due to scattered pump light]; (b,d) EADS obtained from global analysis of the transient spectra reported in panel (a,c), respectively; and (e) kinetic traces registered on the bleaching bands of compound **BDT-H2** in toluene (hollow circles) and CH_2_Cl_2_ (red squares). The continuous lines represent the fittings obtained by global analysis.

We now turn our attention to the measurements conducted on compound **BDT-H2**. Indeed, while the appearance of the transient spectra of **BDT-H2** in toluene is similar to those of **BDT-H1**, some significant differences emerge in CH_2_Cl_2_ (Figure 6a,c) as it can also be noted by comparing the EADS obtained in the two solvents (Figure 6b,d). In toluene (Figure 6a,b), besides the GSB (ca. 530 nm) and ESA band (<470 nm), a SE band is clearly visible also for **BDT-H2** at around 625 nm, and the evolution between the different spectral components takes place on a timescale similar to what previously observed for **BDT-H1** in the same solvent (Figure 5b), including the survival of the last spectral component beyond the investigated time range. In CH_2_Cl_2_ (Figure 6c,d), contrarious to the case of **BDT-H1** (Figure 5c,d), the SE band is still visible, but it does indeed recover much faster compared to toluene because of the rise of an ESA band peaked in the same spectral region, on the picosecond timescale. Nevertheless, the excited-state lifetime in the two solvents is more similar in this case as compared to **BDT-H1**, as can be noticed by comparing the kinetic traces registered on the maximum of the corresponding bleaching signals (Figure 6e).

Clearly, also in the case of **BDT-H2**, this behavior can be explained by invoking, after the initial light absorption, the attainment of a low-lying CT excited state which gets more stabilized in the more polar solvent: however, for **BDT-H2**, the decay of this excited state seems to be much slower than for **BDT-H1**, probably due to its symmetric structure devoid of additional electron-withdrawing groups, that reduces the strength of its donor–acceptor character. This is in good agreement with its superior QY values in polar solvents.

As expected, in the case of **BDT-AA**, the same analysis gave rise to a different result since both the transient spectra, the EADS and the excited-state lifetimes, were found to be very similar in the two investigated solvents (Figure S5, where the GSB at ca. 450 nm and the SE at ca. 500–550 nm are clearly visible in both panels). This was in agreement with the less pronounced CT character of the excited state of **BDT-AA** and the relatively invariable QY values observed in different media. Finally, the same analysis was also conducted on compounds **BDT-O2**/**S2**, giving results qualitatively analogous to those obtained with **BDT-H1**/**H2** (Figures S6 and S7). Nevertheless, it must be pointed out that for these compounds in CH_2_Cl_2_ solution, the CT excited state was found to decay with much faster kinetics compared to the previous cases, and no sign of a SE band could be spotted after the initial state evolution, in agreement with the very pronounced donor–acceptor character of these compounds conferred by their strong electron-donating groups. The decay time constants obtained for all molecules in the two solvents are summarized in Table S4.

### 3.6 Emitters Characterization in the PMMA Matrix

Fluorophore-containing thin films were prepared employing PMMA, a widely used matrix in LSC systems.^60,61^ PMMA is 100% amorphous, transparent, cheap, and commercially available, making this polymer a perfect candidate for large-scale LSC applications.^62^ PMMA films with a thickness of 25 ± 5 μm were obtained by drop-casting a solution of the polymer in CHCl_3_, varying the amount of dye between approximately 0.4 and 2.8% as a percentage of the total weight of the film (see the Experimental Section for details).

**Figure 7:**
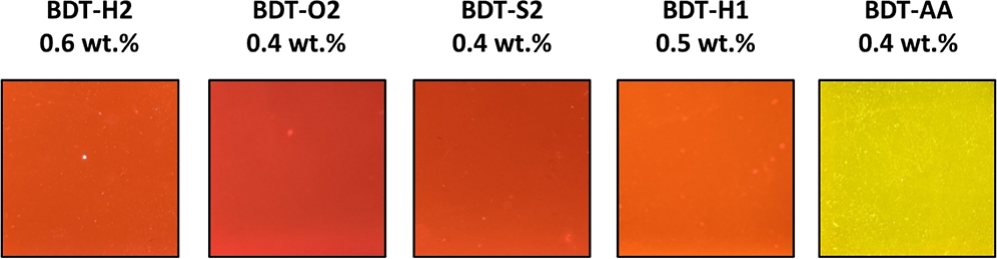
Appearance of PMMA films (portion of 2 × 2 cm^2^) doped with BDT fluorophores at 0.4–0.6 wt % concentration upon excitation in a transilluminator with a 450 nm light source.

In agreement with the optical properties in solution, all films appeared grayish to purple when illuminated with natural light, while their emission ranged from yellow to dark orange when put in a transilluminator equipped with a 450 nm light source (Figure 7). In general, the molecules showed good dispersion in the films, which appeared smooth and did not present macroscopic phase separation. Nevertheless, some significant differences emerged when analyzing their microscopic structures, as it will be highlighted below.

**Figure 8:**
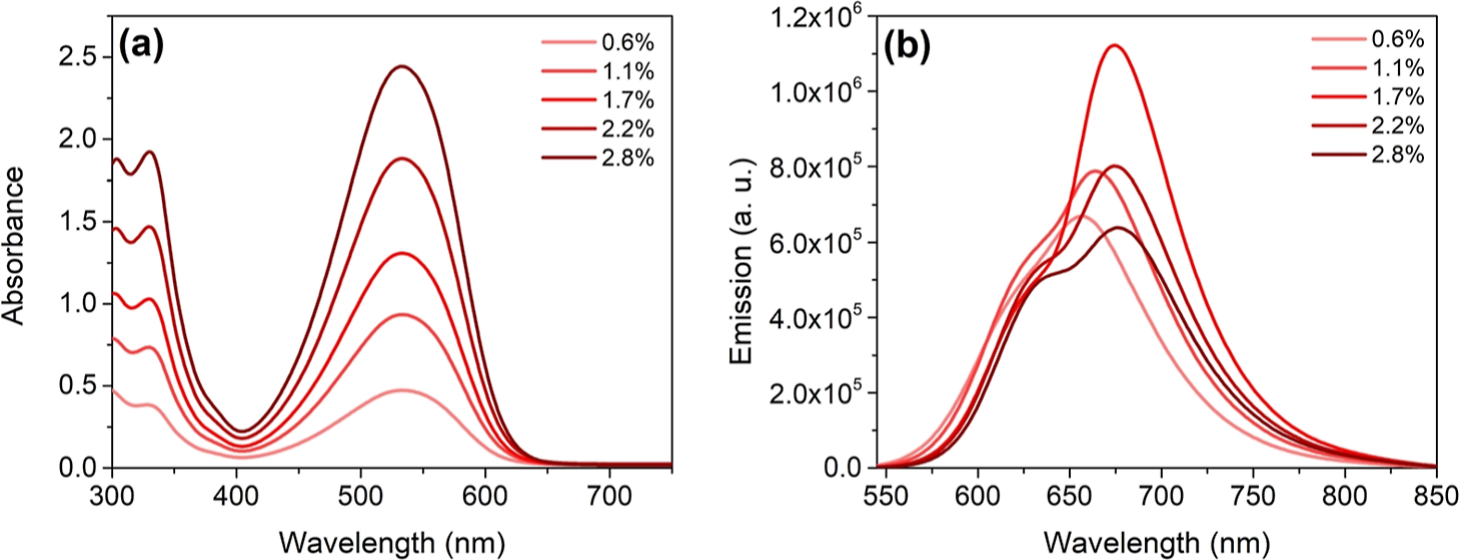
UV/vis absorption (a) and fluorescence emission (b) spectra of PMMA films containing compound **BDT-H2** at different concentrations.

The films were characterized by means of UV/vis absorption and fluorescence emission spectroscopy. Their relevant properties at the lowest concentrations are reported in Table 4. In general, absorption and emission maxima were consistent with the values obtained in toluene, resulting in significant Stokes shifts close or superior to 100 nm in all cases. On the other hand, differences in the behavior of the single emitters were observed depending on their concentration in the films, and thus they will be discussed individually. As a representative example, the absorption and emission spectra of **BDT-H2**-containing films are reported in Figure 8, while data for the other compounds are presented in the Supporting Information (Figure S8).

**Table 4:** Spectroscopic Properties of **BDT** Compounds in the PMMA Matrix at the Lowest Concentrations

compound	conc [wt %]	λ_max_^abs^ [nm]	λ_max_^emi^ [nm]	SS [nm] {eV}^a^
**BDT-H2**	0.6	533	656	123 {0.44}
**BDT-O2**	0.4	560	651	91 {0.31}
**BDT-S2**	0.4	542	633	91 {0.33}
**BDT-H1**	0.5	523	618	95 {0.36}
**BDT-AA**	0.4	447	550	103 {0.52}

a Stokes shifts.

As can be seen from Figure 8, the absorption intensity of **BDT-H2**-containing films regularly increased with the concentration, without any change of the spectral shape, denoting a good dispersion in the polymer. Regarding fluorescence, the intensity increased up to 1.7 wt %, then a partial quenching was observed for higher concentrations, accompanied by a slight red shift of the main peak; furthermore, the presence of a shoulder peak at higher energy could be spotted. This behavior is consistent both with the insurgence of re-absorption phenomena due to the increased fluorophore concentration (inner filter effect)^63^ and the possible formation of microscopic aggregates^64^ emitting at a shorter wavelength. The latter phenomenon was indeed confirmed by inspection of the corresponding epifluorescence microscopy images (Figure S9a), revealing the presence of a few fluorophore aggregates with sizes in the 30–40 μm range. Despite that, no significant decrease in the efficiency of the corresponding LSC devices was later observed (see below).

Interestingly, symmetric D–A–D compounds **BDT-O2** and **BDT-S2** and non-symmetric **BDT-H1** presented a qualitatively similar behavior to that of **BDT-H2**, albeit with some significant differences. First of all, no formation of microscopic aggregates was observed in the PMMA films of **BDT-O2**,**S2** (Figure S9b–d), possibly due to the substituents present on the 4-positions of the benzene rings of their donor groups, potentially altering their geometry in the solid state and hindering intermolecular interactions. Moreover, emissions of all these compounds appeared much weaker than that of **BDT-H2**, especially for **BDT-O2**, whose fluorescence intensity was even found to decrease progressively with fluorophore doping (Figure S8).

On the other hand, the properties of **BDT-AA**-containing films were quite different from those of the other samples: already at the minimum concentration of 0.4 wt %, the absorption spectrum displayed two shoulder peaks at the sides of the main transition at approx. 447 nm, which became very evident above 1.2 wt % (Figure S8). Based on the results of the epifluorescence microscopy experiments, this was attributed to the extensive formation of microscopic aggregates, resulting in an almost complete phase segregation between the fluorophore and the polymer matrix (Figure S9e). Accordingly, a dramatic fall in emission intensity was observed at concentrations higher than 0.4 wt %, clearly due to significant light scattering by the microcrystalline aggregates.

**Figure 9:**
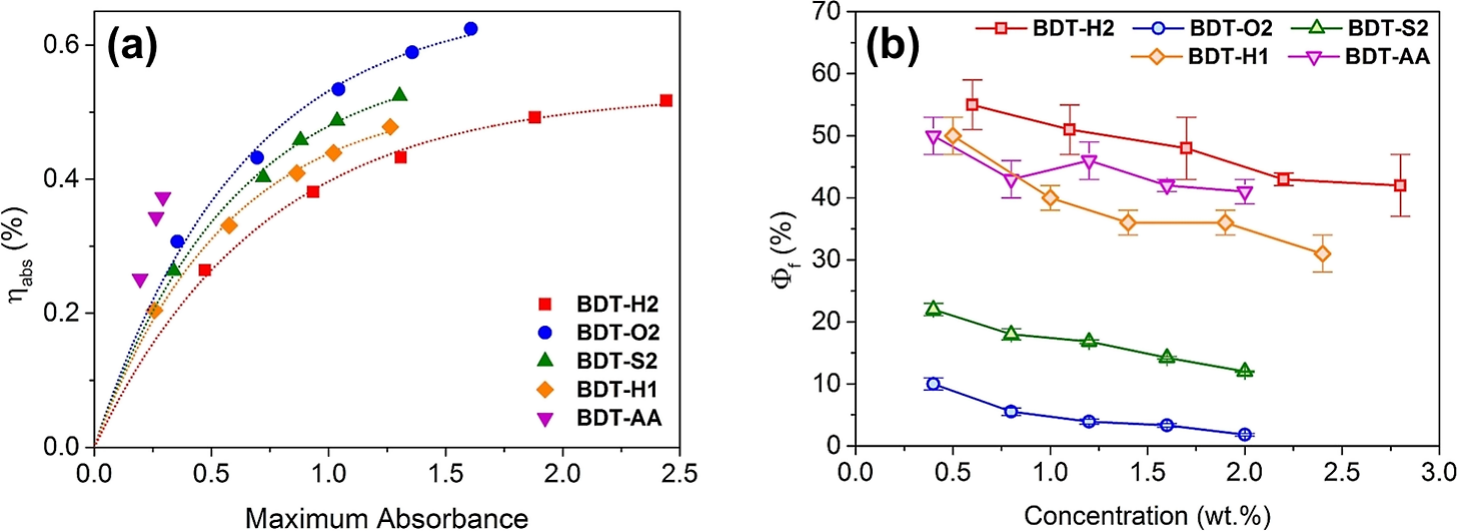
(a) Absorption efficiency in the 300–800 nm range and (b) FQYs of BDT compounds in the PMMA matrix. **BDT-H2**, red squares; **BDT-O2**, blue circles; **BDT-S2**, green upside triangles; **BDT-H1**, orange rhombs; and **BDT-AA**, purple downside triangles. In panel (a), fitting according to an exponential function is shown as a dotted line, see the Supporting Information for details.

To provide a quantitative assessment of the matching between the fluorophores absorption spectra and the emission of the solar simulator lamp used for LSC characterization (see below), the absorption efficiency parameter (η_abs_) in the 300–800 nm range was calculated for all films at each concentration, according to the definition given by Debije et al. (Figure 9a, see Supporting Information for details).^35^

All donor–acceptor compounds gave excellent η_abs_ values close to or higher than 0.5 at their maximum concentration (a value of 1 indicating a perfect match), following an exponential trend vs maximum absorbance, as previously reported.^65^ The best result was given by **BDT-O2**, due to its red-shifted and broader absorption band compared to the other compounds. **BDT-H2**, on the other hand, gave slightly lower η_abs_ than the other emitters at the same maximum absorbance level, but the very high absorbance values of its films ensured an optimal light harvesting ability of the corresponding devices. As for **BDT-AA**, apparently, it gave high η_abs_ values at low maximum absorbance, but the analysis in this case was surely affected by the abovementioned aggregation phenomena, which caused excessive tailing of the spectrum at longer wavelengths due to scattering (Figure S8), leading to an overestimation of η_abs_.

Finally, FQYs were measured for all fluorophore-doped films using an integrating sphere (see the Experimental Section for details) and are shown in Figure 9b. In agreement with the measurements in toluene solution, the highest Φ_f_ values were shown by compound **BDT-H2**, with a maximum of 55% at 0.6 wt %, which moderately decreased up to approx. 42% at the highest concentration, likely due to inner filter effects combined with the possible formation of less emissive supramolecular (amorphous) chromophoric aggregates.^66^ Although these values were inferior to that recorded in toluene (77%), they were still compatible with the fabrication of efficient LSC devices. Slightly lower quantum yields were obtained for emitter **BDT-H1** and even **BDT-AA**, despite the abovementioned formation of micro-aggregates, denoting the excellent light emission properties of the latter compound. Unfortunately, as suggested by their emission spectra, the other symmetric D–A–D compounds presented much lower Φ_f_ values, once again in agreement with results in solution (see above). In particular, the FQYs of **BDT-O2** were always equal or inferior to 10% at all concentrations, suggesting that the corresponding LSC could present unsatisfying optical efficiencies.

### 3.7 Performances of LSC Devices

**Figure 10:**
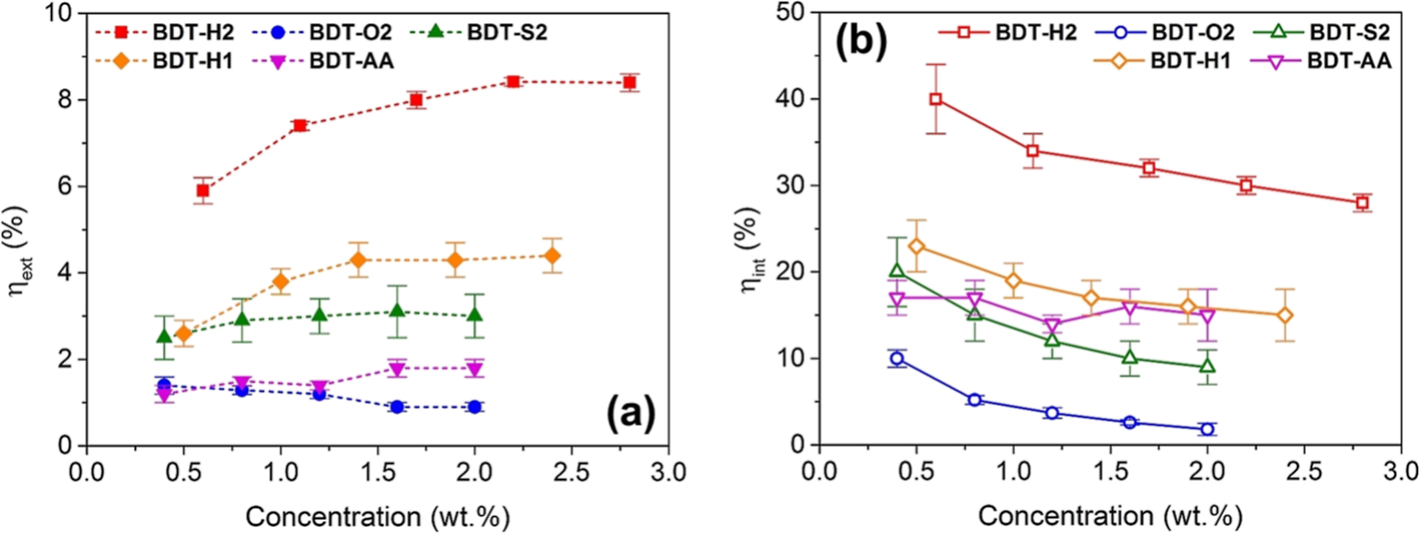
External (a, full symbols) and internal (b, hollow symbols) photon efficiencies of LSCs built with BDT compounds. **BDT-H2**, red squares; **BDT-O2**, blue circles; **BDT-S2**, green upside triangles; **BDT-H1**, orange rhombs; and **BDT-AA**, purple downside triangles.

The performances of the fluorophore-containing polymer films as LSCs were first characterized by recording their external and internal photon efficiencies under simulated solar light (Figure 10, see Supporting Information for details).

The external photon efficiency (η_ext_) corresponds to the ratio between the output photon flux measured at the edges of the LSC with respect to the incident photon flux, and it allows us to evaluate both the light-harvesting ability of the LSC and the effectiveness of the waveguide propagation within the devices. As expected from the spectroscopic characterization, the best η_ext_ figures were obtained with compound **BDT-H2**, which reached the maximum value of approx. 8.4% for the two films with the highest fluorophore concentrations. This was probably due to an optimal balance between the excellent light-absorption ability of **BDT-H2** and the losses due to dissipative phenomena and light scattering, which are both enhanced at increasing fluorophore concentrations. This result suggests that the large Stokes shifts and small spectral overlaps of the BDT-series compounds can indeed be successfully exploited to maximize the device efficiency. The performance of **BDT-H2** in terms of η_ext_ is remarkable, appearing superior to those of other organic donor–acceptor fluorophores recently reported both by us and other research groups.^16,42,65,67−69^

The other compounds provided inferior results compared to **BDT-H2**, but the general tendency toward higher η_ext_ values at higher fluorophore concentration was confirmed, except for **BDT-O2**, for which a slight decrease was observed instead. Indeed, its overall performance was quite poor, clearly due to its very low FQY compared to the other compounds of the series. Interestingly, **BDT-H1** provided fair η_ext_ values with a maximum of around 4.4%, but was still quite far from **BDT-H2**, despite its slightly higher η_abs_ (see above). We can attribute this behavior to its much weaker light harvesting ability compared to **BDT-H2**, as evidenced by the lower absorbance values of the corresponding films (as compared in Figure 8 and Figure S8), together with its lower FQY and smaller Stokes shift in the polymer matrix (Table 4).

Then, to evaluate the photon transport process within the waveguide, the internal photon efficiency (η_int_) was determined. This parameter can be obtained from the edge-emitted power spectra, calculating the ratio between the average output power emitted from the four edges and the fraction of photons effectively absorbed by the LSC (see the Supporting Information for details). Being related to the number of absorbed photons (rather than the total incident power, as η_ext_), η_int_ is a key parameter that allows evaluating in detail all lightguide losses taking place in the device. In almost all cases, η_int_ was maximized at the lowest emitter concentration (Figure 10b) and moderately decreased as the concentration increased. This is a typical trend often observed for LSCs and is usually consistent with that observed for the Φ_f_ values, suggesting that a progressively larger fluorescence dissipation occurred within the waveguide. Interestingly, in this case, the only exception was **BDT-AA**, for which relatively constant η_int_ values were observed across the entire concentration range. It is possible that, due to the abovementioned formation of microaggregates, intense light scattering occurs even at low concentrations, resulting in smaller η_int_ values compared to **BDT-H1** even with slightly better FQYs (Figure 9), but without worsening too much with concentration, thus explaining the relatively constant η_int_ trend.

**Figure 11:**
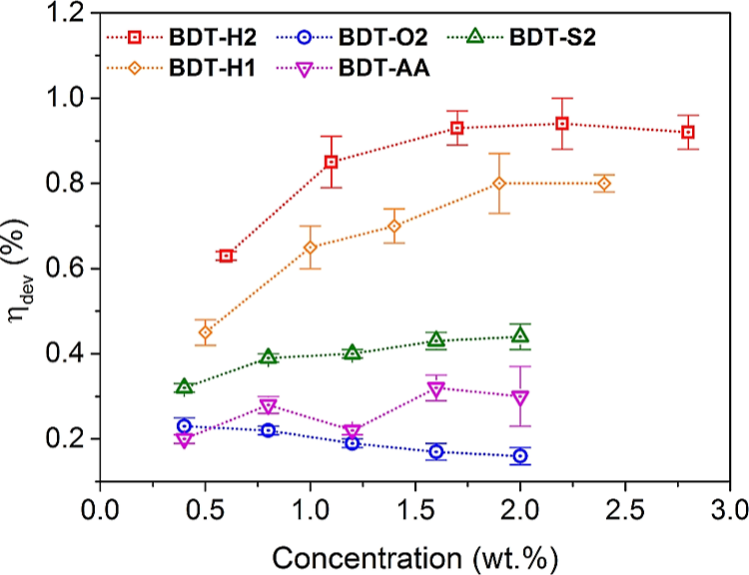
Device efficiencies of LSC built with BDT compounds. **BDT-H2**, red squares; **BDT-O2**, blue circles; **BDT-S2**, green upside triangles; **BDT-H1**, orange rhombs; and **BDT-AA**, purple downside triangles.

To assess the LSC performances in the direct light-to-electricity conversion, we determined the device efficiency (η_dev_) parameter, connecting two Si-PV cells in series to an edge of the thin-film LSC by using silicone grease and measuring the electric power generated by such device under simulated solar irradiation (Figure 11, see the Experimental Section for details).

The obtained parameter can be defined as the electrical power effectively extracted from the PV cells (*P*_el_^out^) relative to the luminous power hitting the top surface of the LSC (*P*_opt_^in^) and is conceptually analogous to the PCE of a PV cell (eq 1)
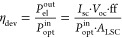
1where ff, *I*_sc_, and *V*_oc_ are the fill factor, short-circuit current, and open-circuit voltage of the edge-mounted PV cells, respectively, *A*_LSC_ is the front-illuminated area of the LSC device, and *P*_opt_^in^ is the incident solar power density expressed in mW cm^–2^.

Results were in general agreement with the trends observed for the η_ext_ parameter, with the best performance once again provided by **BDT-H2**, whose device reached a maximum value of 0.94% at 2.2 wt %. The efficiency reached by **BDT-H1** was also noteworthy, with a maximum value of 0.8% at the relatively high doping levels of 1.9–2.4 wt %. On the other hand, much lower figures could be gathered from the other compounds due to the above-described issues of low Φ_f_ values and extensive light scattering, which effectively decreased the amount of photons hitting the solar cells and, consequently, their electricity production.

The best results obtained for all compounds together with the corresponding doping concentrations are reported in Table 5, alongside those obtained with state-of-the-art commercial emitter Lumogen F Red 305 (**LR305**), which, compared to the compounds discussed in this study, presents not only a higher Φ_f_ value but also a more extensive re-absorption.^3,70^

**Table 5:** Best Efficiency Parameters of the LSC Built with BDT Compounds and **LR305** in the PMMA Matrix

compound	conc [wt %]	η_ext_ [%]	η_int_ [%]	η_dev_ [%]
**BDT-H2**	0.6		40 ± 4	
	2.2	8.4 ± 0.1		0.94 ± 0.06
**BDT-O2**	0.4	1.4 ± 0.2	10 ± 1	0.23 ± 0.02
**BDT-S2**	0.4		20 ± 4	
	1.6	3.1 ± 0.6		
	2.0			0.44 ± 0.03
**BDT-H1**	0.5		23 ± 3	
	2.4	4.4 ± 0.4		0.80 ± 0.02
**BDT-AA**	0.4		17 ± 2	
	1.6	1.8 ± 0.2		0.32 ± 0.03
**LR305**	0.4		50 ± 7	
	1.6			1.0 ± 0.1
	2.0	9.1 ± 0.4		

While the parameters extracted from the LSC built with **LR305** were still the best among those measured, it is remarkable that **BDT-H2** provided similar performances, especially in terms of η_dev_. Even in the case of **BDT-H1**, with much lower photon efficiency values, the best η_dev_ value was only approx. 20% lower than that of **LR305**. This highlights how the design of the BDT-series emitters and the low re-absorption losses associated with their large Stokes shifts can generate an emission profile highly matched with the light absorption of silicon solar cells.

Finally, we also assessed the photostability of a **BDT-H2**-containing PMMA film with an accelerated test, consisting of continuous irradiation under UV light (350–420 nm) at a constant temperature of 70 °C for 650 min (potentially corresponding to a much longer period of time at room temperature, see the Supporting Information for details). The selected film was that containing 2.2 wt % of fluorophore as it provided the best results among all those tested in terms of η_ext_ and η_dev_ (Table 5). The emission spectrum of the incident light is reported in Figure S15 together with that of the AM 1.5G solar spectrum. Its power density was integrated to be 38.43 W m^–2^ (95% peak area), corresponding to irradiation conditions of 1.15 sun, considering that in the same region the AM 1.5G spectrum presents an irradiance of 33.45 W m^–2^. Under these conditions, the film demonstrated approx. 20% loss in maximum absorption intensity during the entire experiment (Figure S16a), which was mirrored by a progressive reduction of the area of its emission profile (Figure S16b). Nevertheless, the general shape of the absorption spectrum was not significantly altered, and only a small blue shift of approx. 9 nm was observed, indicating that most of the fluorophore was not degraded in the accelerated aging test.

## 5 Conclusions

In this paper, we have described the design, synthesis, and spectroscopic characterization of a series of organic fluorescent emitters bearing an electron-withdrawing benzo[1,2-*b*:4,5-*b*′]dithiophene 1,1,5,5-tetraoxide unit as their central core. The compounds had either a symmetric (D–A–D or A′–A–A′) or non-symmetric (D–A–A′) structure, characterized by the presence of different electron-donating or electron-accepting groups at the sides of the central heterocyclic system. A DFT and TD-DFT computational investigation revealed that compounds containing both donor and acceptor groups were expected to attain excited states characterized by a large degree of ICT upon photoexcitation, potentially yielding emissions in the red part of the spectrum accompanied by large Stokes shifts.

Accordingly, the optical properties of the emitters in solution were found to be critically dependent on their general structure and on the nature of the substituents: in particular, the presence of strong electron-donating groups connected to the central BDT-tetraoxide unit caused a significant red shift of the emission, which was also observed when going from symmetric to the corresponding non-symmetric structures. Such bathochromic shifts were regrettably accompanied by a more or less pronounced decrease of FQY, which in some cases prevented the fluorophores use in LSCs (**BDT-O1,S1**). TAS studies confirmed that, after the initial photoexcitation, the compounds underwent a fast transition to lower-lying ICT excited states, whose lifetime was largely dictated both by their structures and the surrounding environment. Thus, non-symmetric compounds (**BDT-H1**), as well as compounds with stronger electron-donating groups (**BDT-O2,S2**), showed a much faster excited-state decay compared to **BDT-H2**, especially in more polar solvents, in good agreement with the corresponding Φ_f_ trends.

All emitters presenting moderate-to-good FQY in toluene solution were dispersed in a PMMA matrix at different concentrations for LSC studies. In general, LSC performances were enhanced in the case of a strong emission accompanied by an efficient minimization of self-absorption phenomena, as it is typical in this kind of devices. Thus, despite its slightly lower light absorption efficiency, **BDT-H2** yielded the most efficient concentrators, thanks to its higher Φ_f_ and smaller overlap compared to the other compounds of the series. Remarkably, both the photonic (η_ext_ of 8.4 ± 0.1%) and PV (η_dev_ of 0.94 ± 0.06%) efficiency figures of LSC built with **BDT-H2** were higher than those recently reported for several other organic emitters and were very close to those of state-of-the-art devices fabricated with standard fluorophore **LR305**.

This study thus highlights that, in the case of organic emitters for LSCs, finding the right balance between the relative strengths of the connected donor and acceptor groups, as well as the most appropriate molecular architecture, is key to achieve the best compromise between all the relevant photophysical properties (light harvesting ability, maximum absorption wavelength, FQY, and Stokes shift), leading to solar collectors with optimized performances.

## Supporting Information Available

Computational analysis; synthetic procedure for the preparation of compound **1**; copies of the ^1^H and ^13^C NMR spectra of all new compounds; additional spectroscopic characterization of the compounds in solution and in the PMMA matrix; TAS analysis; experimental details on the optical and PV efficiency measurements; and experimental details on the accelerated lifetime tests (PDF)
