# Supplementary material for: Orange/Red Benzo[1,2-b:4,5-b′]dithiophene 1,1,5,5-Tetraoxide-Based Emitters for Luminescent Solar Concentrators: Effect of Structures on Fluorescence Properties and Device Performances
Source: ACS Appl Energy Mater. 2023 Apr 20;6(9):4862–80. doi: 10.1021/acsaem.3c00362 (PMC10170478; doi:10.1021/acsaem.3c00362)
Supplement: Supplementary file 1 — ae3c00362_si_001.pdf [file ae3c00362_si_001.pdf]

Supporting Information

Orange/red Benzo[1,2-*b*:4,5-*b*′]dithiophene 1,1,5,5-tetraoxide-Based Emitters for Luminescent Solar Concentrators: Effect of Structure on Fluorescence Properties and Device Performances

Matteo Bartolini,[a,‡] Cosimo Micheletti,[b,‡] Alberto Picchi,[b,‡] Carmen Coppola,[c,d] Adalgisa Sinicropi,[a,c,d] Mariangela Di Donato,[a,e] Paolo Foggi,[e,f,h] Alessandro Mordini,[a,g] Gianna Reginato,[a] Andrea Pucci,[a,b,*] Lorenzo Zani,[a,*] Massimo Calamante[a,g]

[a] Institute of Chemistry of Organometallic Compounds (CNR-ICCOM), Via Madonna del Piano 10, 50019 Sesto Fiorentino, Italy; [b] Department of Chemistry and Industrial Chemistry, University of Pisa, Via G. Moruzzi 13, 56124 Pisa, Italy; [c] Department of Biotechnology, Chemistry and Pharmacy, R^{2}ES Lab, University of Siena, Via A. Moro 2, 53100 Siena, Italy; [d] CSGI, Consorzio per lo Sviluppo dei Sistemi a Grande Interfase, Via della Lastruccia 3, 50019 Sesto Fiorentino, Italy; [e] LENS, European Laboratory for Non-Linear Spectroscopy, Via N. Carrara 1, 50019 Sesto Fiorentino, Italy; [f] Department of Chemistry, Biology and Biotechnology, University of Perugia, Via Elce di Sotto 8, 06123 Perugia, Italy; [g] Department of Chemistry ''U. Schiff'', University of Florence, Via della Lastruccia 13, 50019 Sesto Fiorentino, Italy; [h] National Institute of Optics (CNR-INO), Via N. Carrara 1, 50019 Sesto Fiorentino, Italy.

Corresponding authors email: andrea.pucci@unipi.it; lorenzo.zani@iccom.cnr.it.

[‡] *These authors contributed equally to this work.*

Table of Contents

1. DFT and TD-DFT computational investigation........ S2
2. Synthesis of the oxidized BDT central core **1**........ S7
3. Copies of the NMR spectra of compounds **BDT-H2**, **O2**, **S2 AA**, **4a-c** and **BDT-H1**, **O1**, **S1**........ S8
4. Spectroscopic characterization in different solvents........ S28
5. Additional figures and tables for the TAS measurements........ S30
6. Characterization of fluorophore-doped PMMA films........ S34
7. Absorption efficiency calculation........ S36
8. Measurement of external (η_{ext}) and internal (η_{int}) photon efficiency........ S37
9. Measurement of device photovoltaic efficiency (η_{dev})........ S39
10. Accelerated photodegradation test........ S40
11. References........ S42

1. DFT and TD-DFT computational investigation

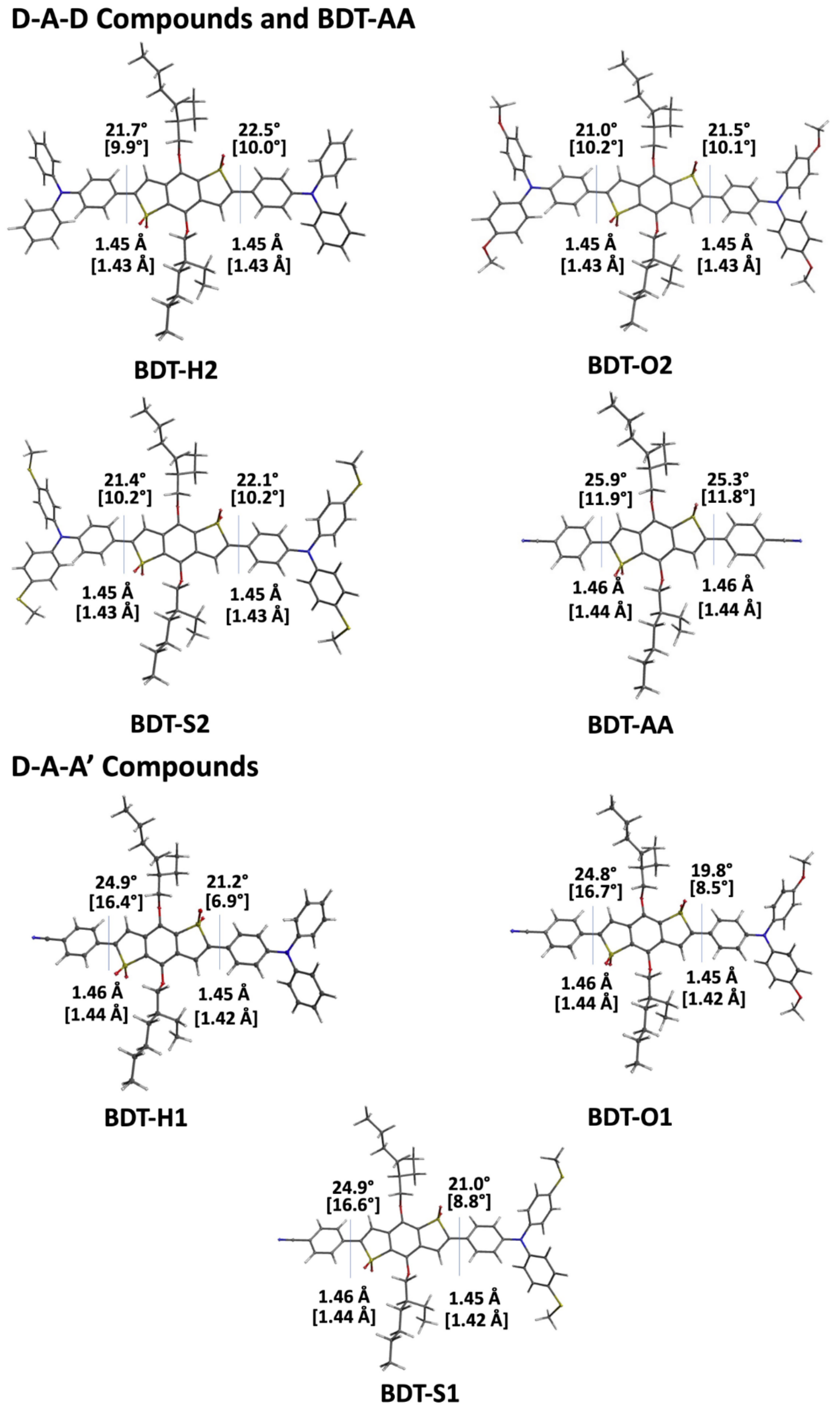

Figure S1. *Bond lengths (Å) and dihedral angles (degrees) of S_0 and S_1 (in brackets) optimized geometries of all compounds of the* ***BDT*** *series.*

Table S1. *Ground state energies (in eV) of the FMOs of all compounds of the* ***BDT*** *series computed in toluene at the B3LYP/6-31G* level.*

Compound	HOMO−1	HOMO	LUMO	LUMO+1
**BDT-H2**	−5.276	−5.072	−2.694	−1.489
**BDT-O2**	−4.991	−4.814	−2.602	−1.381
**BDT-S2**	−5.033	−4.890	−2.690	−1.486
**BDT-H1**	−6.325	−5.270	−3.010	−1.952
**BDT-O1**	−6.153	−5.022	−2.966	−1.911
**BDT-S1**	−5.986	−5.056	−3.007	−1.950
**BDT-AA**	−7.262	−6.468	−3.315	−2.294

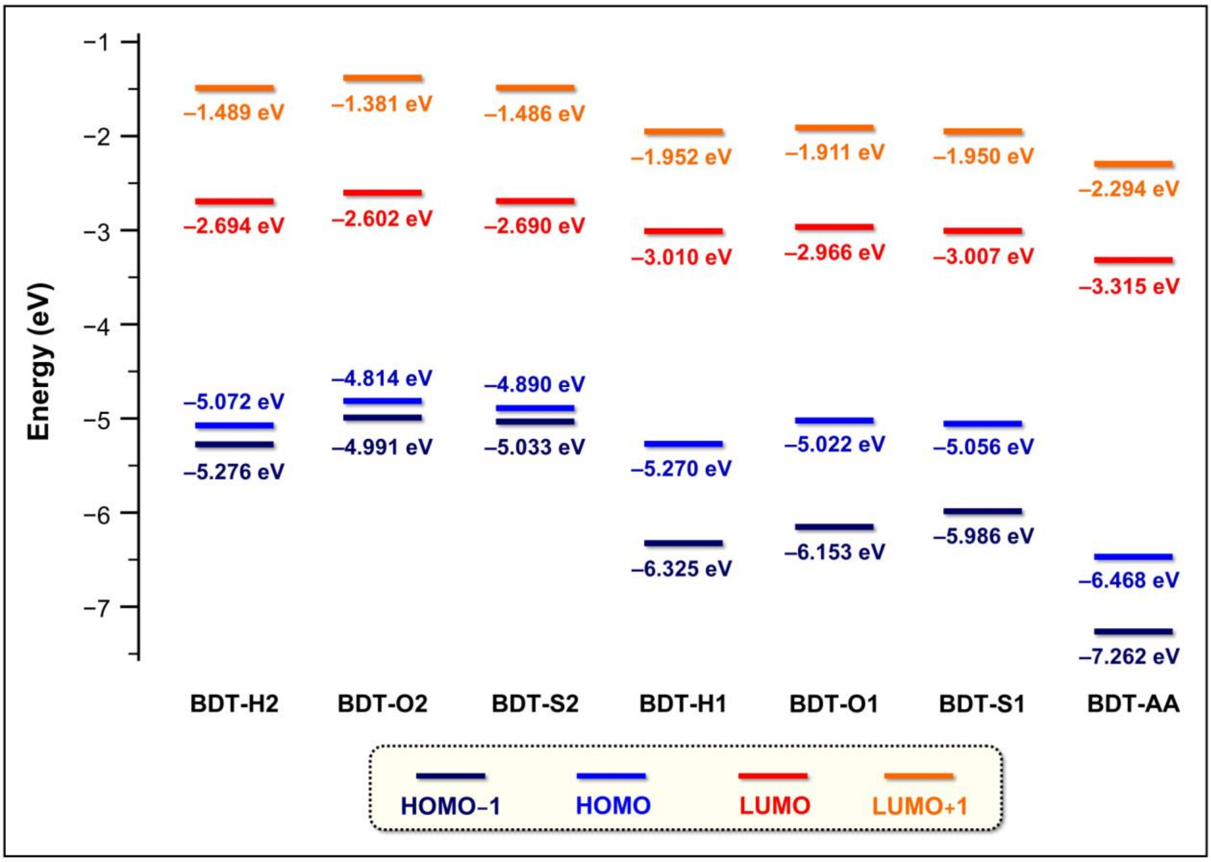

Table S2. *First excited state energies (in eV) of the FMOs of all compounds of the* ***BDT*** *series computed in toluene at the TD-CAM-B3LYP/6-31G* level.*

Compound	HOMO−1	HOMO	LUMO	LUMO+1
**BDT-H2**	−6.521	−6.062	−2.027	−0.475
**BDT-O2**	−6.207	−5.848	−1.956	−0.399
**BDT-S2**	−6.268	−5.955	−2.046	−0.502
**BDT-H1**	−7.476	−6.331	−2.301	−0.897
**BDT-O1**	−7.248	−6.077	−2.267	−0.878
**BDT-S1**	−7.205	−6.164	−2.312	−0.915
**BDT-AA**	−8.430	−7.079	−2.635	−1.106

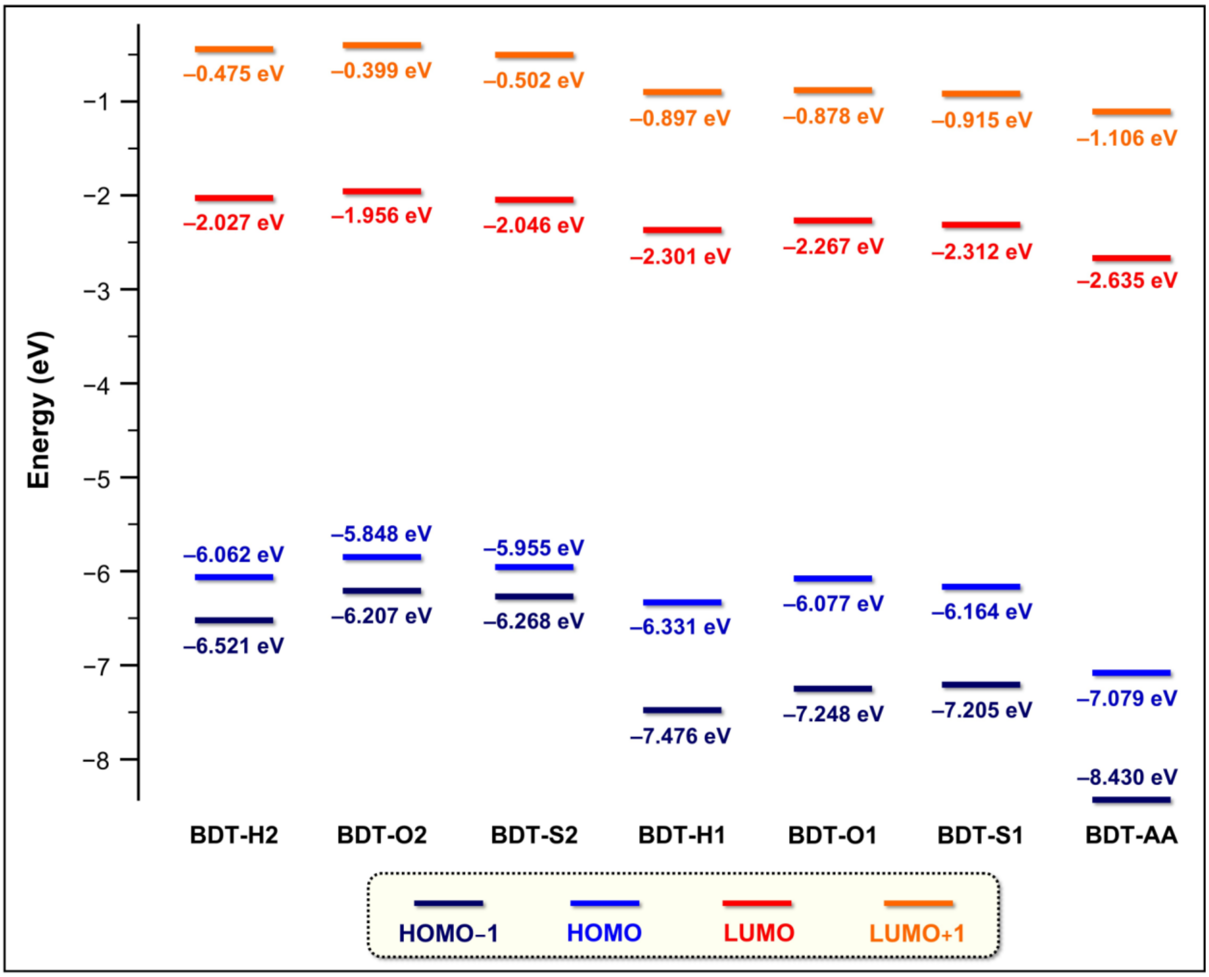

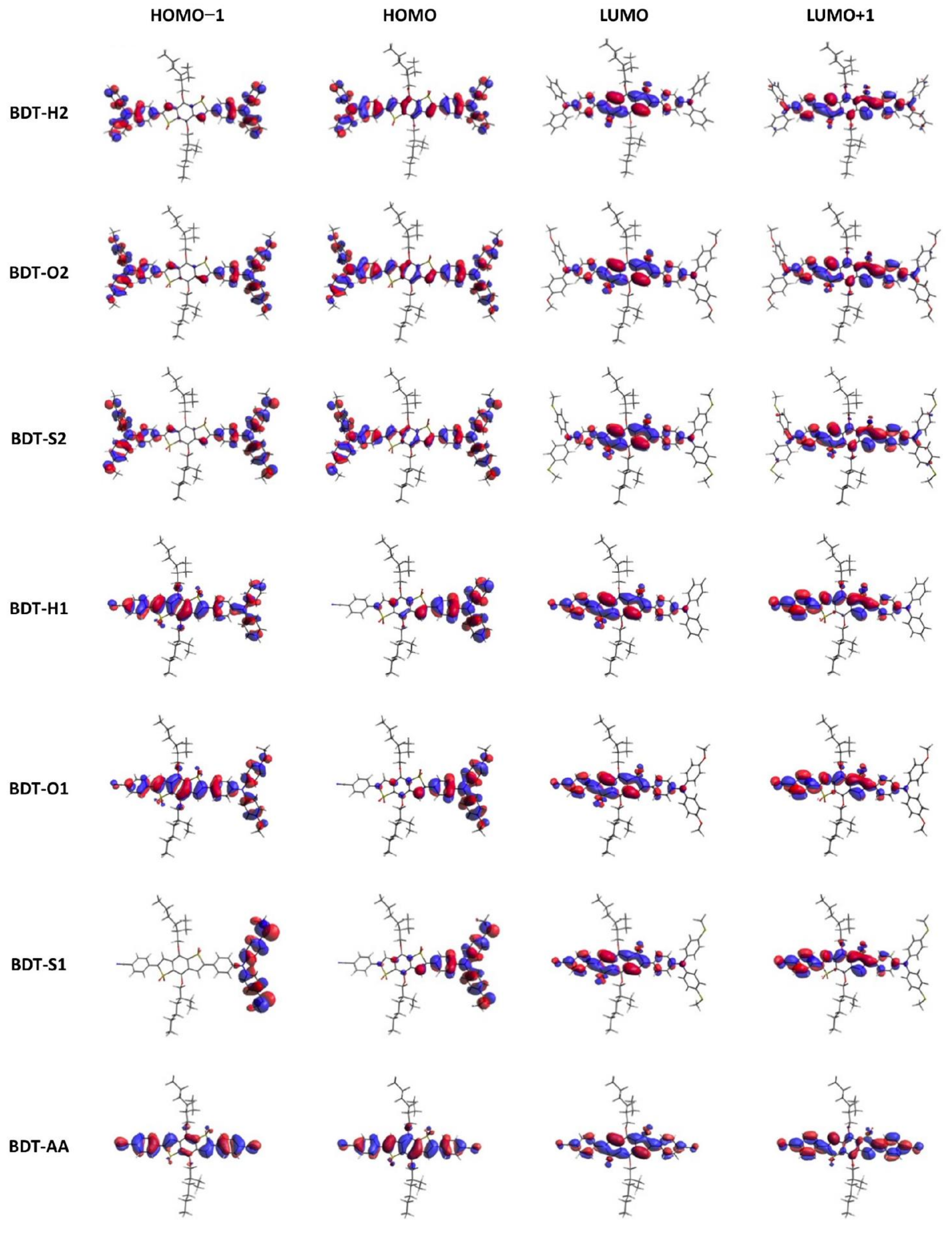

Figure S2. *DFT (B3LYP/6-31G*) ground state frontier molecular orbitals (FMOs) of* ***BDT*** *series compounds in toluene solution.*

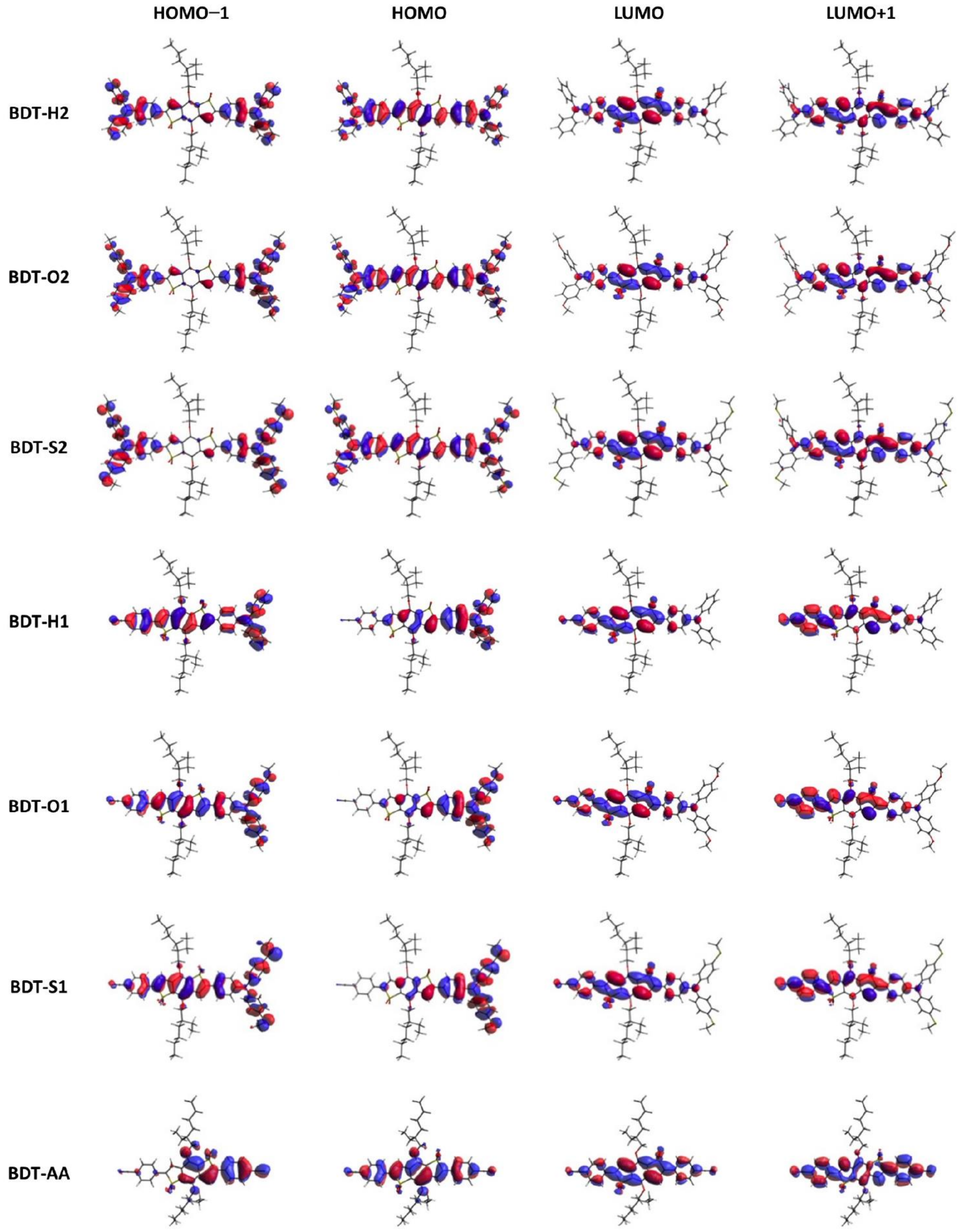

Figure S3. *TDDFT (CAM-B3LYP/6-31G*) first excited state frontier molecular orbitals (FMOs) of **BDT** series compounds in toluene solution.*

2. Synthesis of the oxidized BDT central core 1

The synthesis of the new compounds started with the preparation of the oxidized central BDT core. According to some previously reported procedures,[1-3] starting material benzo[1,2-*b*:4,5-*b*′]dithiophene-4,8-dione (**A**) was first reduced with metallic zinc and then alkylated to yield intermediate **B**. The latter was then oxidized with *m*-CPBA to give the corresponding sulfur tetraoxide derivative **1** in good yield (Scheme S1).

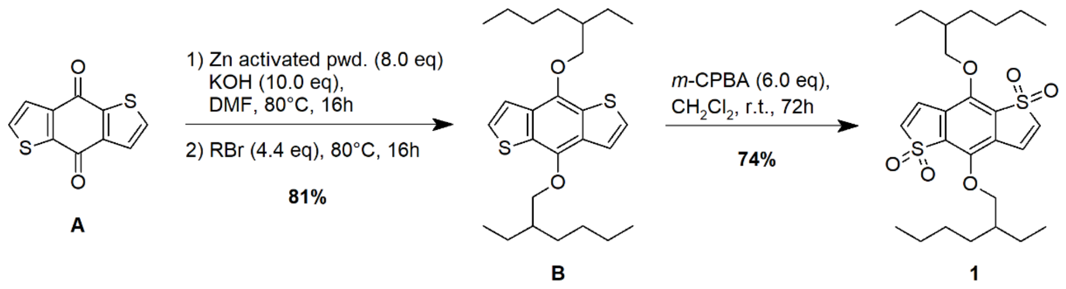

***Scheme S1.** Synthesis of the oxidized BDT central core **1**.*

4,8-*Bis*((2-ethylhexyl)oxy)benzo[1,2-*b*:4,5-*b*']dithiophene (B)

Benzo[1,2-*b*:4,5-*b*′]dithiophene-4,8-dione (**A**, 50 mg, 0.23 mmol, 1.0 eq) was dissolved in dry and degassed *N,N*-DMF (5 mL). Then, Zn powder (120 mg, 1.84 mmol, 8.0 eq, previously activated) and KOH (130 mg, 2.30 mmol, 10.0 eq) were added to the reaction mixture and the latter was heated to 80°C for 16 h under an inert atmosphere of N_2. The mixture slowly turned from dark blue to rotten green. 2-Ethylhexyl bromide (193 mg, 1.0 mmol, 4.4 eq) was then added, and the suspension was stirred for an additional 16 h. After cooling to room temperature, the mixture was filtered over a short pad of Celite© to remove the excess Zn, washed with HCl 1M (10 mL) and water (10 mL), and extracted with CH_2Cl_2 (3 × 10 mL). The combined organic layers were dried over Na_2SO_4 and concentrated under vacuum. The residue was purified by flash column chromatography (SiO_2, Petroleum Ether/ CH_2Cl_2 6:1 to 4:1) to yield product **2** (83 mg, 0.19 mmol, 81%) as a yellow oil. ^{1}H-NMR ($CDCl_3$, 200 MHz): δ = 7.49 (d, *J* = 5.5 Hz, 2 H), 7.37 (d, *J* = 5.5 Hz, 2 H), 4.19 (d, *J* = 5.2 Hz, 4 H), 1.85 – 1.33 (m, 18 H), 1.03 (t, *J* = 7.4 Hz, 6 H), 0.94 (t, *J* = 7.4 Hz, 6 H) ppm. The analytical data are in agreement with those reported in the literature.[1]

4,8-*Bis*((2-ethylhexyl)oxy)benzo[1,2-*b*:4,5-*b*']dithiophene 1,1,5,5-tetraoxide (1)

Compound **B** (60 mg, 0.13 mmol, 1.0 eq) was dissolved in CH_2Cl_2 (5 mL) and the solution was cooled to 0°C. Then, a second solution of *m*-CPBA (140 mg, 0.81 mmol, 6.0 eq) in CH_2Cl_2 (10 mL) was added dropwise to the first one. The resulting mixture was shielded from the light. The yellow solution was allowed to warm up to room temperature and stirred for 72 h. The reaction was then quenched with H_2O, extracted with CH_2Cl_2 (3 x 10 mL) and carefully washed with a solution of $NaHCO_3$ (3 x 10 mL). The combined organic layers were dried over Na_2SO_4 and concentrated under vacuum. The residue was purified by flash column chromatography (SiO_2, Petroleum Ether / CH_2Cl_2 1:1) to yield product **3** (51 mg, 0.1 mmol, 74%) as a pale-yellow solid. ^{1}H-NMR ($CDCl_3$, 200 MHz): δ = 7.39 (d, *J* = 7.0 Hz, 2 H), 6.70 (d, *J* = 7.0 Hz, 2 H), 4.36 (d, *J* = 5.2 Hz, 4 H), 1.72 – 1.87 (m, 2 H), 1.26 – 1.65 (m, 16 H), 0.97 (t, *J* = 7.3 Hz, 6 H), 0.88 (t, *J* = 7.3 Hz, 6 H) ppm. The analytical data are in agreement with those reported in the literature.[2]

3. Copies of the NMR spectra of compounds BDT-H2, O2, S2 AA, 6a-c and BDT-H1, O1, S1

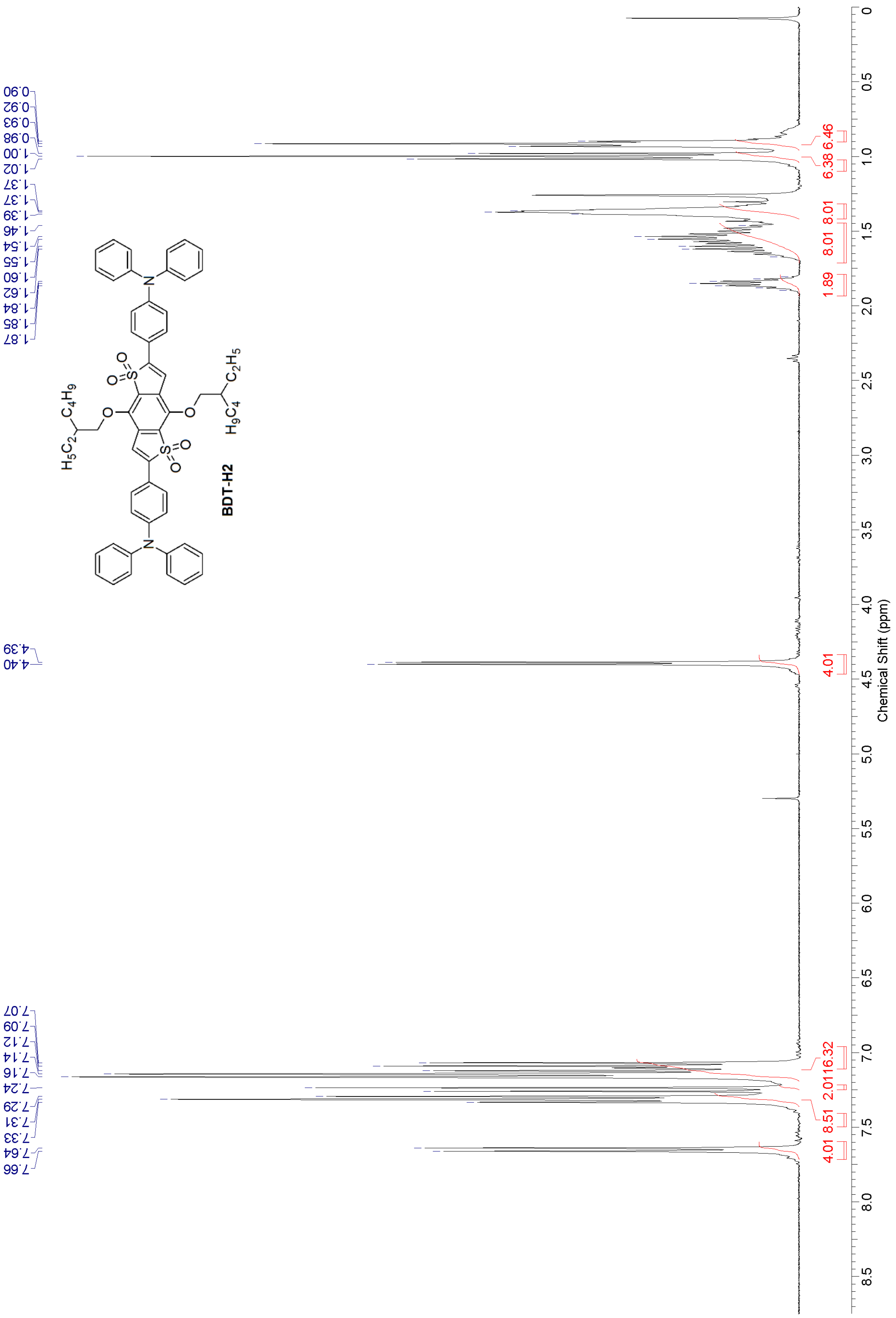

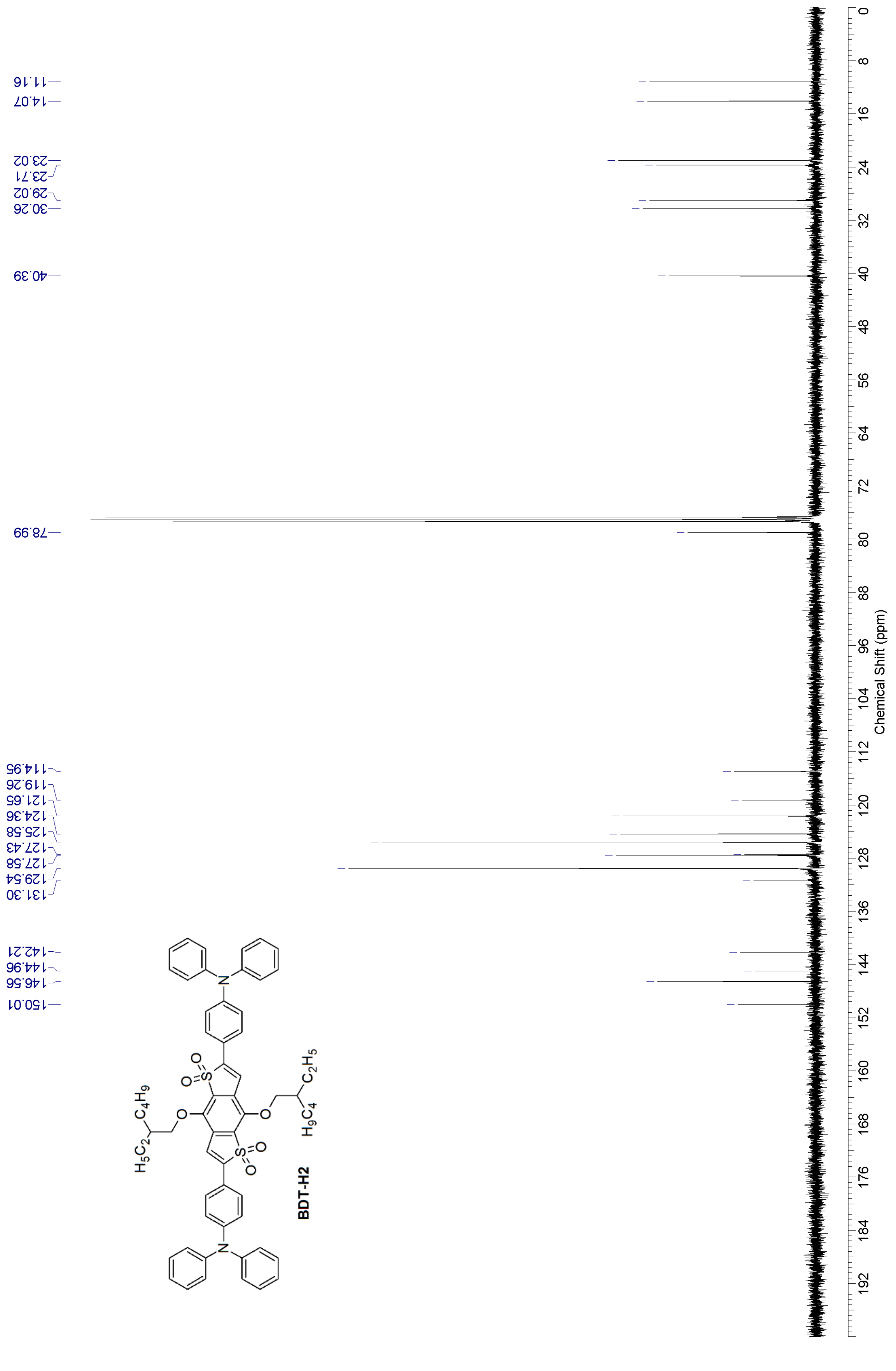
11.16
14.07
23.02
23.71
29.02
30.26
40.39
78.99
114.95
119.26
121.65
124.36
125.58
127.43
127.58
129.54
131.30
142.21
144.96
146.56
150.01
Chemical Shift (ppm)
BDT-H2

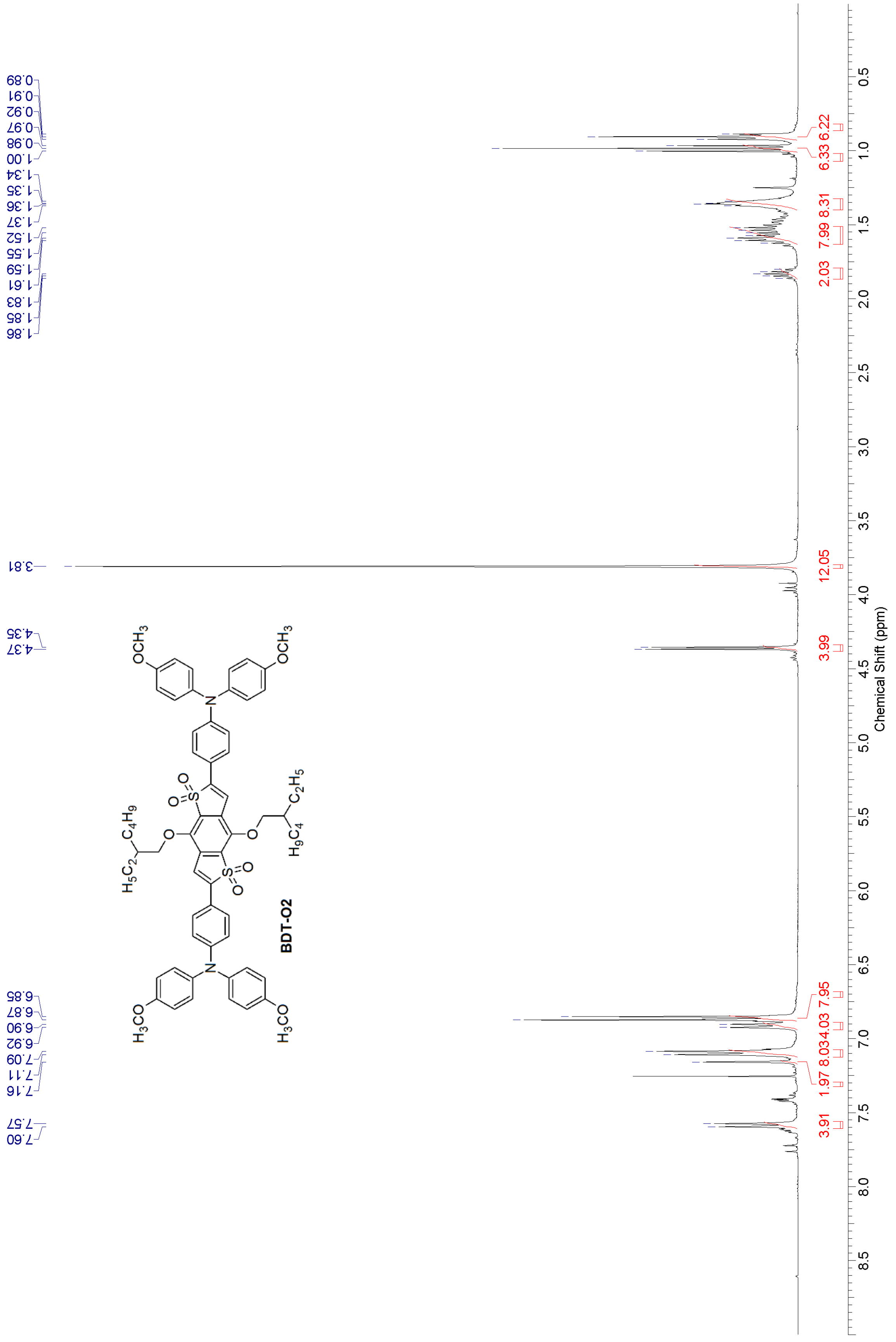
BDT-O2
Chemical Shift (ppm)

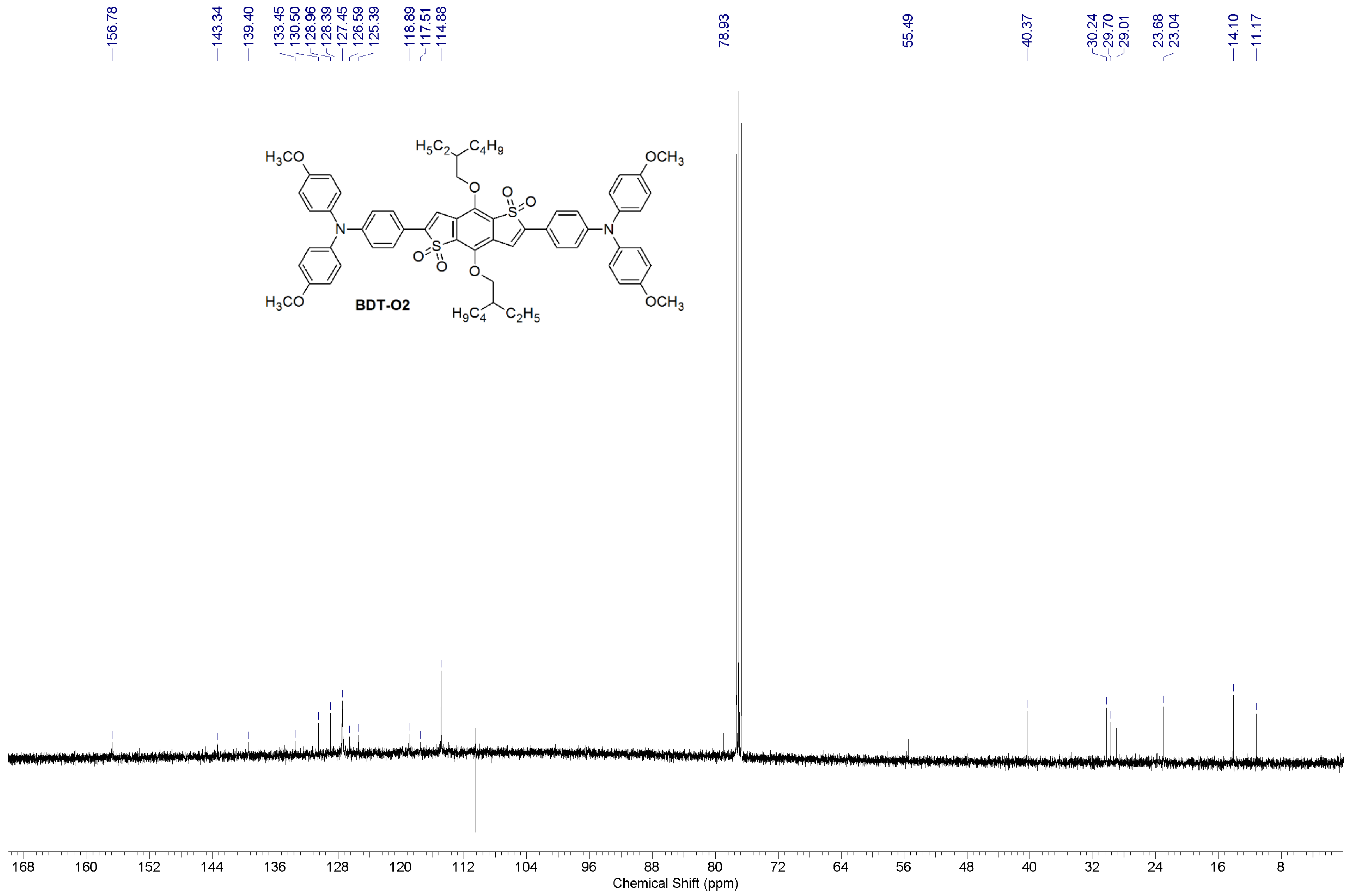
156.78
143.34
139.40
133.45
130.50
128.96
128.39
127.45
126.59
125.39
118.89
117.51
114.88
78.93
55.49
40.37
30.24
29.70
29.01
23.68
23.04
14.10
11.17
BDT-O2
168
160
152
144
136
128
120
112
104
96
88
80
72
64
56
48
40
32
24
16
8
Chemical Shift (ppm)

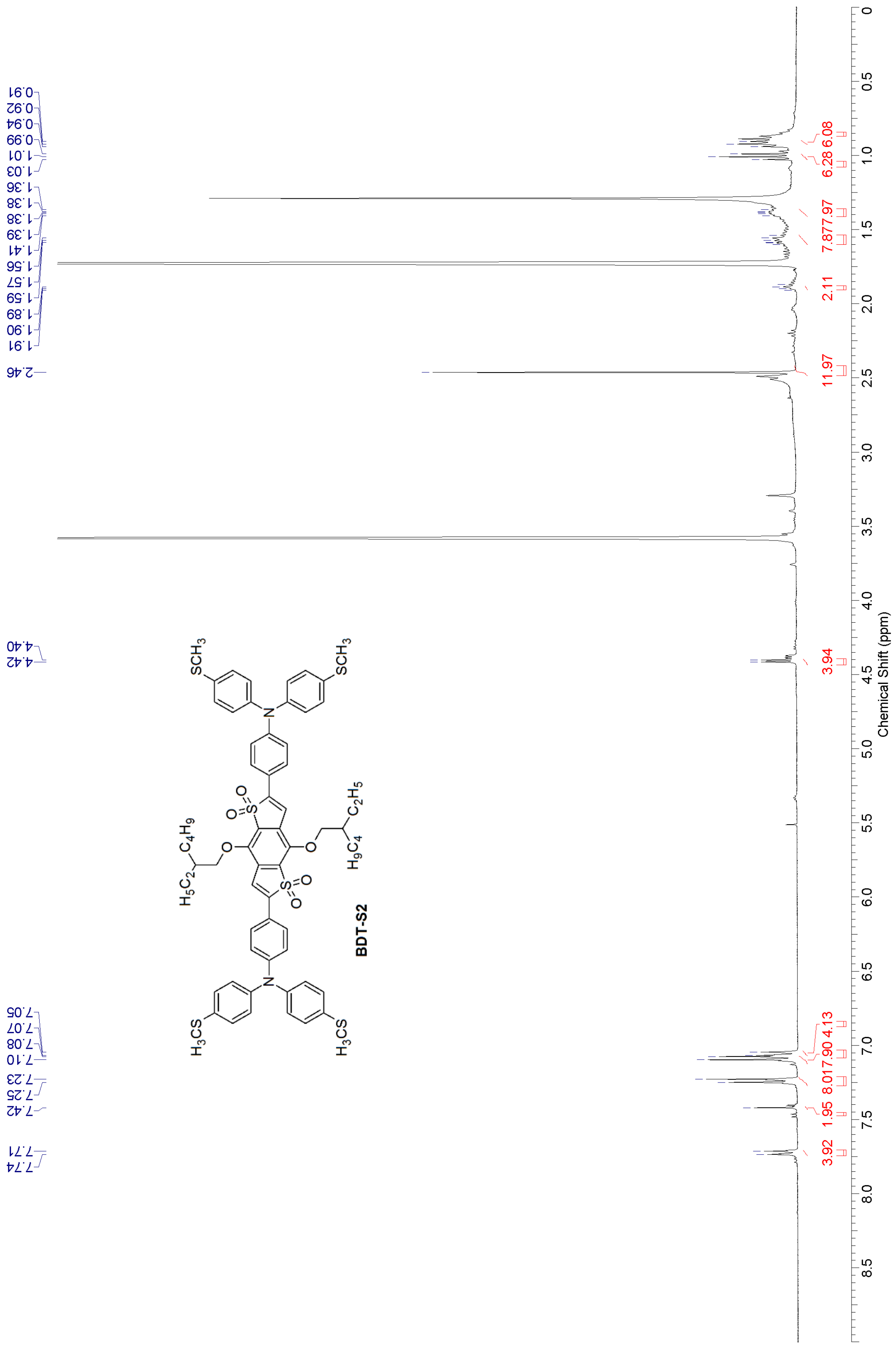
BDT-S2
Chemical Shift (ppm)

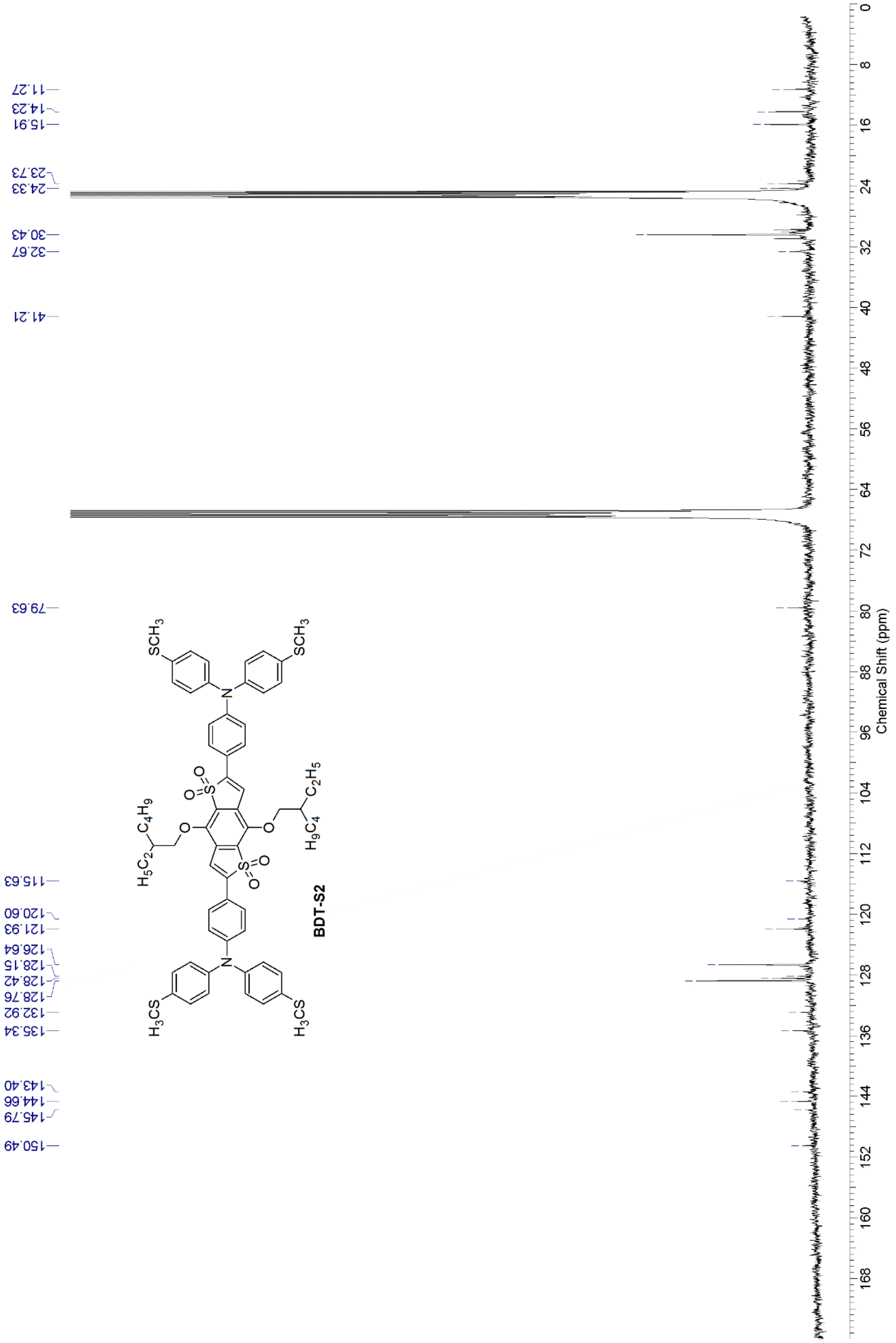
11.27
14.23
15.91
23.73
24.33
30.43
32.67
41.21
79.63
115.63
120.60
121.93
126.64
128.15
128.42
128.76
132.92
135.34
143.40
144.66
145.79
150.49
BDT-S2
Chemical Shift (ppm)

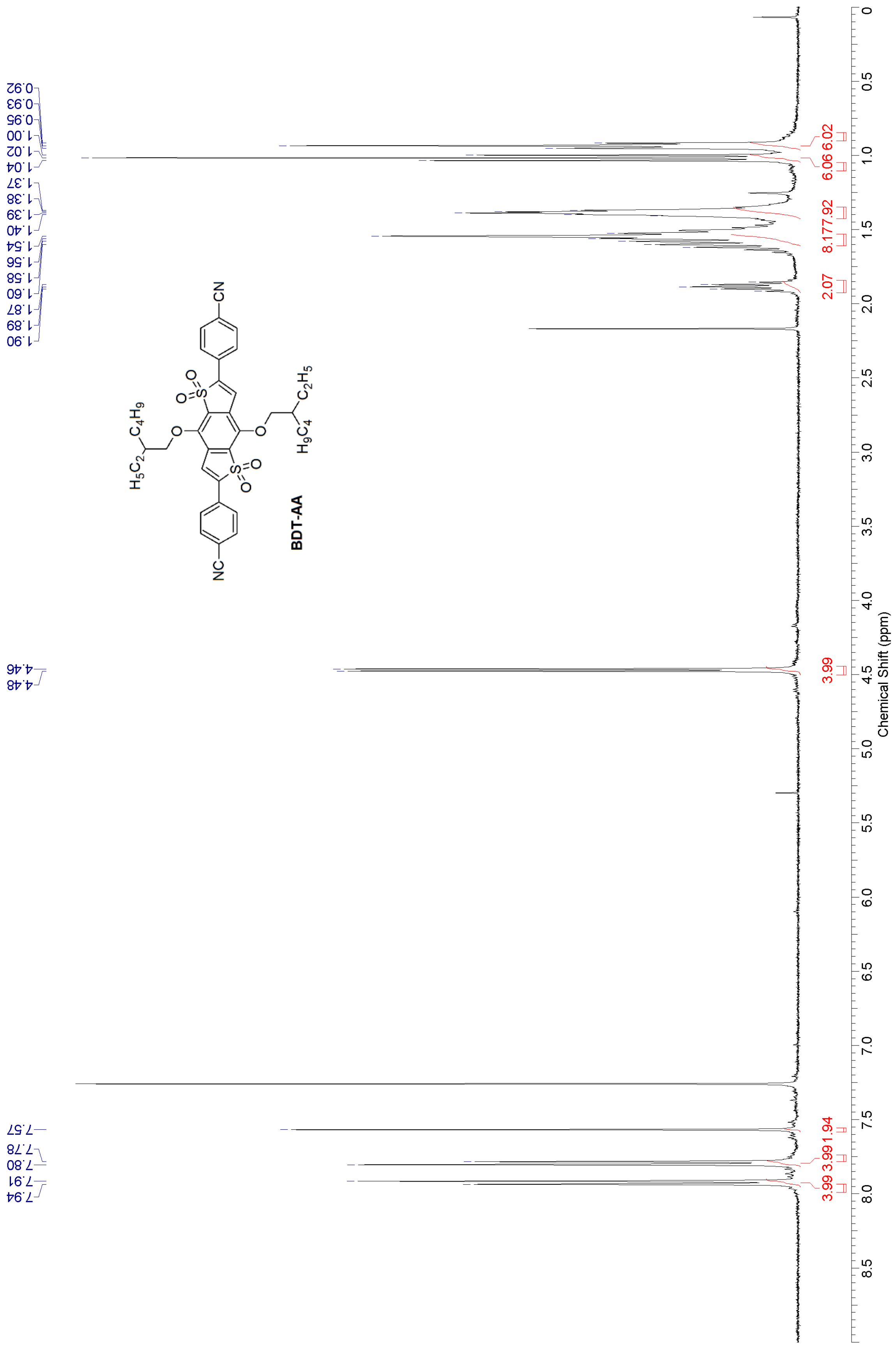

BDT-AA
NC
CN
H_5C_2
C_4H_9
H_9C_4
C_2H_5
Chemical Shift (ppm)
0.92 0.93 0.95 1.00 1.02 1.04 1.37 1.38 1.39 1.40 1.54 1.56 1.58 1.60 1.87 1.89 1.90
4.46 4.48
7.57 7.78 7.80 7.91 7.94
6.02 6.06 8.17 7.92 2.07 3.99 1.94 3.99 3.99

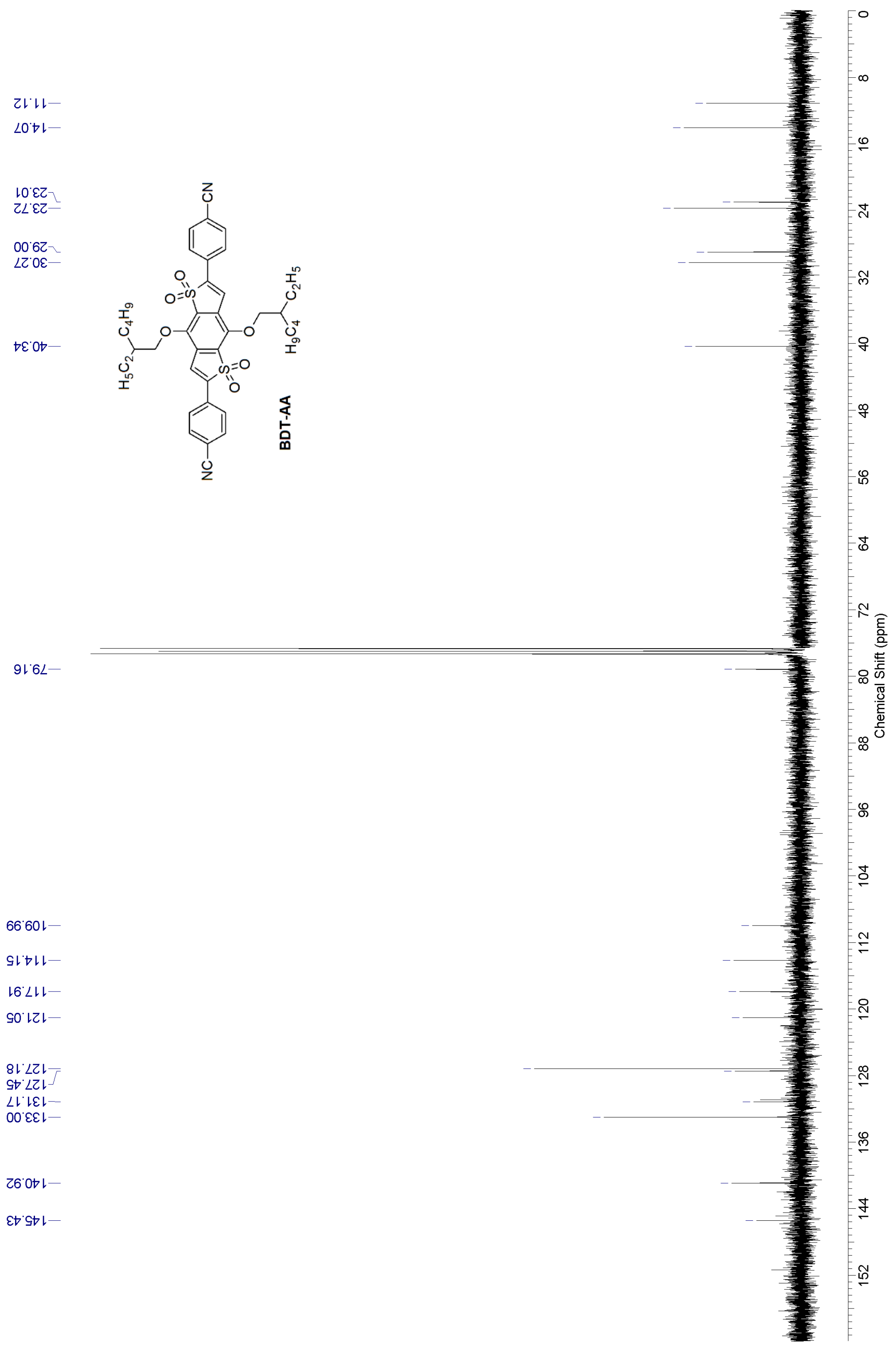
11.12
14.07
23.01
23.72
29.00
30.27
40.34
79.16
109.99
114.15
117.91
121.05
127.18
127.45
131.17
133.00
140.92
145.43
BDT-AA
0
8
16
24
32
40
48
56
64
72
80
88
96
104
112
120
128
136
144
152
Chemical Shift (ppm)

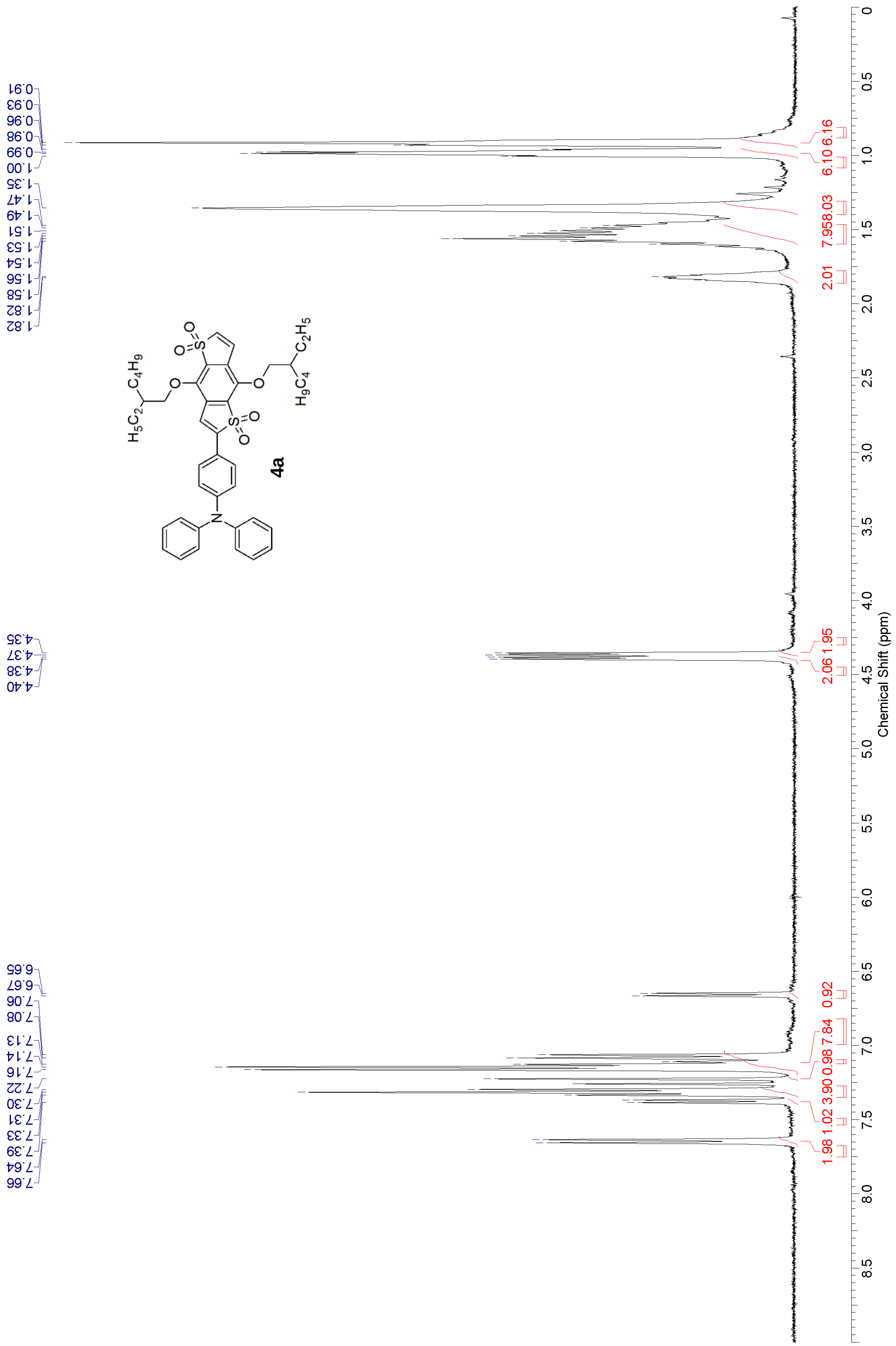
4a
Chemical Shift (ppm)

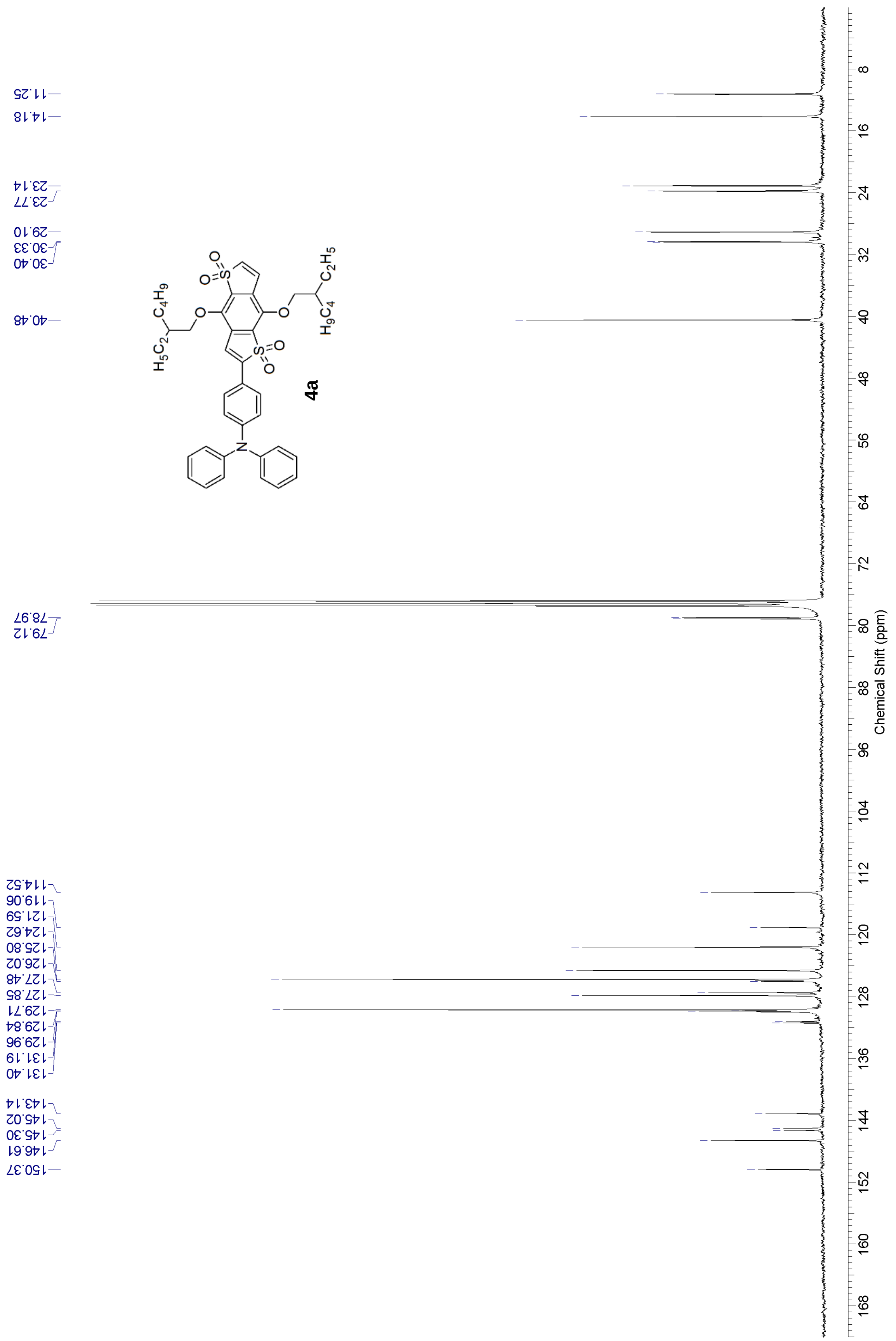

11.25
14.18
23.14
23.77
29.10
30.33
30.40
40.48
78.97
79.12
114.52
119.06
121.59
124.62
125.80
126.02
127.48
127.85
129.71
129.84
129.96
131.19
131.40
143.14
145.02
145.30
146.61
150.37
4a
Chemical Shift (ppm)

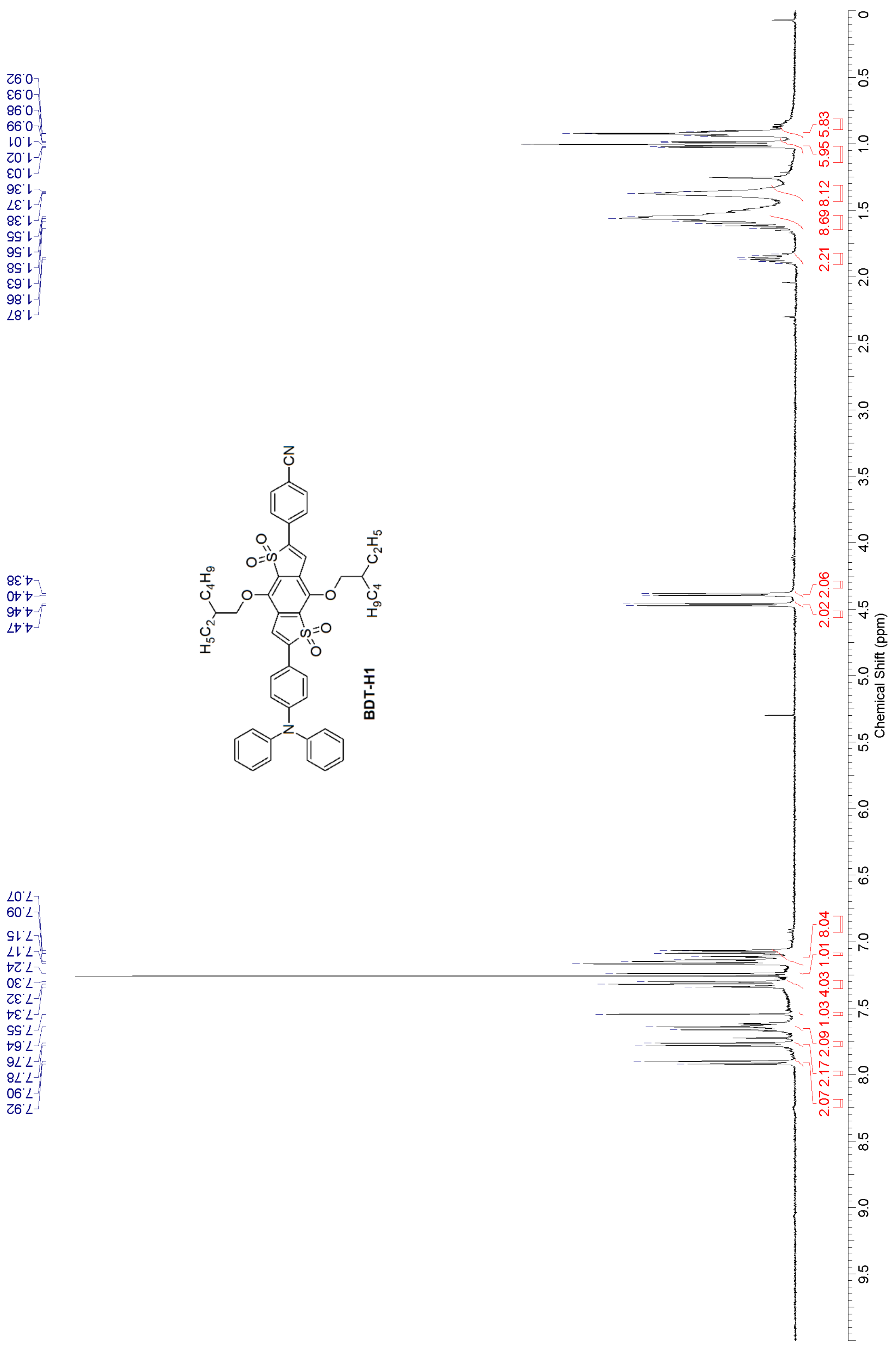
BDT-H1
Chemical Shift (ppm)

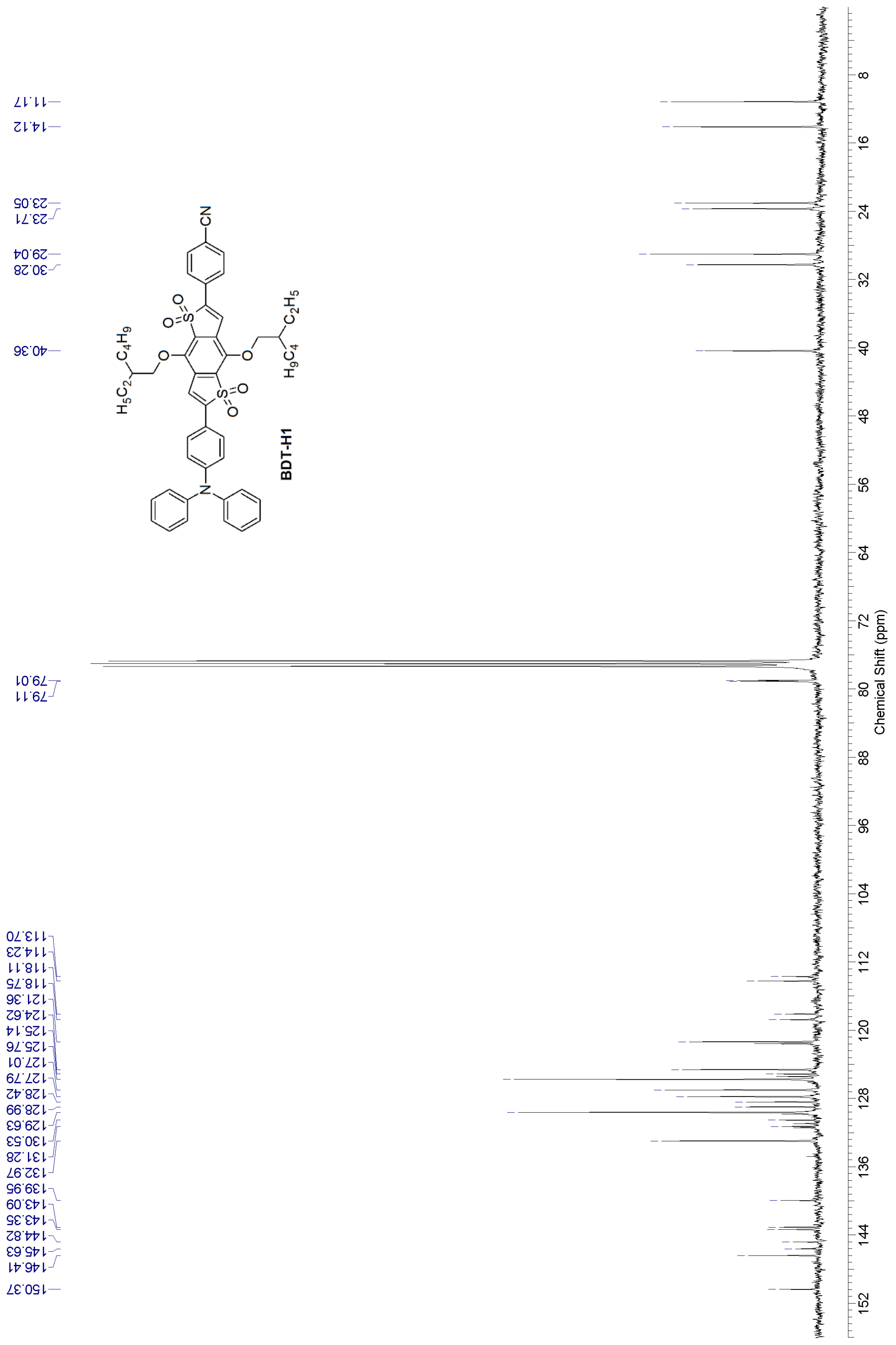
11.17
14.12
23.05
23.71
29.04
30.28
40.36
79.01
79.11
113.70
114.23
118.11
118.75
121.36
124.62
125.14
125.76
127.01
127.79
128.42
128.99
129.63
130.53
131.28
132.97
139.95
143.09
143.35
144.82
145.63
146.41
150.37
BDT-H1
Chemical Shift (ppm)

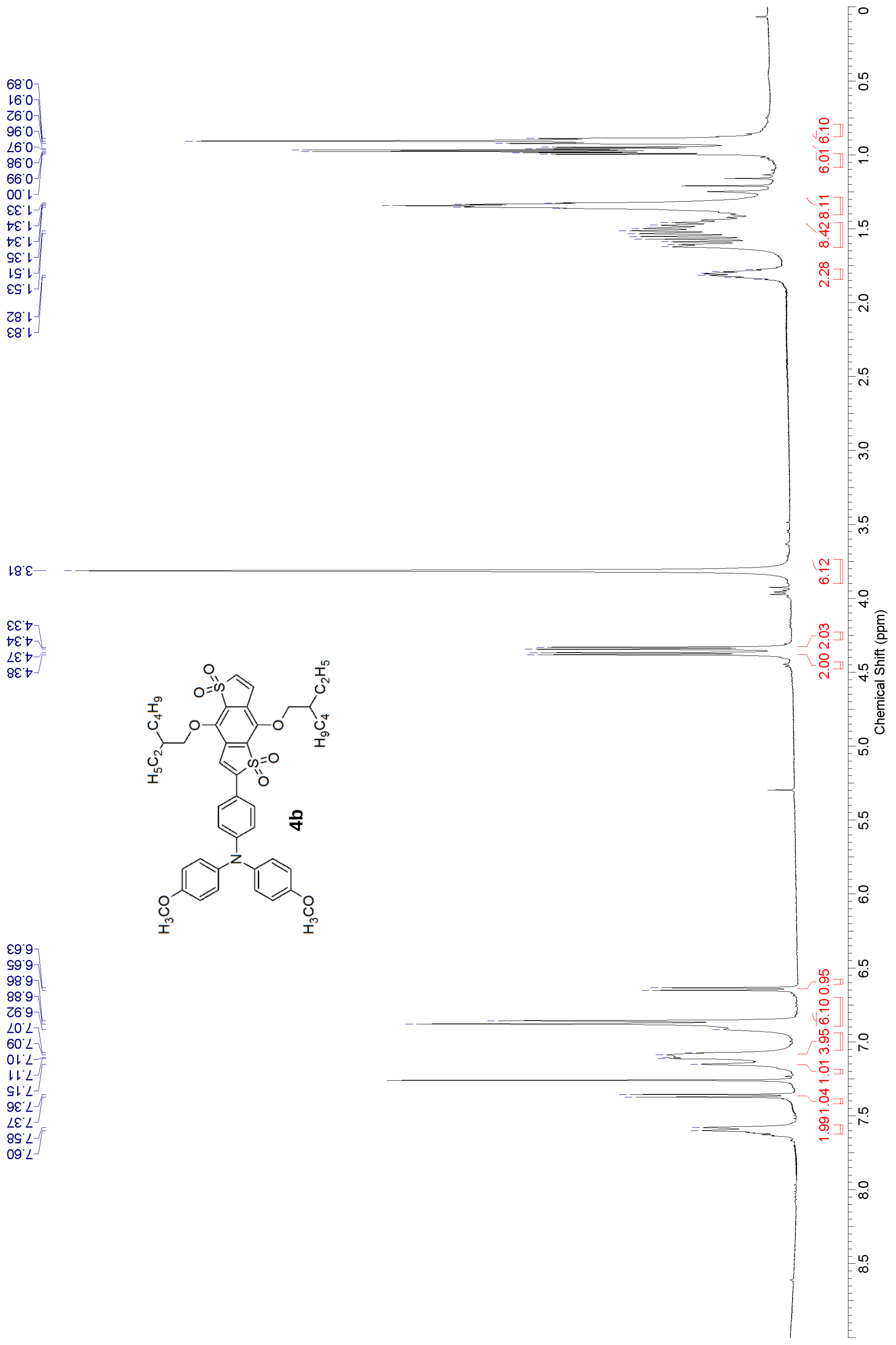
4b
Chemical Shift (ppm)

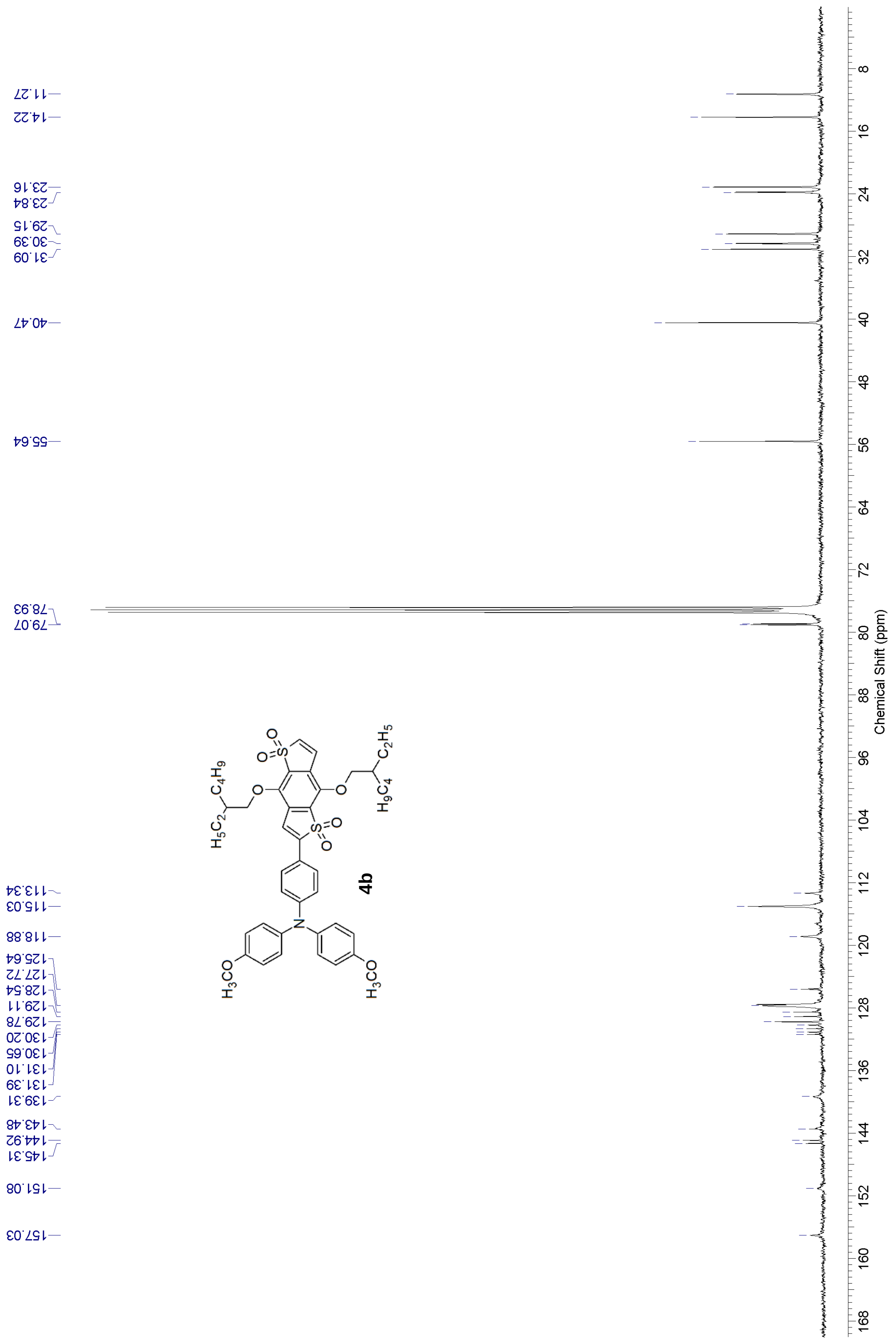
11.27
14.22
23.16
23.84
29.15
30.39
31.09
40.47
55.64
78.93
79.07
113.34
115.03
118.88
125.64
127.72
128.54
129.11
129.78
130.20
130.65
131.10
131.39
139.31
143.48
144.92
145.31
151.08
157.03
4b
Chemical Shift (ppm)

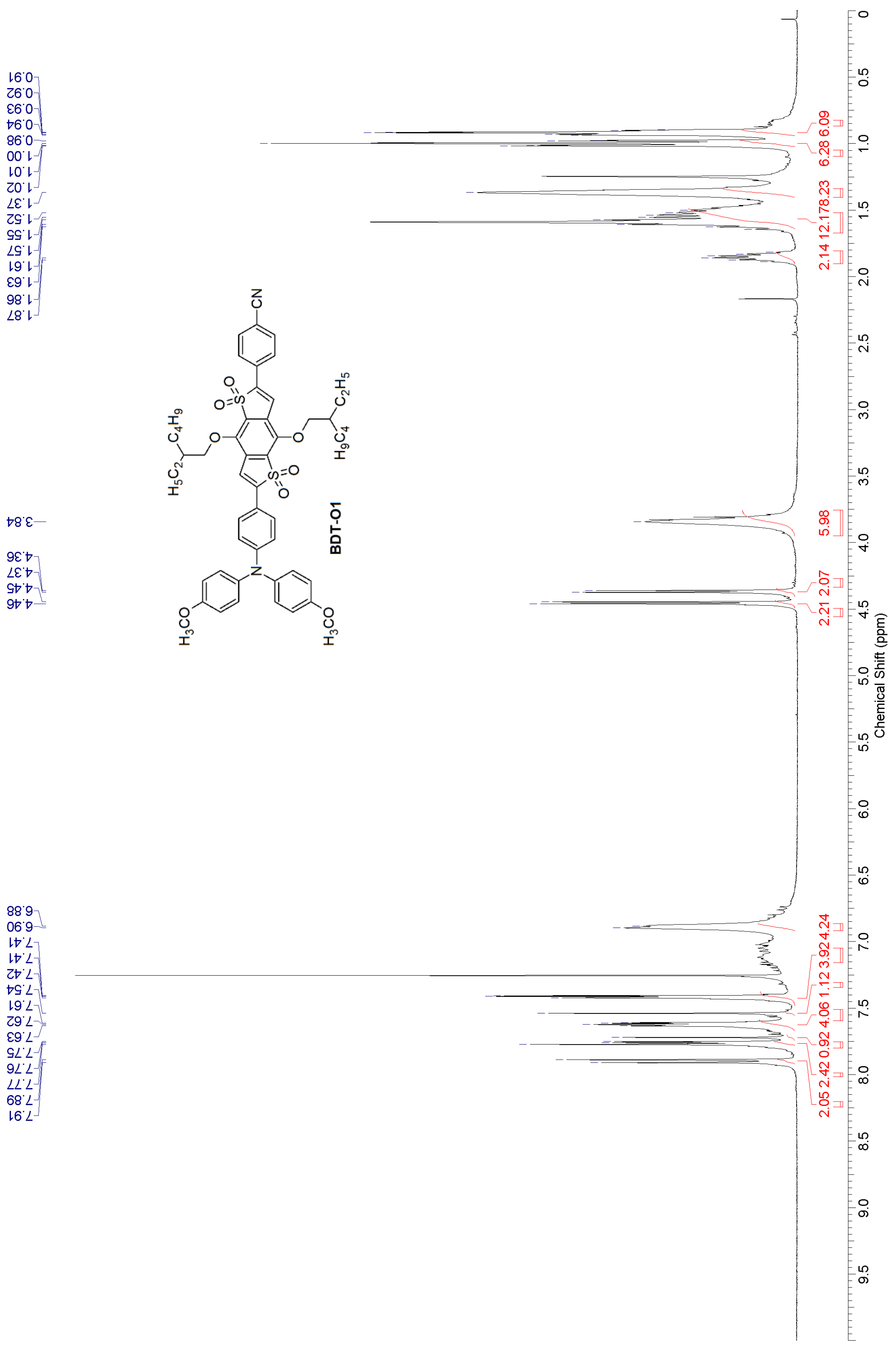
BDT-O1
Chemical Shift (ppm)

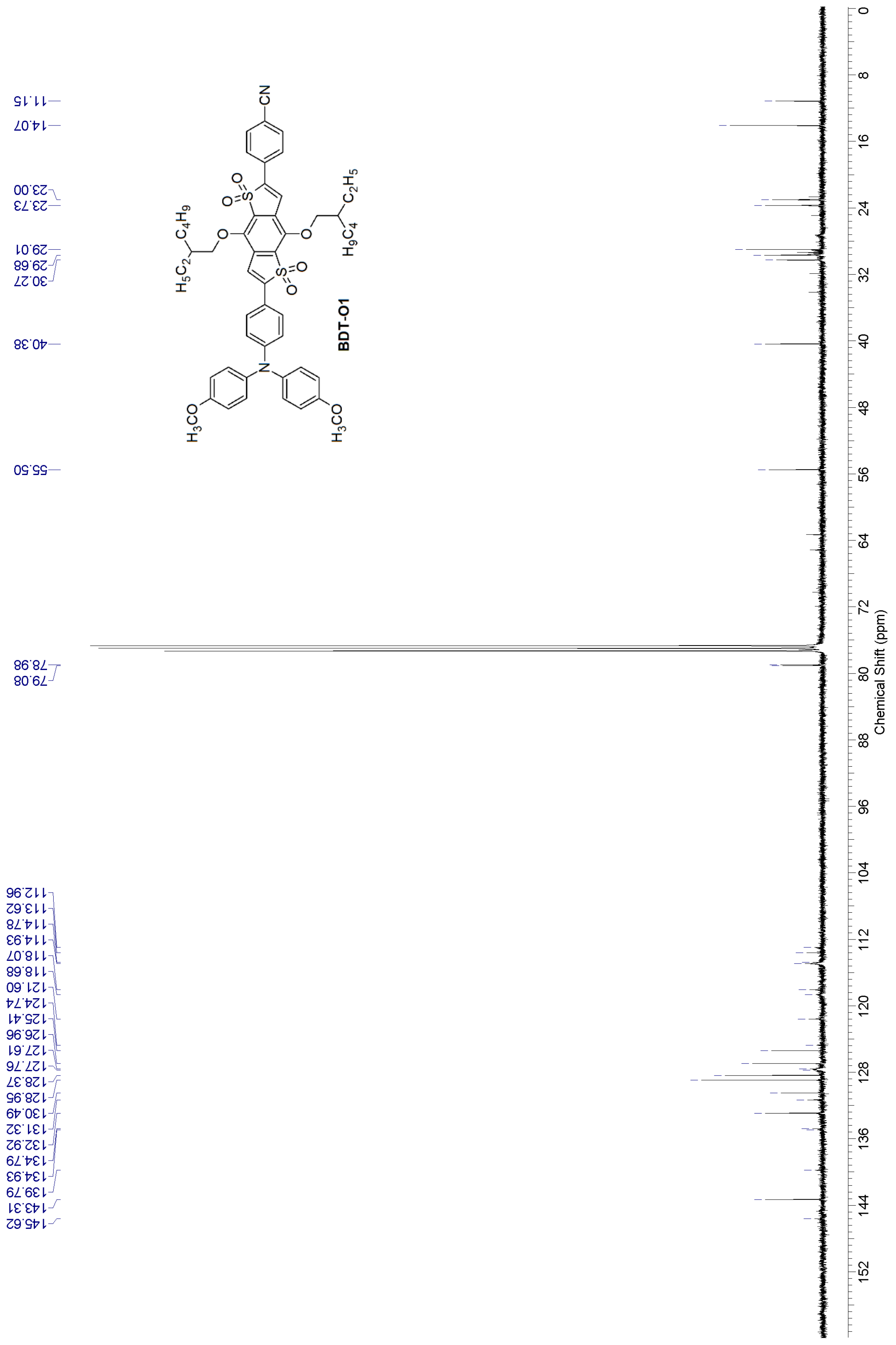

BDT-O1
CN
H3CO
H3CO
N
O
S
H5C2
C4H9
C2H5
H9C4
11.15
14.07
23.00
23.73
29.01
29.68
30.27
40.38
55.50
78.98
79.08
112.96
113.62
114.78
114.93
118.07
118.68
121.60
124.74
125.41
126.96
127.61
127.76
128.37
128.95
130.49
131.32
132.92
134.79
134.93
139.79
143.31
145.62
Chemical Shift (ppm)
0
8
16
24
32
40
48
56
64
72
80
88
96
104
112
120
128
136
144
152

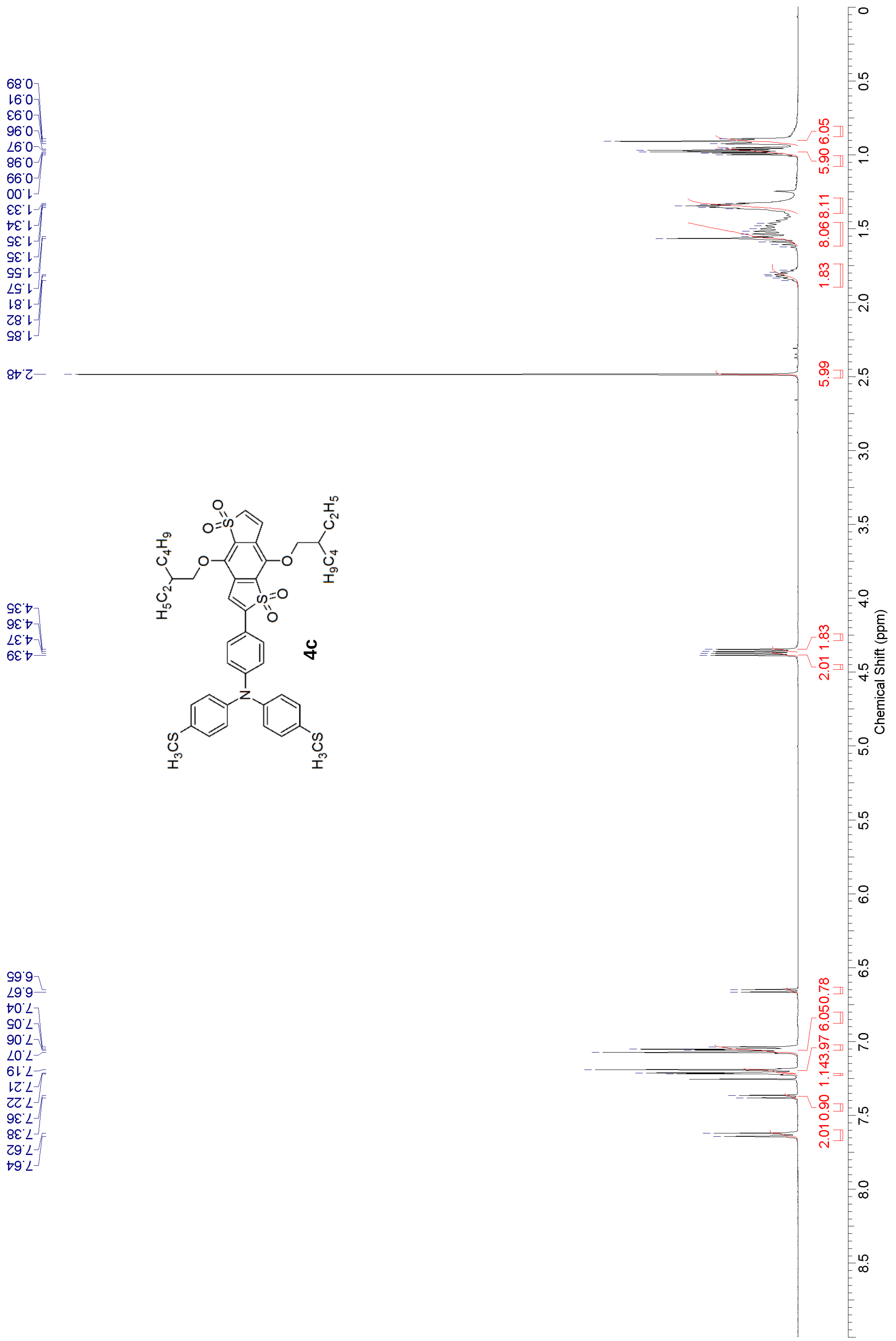
4c
Chemical Shift (ppm)

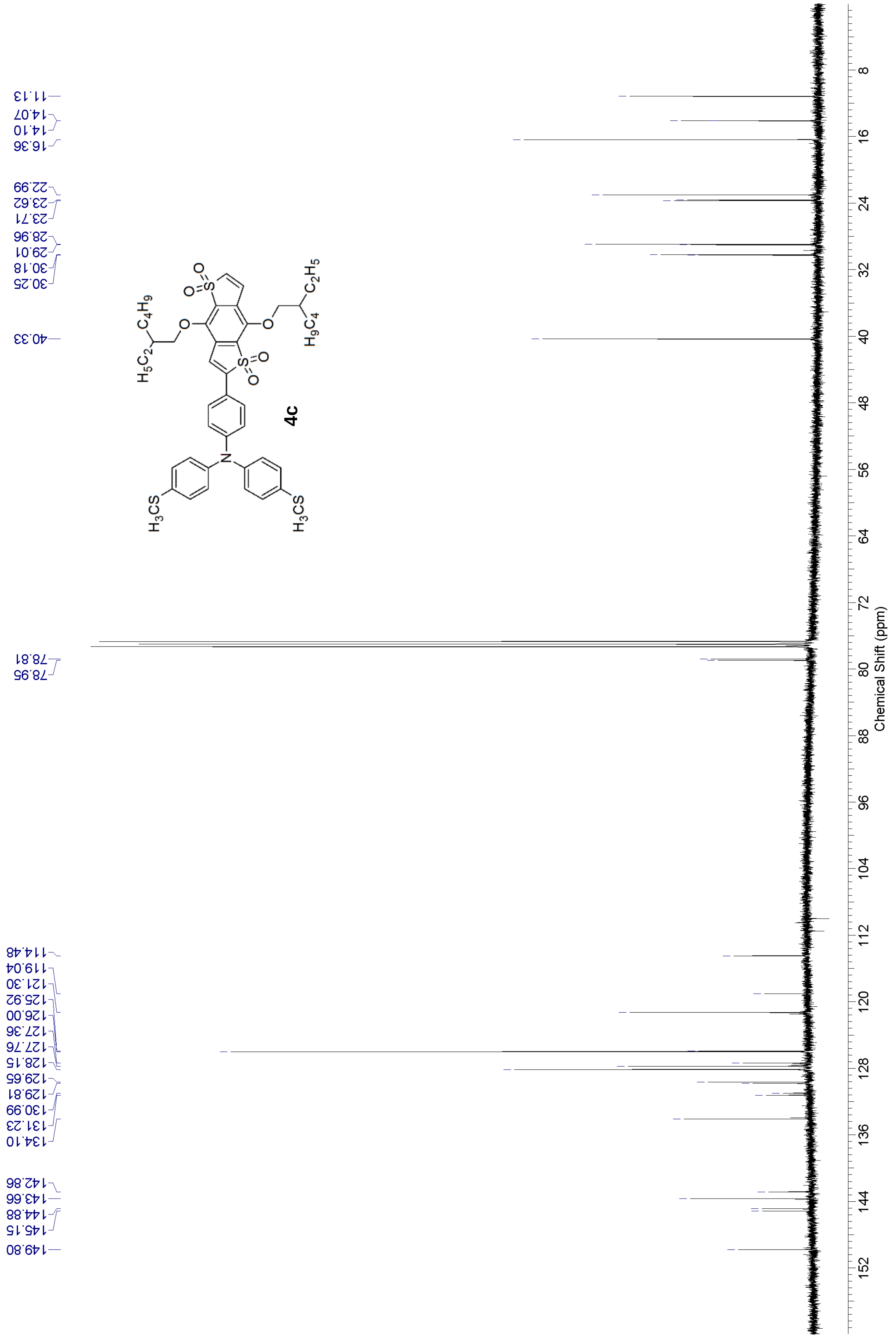
11.13
14.07
14.10
16.36
22.99
23.62
23.71
28.96
29.01
30.18
30.25
40.33
78.81
78.95
114.48
119.04
121.30
125.92
126.00
127.36
127.76
128.15
129.65
129.81
130.99
131.23
134.10
142.86
143.66
144.88
145.15
149.80
4c
Chemical Shift (ppm)

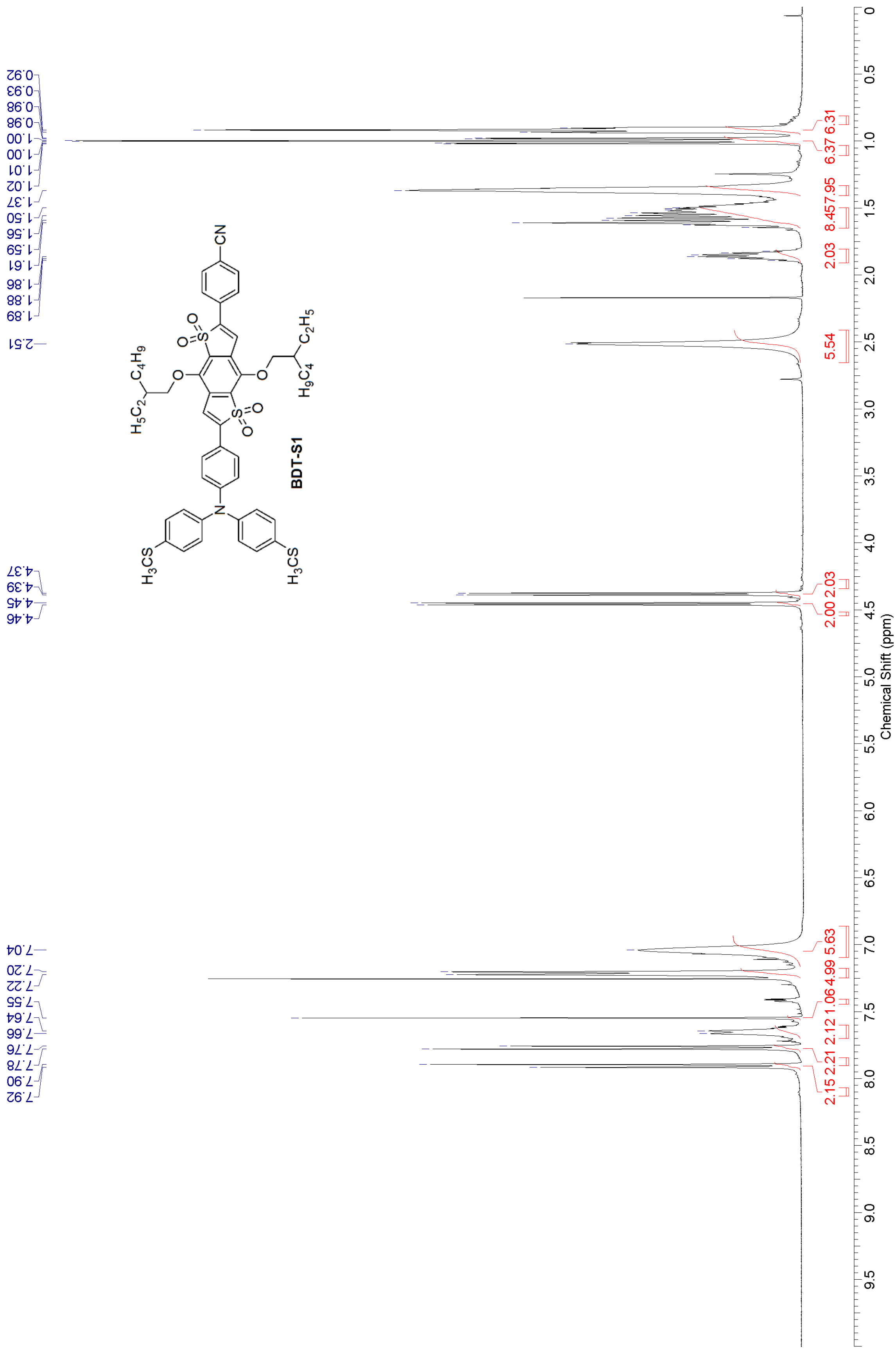
BDT-S1
Chemical Shift (ppm)

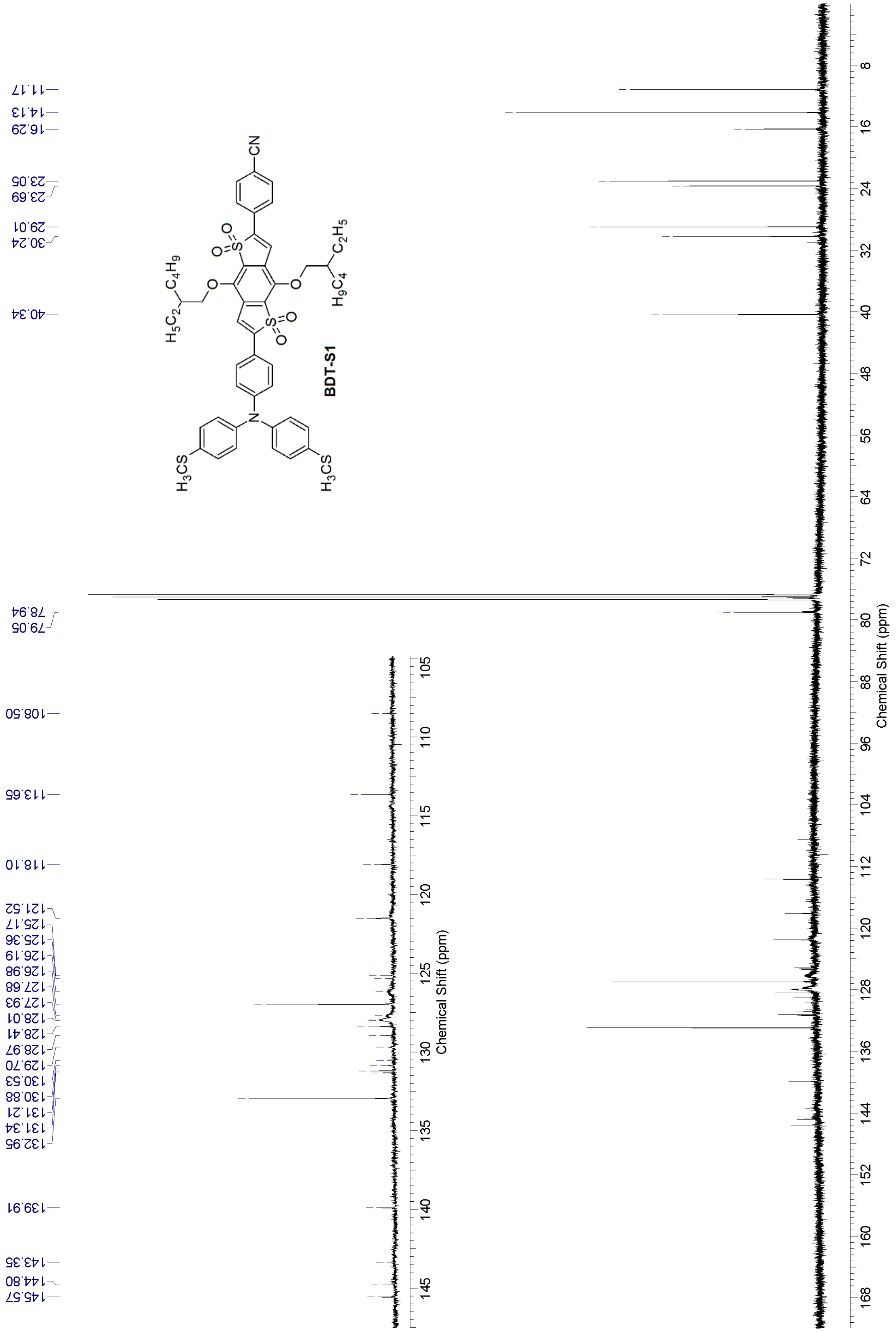
11.17
14.13
16.29
23.05
23.69
29.01
30.24
40.34
78.94
79.05
108.50
113.65
118.10
121.52
125.17
125.36
126.19
126.98
127.68
127.93
128.01
128.41
128.97
129.70
130.53
130.88
131.21
131.34
132.95
139.91
143.35
144.80
145.57
CN
H_5C_2
C_4H_9
C_2H_5
H_9C_4
H_3CS
BDT-S1
Chemical Shift (ppm)
Chemical Shift (ppm)

4. Spectroscopic characterization in different solvents

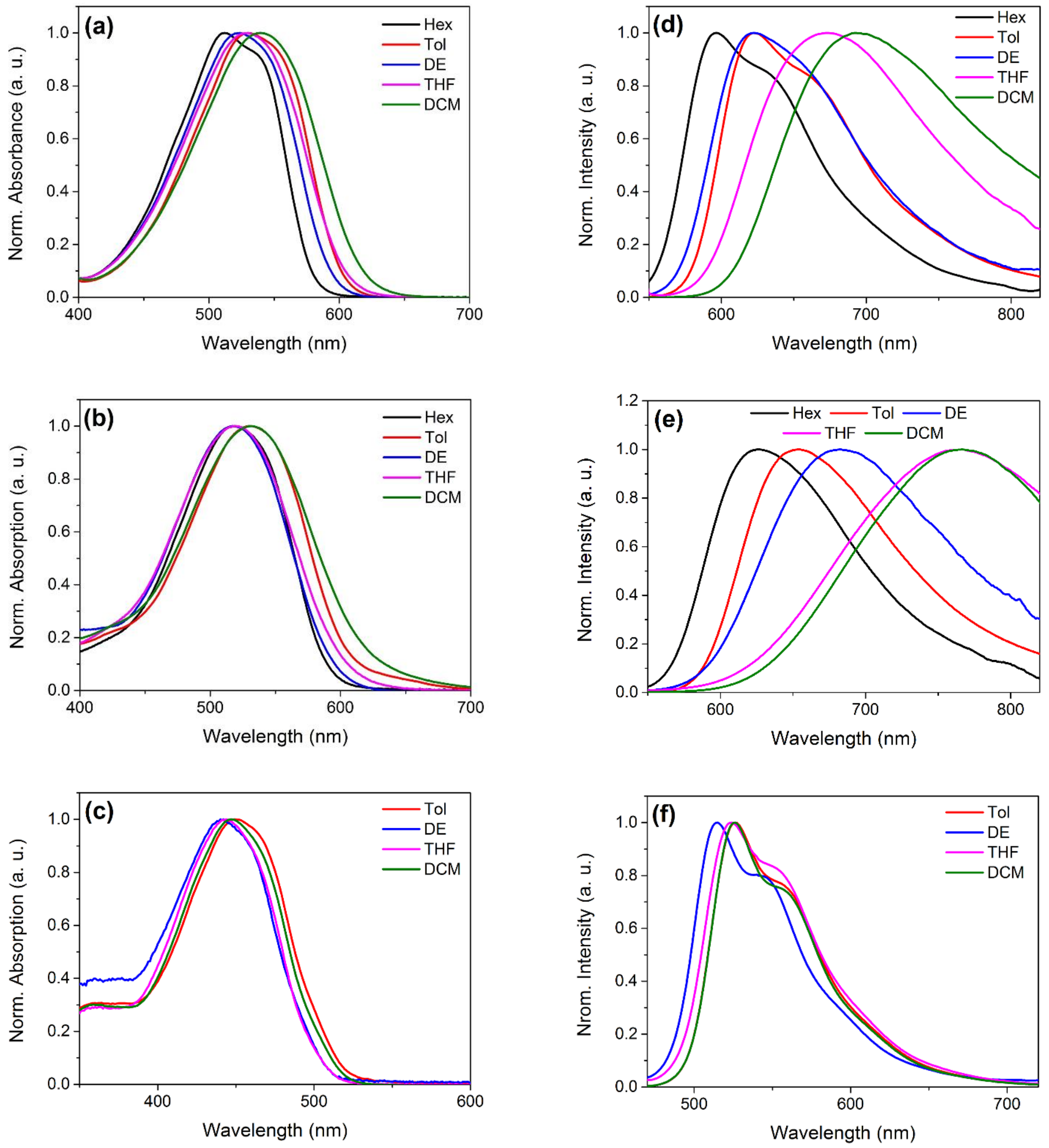

Figure S4. *Normalized UV-Vis absorption (a-c) and fluorescence emission (d-f) spectra of compounds **BDT-H2** (a,d), **BDT-H1** (b,e) and **BDT-AA** (c,f) in different solvents. Hex: n-hexane (black line); Tol: toluene (red line); DE: diethyl ether (blue line); THF: tetrahydrofuran (magenta line); DCM: dichloromethane (green line).*

Table S3. *Spectroscopic properties of compounds **BDT-H2**, **BDT-H1** and **BDT-AA** in solvents of different polarity.*

Solvent	$\varepsilon \times 10^4$ [M^{-1} cm^{-1}]	λ^{abs}_{max} [nm]	λ^{emi}_{max} [nm]	Φ_f [%][b]	*SS* [nm] {eV}[c]
			**BDT-H2**[a]		
Hexane	6.57	512	597	78	85 {0.34}
Toluene	6.35	530	622	77	92 {0.35}
Diethyl ether	4.83	524	623	74	99 {0.38}
Tetrahydrofuran	5.89	530	673	40	143 {0.50}
Dichloromethane	6.37	540	692	39	152 {0.50}
			**BDT-H1**		
Hexane	2.00	520	626	75	106 {0.40}
Toluene	2.39	531	654	68	123 {0.44}
Diethyl ether	2.88	517	683	45	166 {0.58}
Tetrahydrofuran	1.81	520	765[d]	5	245 {0.76}[d]
Dichloromethane	2.42	532	766[d]	4	232 {0.71}[d]
			**BDT-AA**		
Hexane[e]	-	-	-	-	-
Toluene	1.34	449	526	66	77 {0.40}
Diethyl ether	0.75	440	515	57	75 {0.41}
Tetrahydrofuran	1.14	444	524	53	80 {0.43}
Dichloromethane	1.28	447	524	66	77 {0.41}

[a] In good agreement with literature data.[4] [b] Absolute QY determined using an integrating sphere. [c] Stokes shift; [d] In these solvents, due to the low fluorescence yield and longer emission wavelengths of **BDT-H1**, the reported λ^{emi}_{max} and Stokes shift values are likely overestimated, as a result of the application of the correction function necessary to compensate the sensitivity loss of the photomultiplier in the red wavelength region. [e] No spectrum could be recorded due to the insufficient solubility of **BDT-AA** in this solvent.

5. Additional figures and tables for the TAS measurements

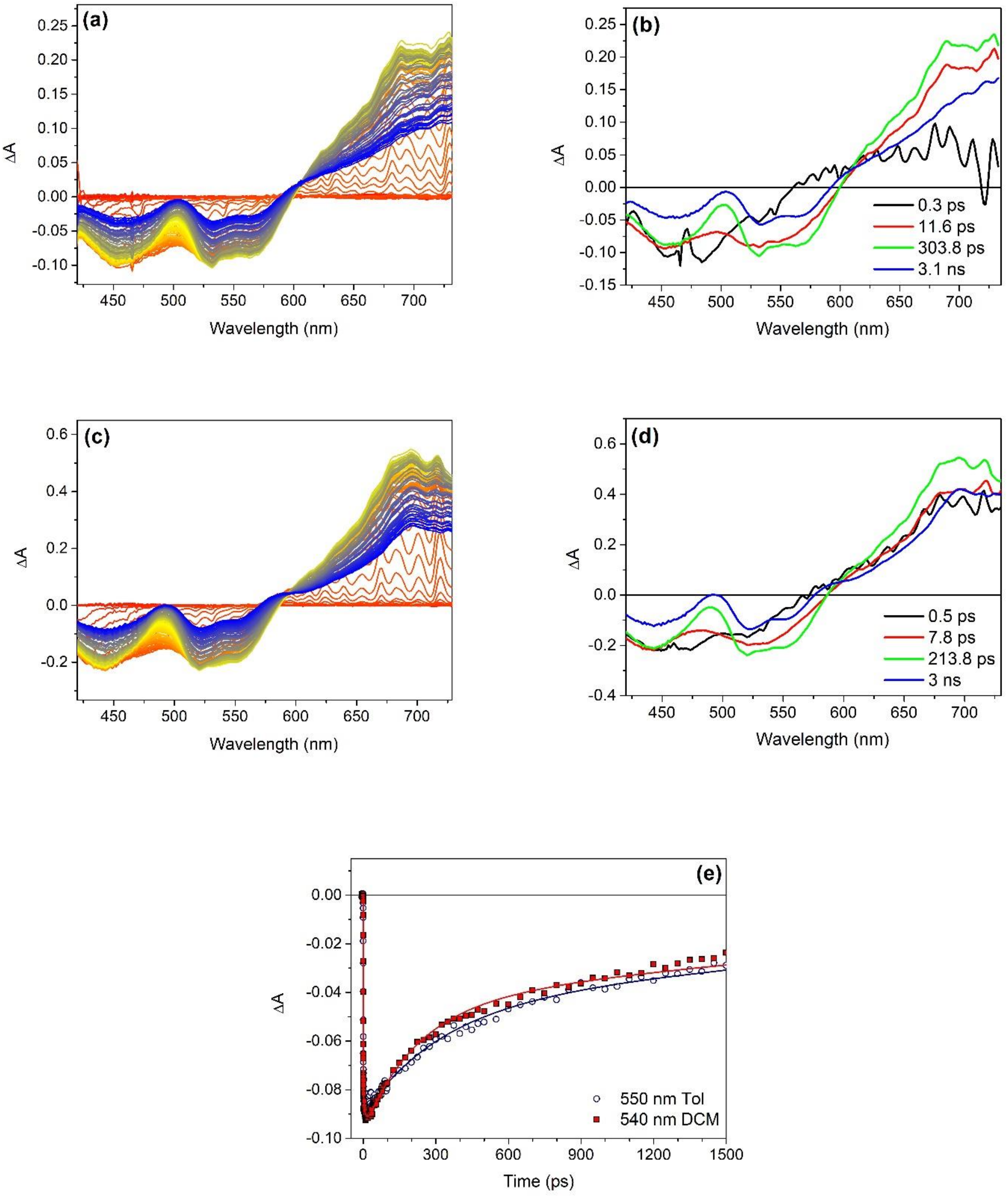

Figure S5. *Transient spectra measured for* ***BDT-AA*** *in (a) toluene and (c)* CH_2Cl_2 *and respective EADS obtained from global analysis, (b) and (d). (e) Kinetic traces registered on the bleaching bands for molecule* ***BDT-AA*** *in toluene and* CH_2Cl_2*. The continuous lines represent the fits obtained by global analysis.*

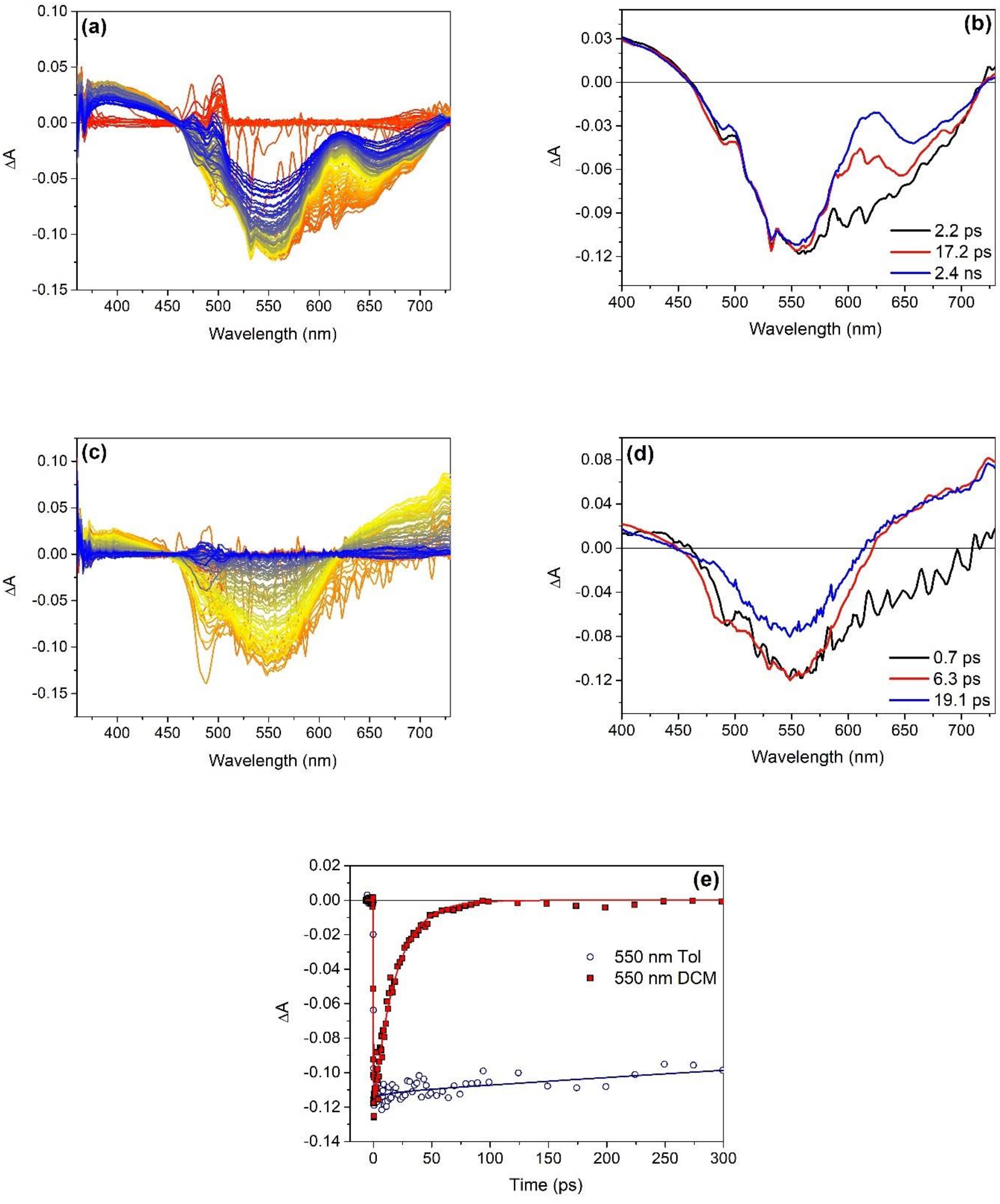

Figure S6. *Transient spectra measured for compound **BDT-O2** in (a) toluene and (c) CH_2Cl_2 and respective EADS obtained from global analysis, (b) and (d). (e) Kinetic traces registered on the bleaching bands for molecule **BDT-O2** in toluene and CH_2Cl_2. The continuous lines represent the fits obtained by global analysis.*

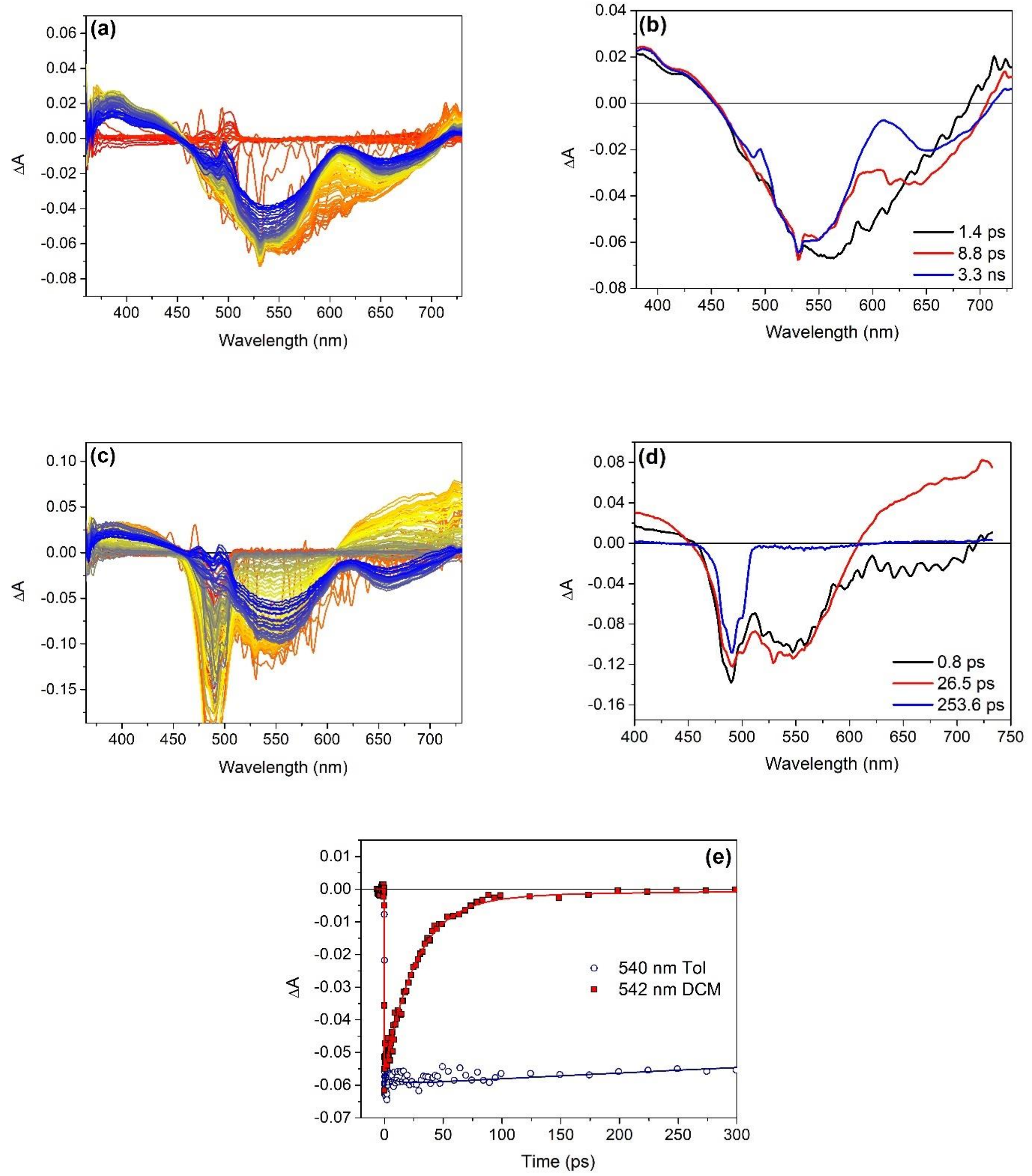

Figure S7. Transient spectra measured *for compound **BDT-S2** in (a) toluene and (b)* CH_2Cl_2 *and respective EADS obtained from global analysis (c) and (d). (e) Kinetic traces registered on the bleaching bands for molecule **BDT-S2** in toluene and* CH_2Cl_2*. The continuous line represents the fit obtained by global analysis.*

Table S4. *Lifetimes obtained from global analysis of the transient data of the compounds in toluene and CH_2Cl_2.*

**Compound**	**Toluene**				**CH_2Cl_2**			
	τ_1	τ_2	τ_3	τ_4	τ_1	τ_2	τ_3	τ_4
**BDT-H1**	0.85 ps	9.3 ps	5.6 ns	-	0.8 ps	5.9 ps	868 ps	-
**BDT-H2**	1.0 ps	12 ps	3.0 ns	-	0.4 ps	1.3 ps	4.0 ns	-
**BDT-O2**	2.2 ps	17.3 ps	2.4 ns	-	0.6 ps	6.3 ps	19.1 ps	-
**BDT-S2**	1.4 ps	8.8 ps	3.2 ns	-	0.8 ps	26.6 ps	253 ps	-
**BDT-AA**	0.3 ps	11.6 ps	304 ps	3.1 ns	0.5 ps	7.8 ps	214 ps	3.0 ns

6. Characterization of fluorophore-doped PMMA films

BDT-O2

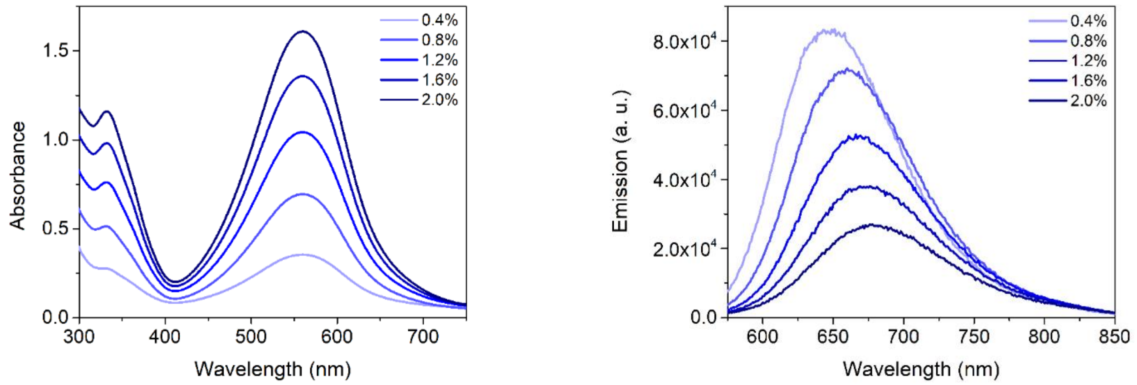

BDT-S2

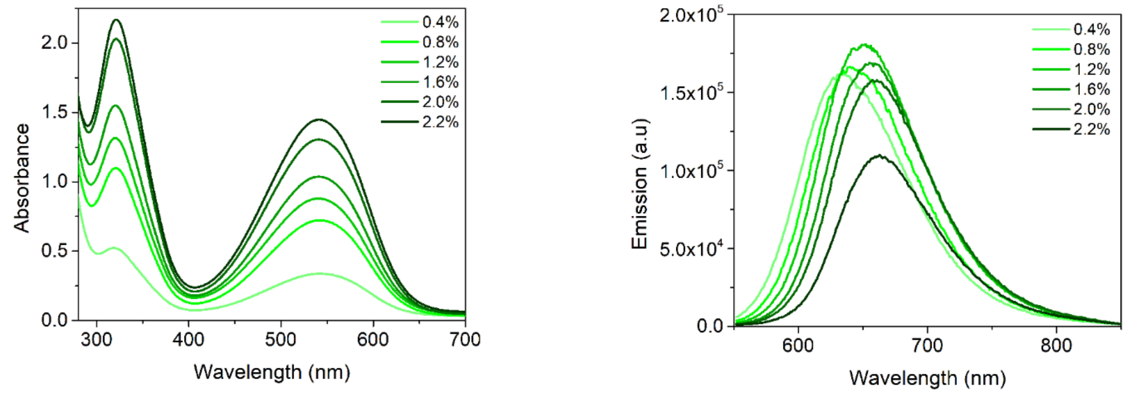

BDT-H1

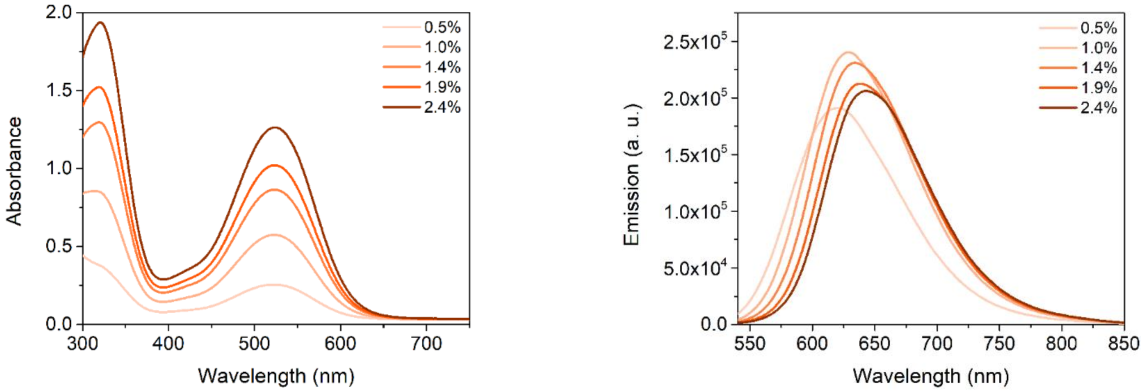

BDT-AA

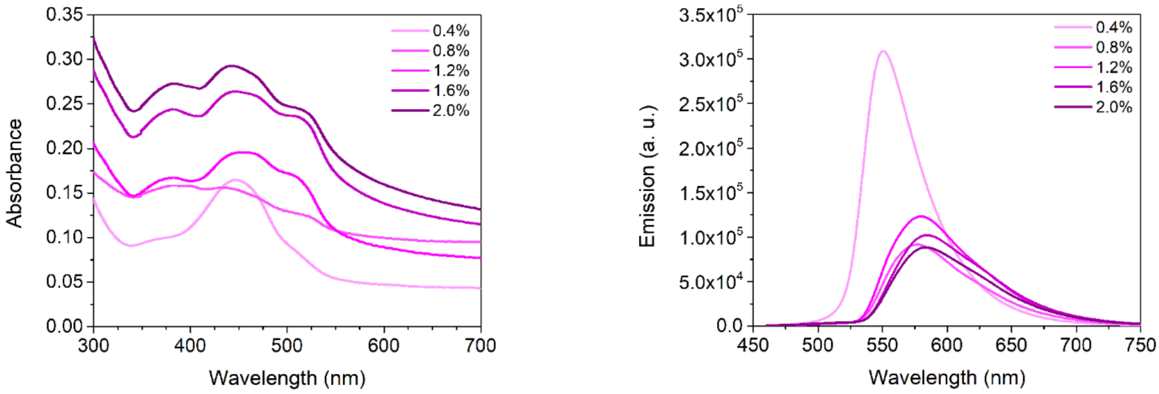

Figure S8. *UV-Vis absorption (left) and fluorescence emission (right) spectra of PMMA films containing compounds **BDT-O2, S2, H1** and **AA** at different concentrations.*

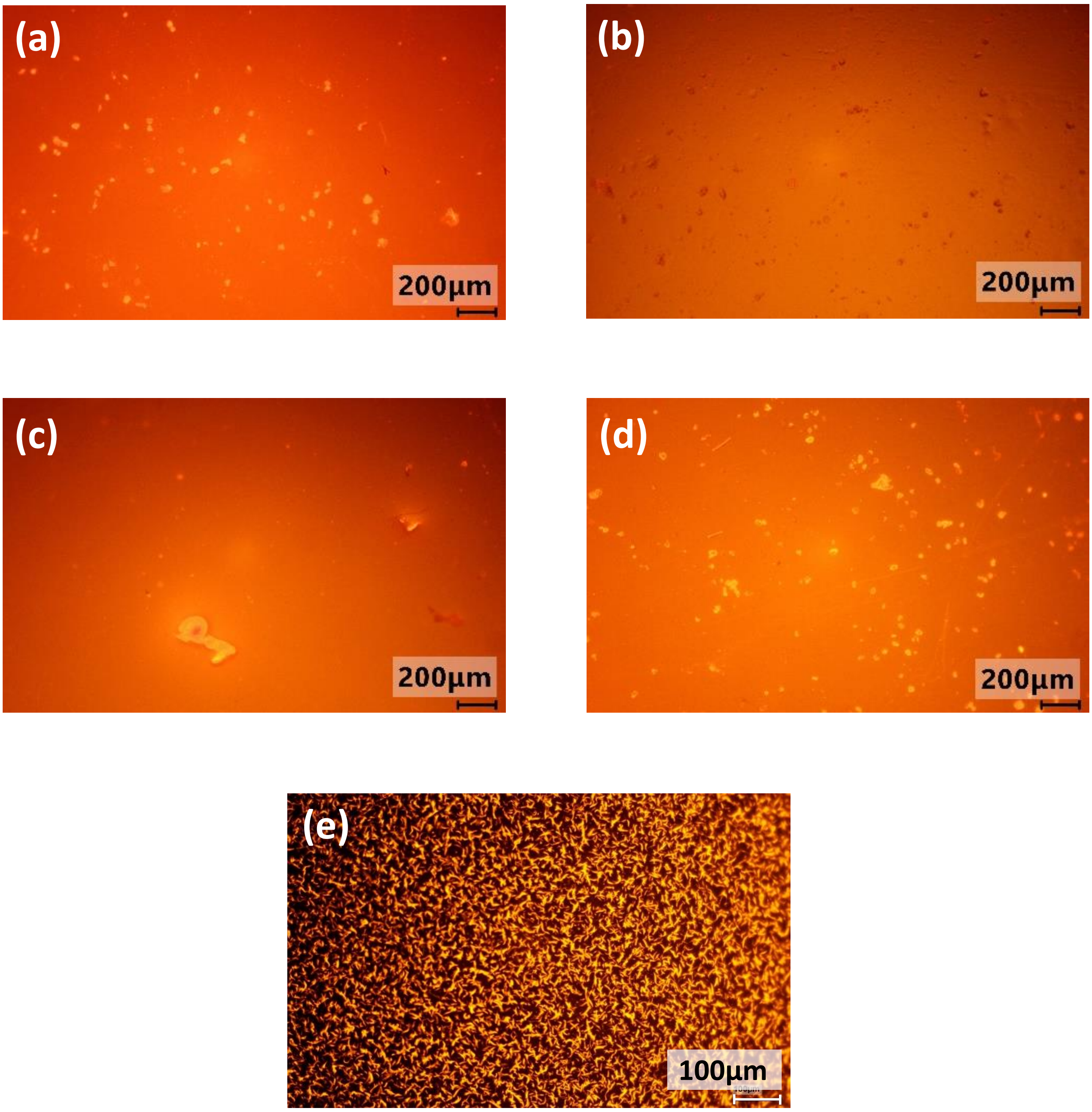

Figure S9. *Epifluorescence microscopy images of PMMA films doped with the fluorophores at the following concentrations: (a)* ***BDT-H2****, 0.6 wt.%; (b)* ***BDT-O2****, 0.4 wt.%; (c)* ***BDT-S2****, 0.4 wt%; (d)* ***BDT-H1****, 0.5 wt.%; (e)* ***BDT-AA****, 0.4 wt.%. Scale bars (100-200 µm) are indicated at the bottom left corner of each panel.*

7. Absorption efficiency calculation

To provide a quantitative evaluation of the spectral match between the absorption of the organic fluorophores prepared in this study and the emission of the solar simulator lamp used to characterize the LSCs (see below, Figure S11), absorption efficiency (η_{abs}) was calculated according to the method described by Debije *et al.* in their reference work.[5]
The expression used for calculation was:

$$\eta_{abs} = \frac{\int_{300}^{800} S_{so}(\lambda)\cdot(1-10^{-A(\lambda)})d\lambda}{\int_{300}^{800} S_{so}(\lambda)d\lambda} \qquad \text{(Eq. S1)}$$

Where $S_{so}(\lambda)$ is the emission spectrum of the light source and $A(\lambda)$ is the absorbance of the fluorophore dispersed in the polymeric matrix, both wavelength-dependent. The result is a numerical value that is dependent on luminophore concentration. The integration limits were selected to account for the spectral range of the luminophore and the emission range of the light source.
The curves were plotted against the maximum absorbance at each concentration (A_{max}), and fitted to the following exponential functions, as previously described:[6]

$$\eta_{abs} = a(1 - e^{-bA_{\max}}) \qquad \text{(Eq. S2)}$$

Fitting results are as follows:

BDT-H2: *a* = 0.53; *b* = 1.38; R^2 = 0.991

BDT-O2: *a* = 0.67; *b* = 1.60; R^2 = 0.986

BDT-S2: *a* = 0.59; *b* = 1.70; R^2 = 0.994

BDT-H1: *a* = 0.53; *b* = 1.78; R^2 = 0.993

BDT-AA: calculated data at the highest concentrations have been reported in Figure 9a for completeness, but no fitting was carried out, since the spectral shape was altered by the effect of the microcrystalline aggregates of the luminophore, causing an excessive tailing of the spectra at the longest wavelengths (Figure S8).

8. Measurement of external (η_{ext}) and internal (η_{int}) photon efficiency

All measurements were performed by using a commercially available system (Arkeo, Cicci research s.r.l., Grosseto, Italy) containing a CMOS-based spectrometer with a symmetrical Czerny-Turner optical bench connected to an integrating sphere (Figure S10).

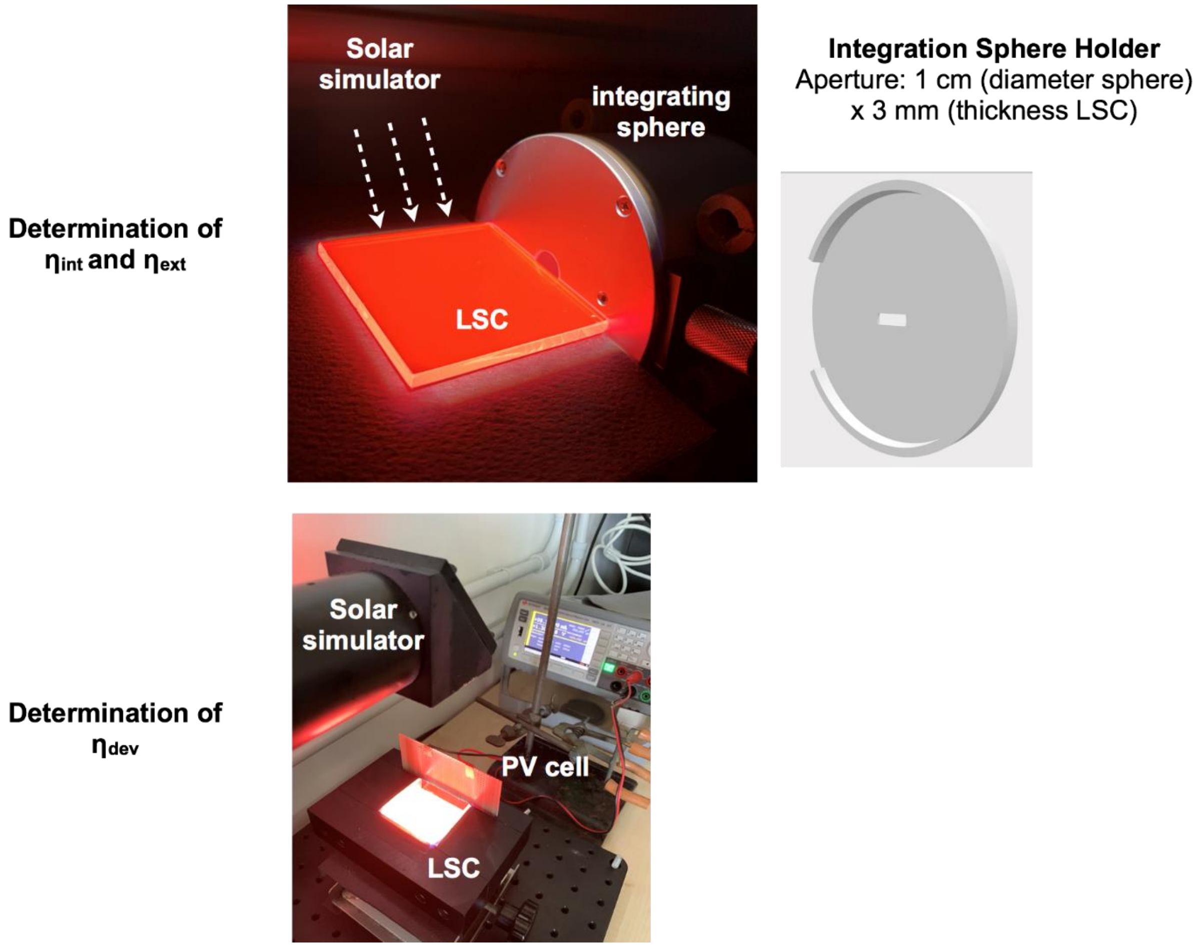

Figure S10. *Photos of the experimental setup utilized for the η_{int}, η_{ext} and η_{dev} determination*

The illumination source was an ORIEL® LCS-100 solar simulator 94011A S/N: 322 (1 Sun, AM 1.5G). An integrating sphere of 5 cm of diameter and 1 cm of aperture was placed along the edge of the glass plate. To avoid the collection of the stray light, the sphere was covered by an opaque plastic holder with a rectangular aperture of 1 cm (the width of the sphere aperture) × 3 mm (i.e., the thickness of the LSC slab). The integrating sphere was moved along the side of the LSC until all the slab edge had been scanned. The spectrally-resolved edge output photon count was collected from the CMOS-based spectrometer and calibrated into optical power (W) and then in irradiance. Aimed at limiting reflections of unabsorbed light, an absorbing matte black background was placed in contact with the LSC rear side. The illumination source was kept close and perpendicular to the center of the LSC front surface to minimize the divergence of the excitation beam and to avoid the direct illumination of the integrating sphere. A series of 3-5 measurements were repeated to allow the integration sphere to collect the maximum single-edge output power.

The optical performances of LSCs were evaluated in terms of the external (η_{ext}) and internal (η_{int}) photon efficiency, calculated from equations S4 and S5, respectively:

$$\eta_{ext} = \frac{\text{no. of edge} - \text{emitted photons}}{\text{no. of total incident photons}} = \frac{\sum_{i=1}^{i=n}\int_{\lambda_1}^{\lambda_2} P_{out,i}(\lambda)\frac{\lambda}{hc}d\lambda}{\int_{\lambda_1}^{\lambda_2} P_{in}(\lambda)\frac{\lambda}{hc}d\lambda} \quad \text{(Eq. S4)}$$

$$\eta_{int} = \frac{\text{no. of edge} - \text{emitted photons}}{\text{no. of total absorbed photons}} = \frac{\sum_{i=1}^{i=n}\int_{\lambda_1}^{\lambda_2} P_{out,i}(\lambda)\frac{\lambda}{hc}d\lambda}{\int_{\lambda_1}^{\lambda_2} P_{abs}(\lambda)\frac{\lambda}{hc}d\lambda} = \frac{\sum_{i=1}^{i=n}\int_{\lambda_1}^{\lambda_2} P_{out,i}(\lambda)\frac{\lambda}{hc}d\lambda}{\int_{\lambda_1}^{\lambda_2} P_{in}(\lambda)(1-10^{-A(\lambda)})\frac{\lambda}{hc}d\lambda} \quad \text{(Eq. S5)}$$

Where:

a) n = 4, λ_1 = 300 nm and λ_2 = 1100 nm;

b) The number of edge-emitted photons was obtained from the sum of the output power spectra measured for each edge of the LSC;

c) The total number of photons incident on the front surface of the LSC was obtained from the input power spectrum of the light source incident on the illuminated surface area of the LSC (Figure S11);

d) The number of total absorbed photons was obtained by convoluting the absorption spectrum of the LSC and the input power spectrum of the light source incident on the illuminated surface area of the LSC. Such value was also obtained by the difference between the incident input power and the power transmitted by the LSC.

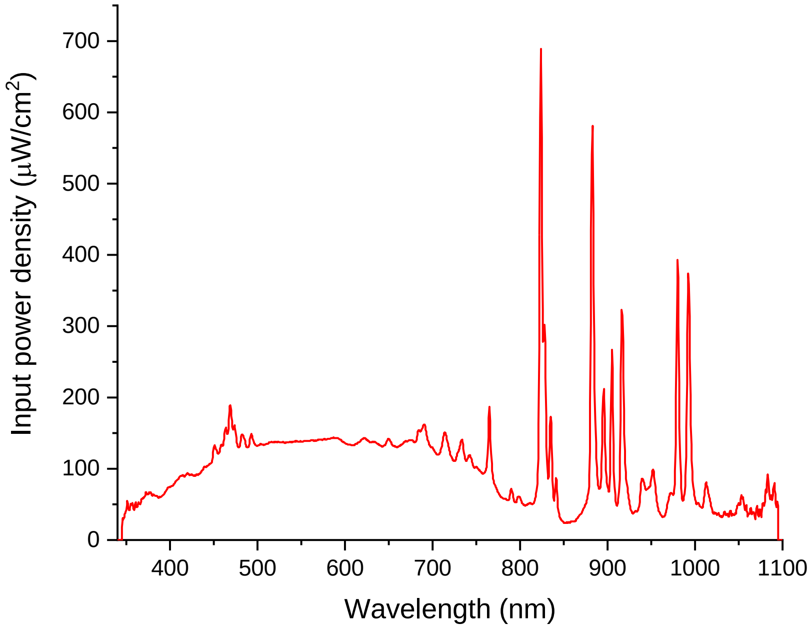

***Figure S11**. Input power density of the light source incident on the illuminated surface area of the LSC, $P_{in}(\lambda)$.*

9. Measurement of device photovoltaic efficiency (η_{dev})

The LSC photovoltaic efficiency is determined by attaching two Si-PV cells connected in series to an edge of the thin-film LSCs by using silicone grease. The performance of the assembled LSC-PV systems is assessed under standard illumination conditions by measuring the power conversion efficiency of the resulting LSC device (η_{dev}), defined as the electrical power effectively extracted from the PV cells ($P^{out}{}_{el}$) relative to the luminous power hitting the top surface of the LSC ($P^{in}{}_{opt}$):

$$\eta_{dev} = \frac{P_{el}^{out}}{P_{opt}^{in}} = \frac{I_{SC} \cdot V_{OC} \cdot ff}{P_{opt}^{in} \cdot A_{LSC}} \qquad \text{(Eq. S6)}$$

where I_{SC}, V_{OC} and ff are the short-circuit current, open-circuit voltage and fill factor of the edge-mounted PV cells, respectively, A_{LSC} is the front-illuminated area of the LSC device, and $P^{in}{}_{opt}$ is the incident solar power density expressed in mW cm^{-2}.

For the determination of η_{dev}, two Si-PV cells IXYS KXOB25-12X1F (22 x 7 mm, V_{oc} = 0.69 V, I_{sc} = 46.7 mA, ff > 70%, η = 25%) were connected in series (Figure S12 shows the *I/V* curve of the two cells assembly under direct simulated solar illumination). The current/voltage characteristics of the LSC-PV system were determined with a precision source/measure unit (Keysight Technologies B2900 Series). Silicon was used to grease the LSC edge. A black matte layer was placed beneath the LSC with an air gap of about 2.5 mm during the measurements.

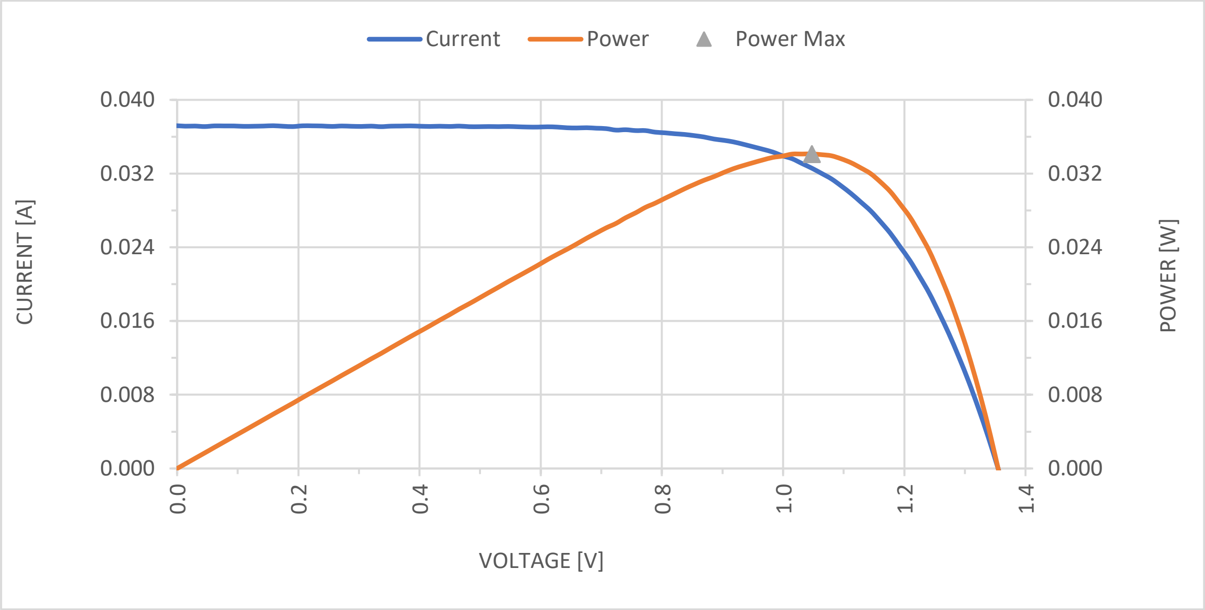

Figure S12. *I-V curve of the monocrystalline silicon solar cells (IXOLAR cells).*

10. Accelerated photodegradation test

The experiment was conducted for 650 minutes total at a constant temperature of 70 °C on a **BDT-H2**-containing PMMA film (2.2 wt.%). Wishing to comply with the ASTM G154 standard,[7] a home-made setup was used, which was composed of a LED tower (Cicci research s.r.l., Grosseto, Italy) as light source and an optical fiber connected to a spectroradiometer as the detector (CCARK.A.4.Spectroradiometer, Fiber Optic VIS/NIR spectrometer, 2048 pixels, grating VA (360-1100 nm), slit-50, OSC, DCL- UV/VIS), placed at a distance of < 1 cm from the polymeric film with a detection angle of ca. 35° (Figure S13). The sample was placed on a controllable hot stage (originally used to carry out spatially resolved photoluminescence tests with a thermal module) to adjust its temperature during the experiment.

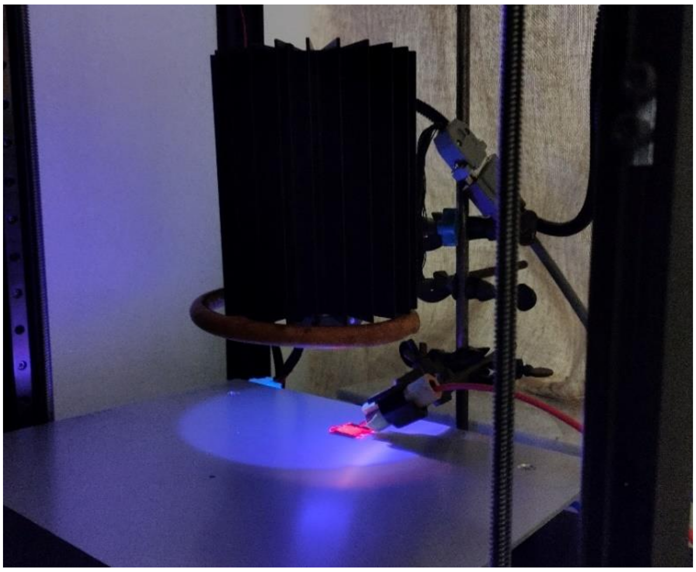

Figure S13. *Photograph of the home-built setup used for the accelerated ageing experiment.*

The experimental temperature was set directly using the hot stage control and was checked by means of a FLIR thermal camera (Figure S14). During the experiment, the entire setup was covered with a black blanket to exclude the external radiation.

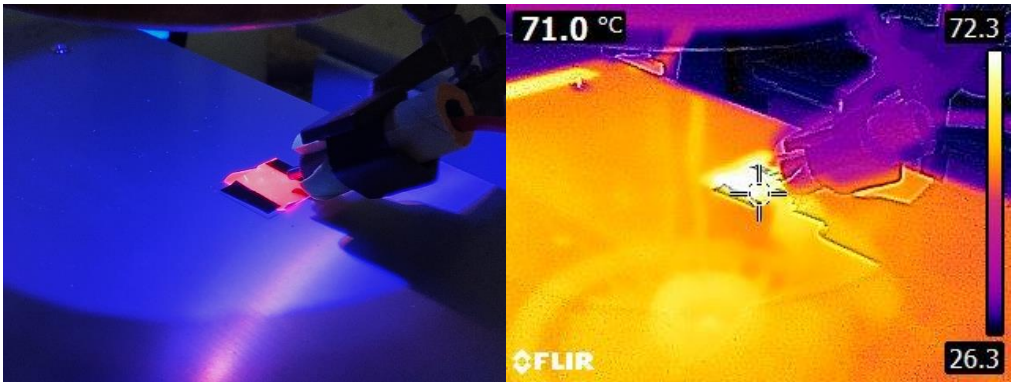

Figure S14. *Close-up of the experimental setup (left) and thermal image (right).*

As incident light sources, two of the LEDs, named "Far UV" and "UV", were selected (95% irradiation in the 361-406 nm range). Their emission was calibrated with an integrating sphere, which was placed at the same

distance from the source as that of the sample. After adjusting the LED intensity, we measured an irradiance of 38.43 W/m^2 in the selected area, to be compared with that of the AM 1.5G solar irradiation (33.45 W/m^2). The measurement thus took place at 1.15 Sun irradiation conditions.

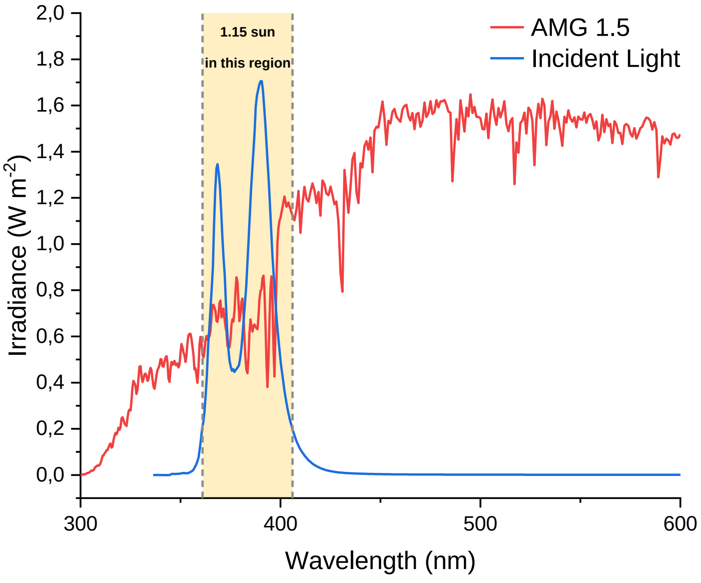

Figure S15. *Emission spectrum of the light source used for the photostability experiment, compared to the AM 1.5G spectrum. The area integrated to determine the spectral flux is highlighted in yellow.*

The emission spectra of the sample at the different experimental times and the progression of emission peak area with time are reported in Figure S16.

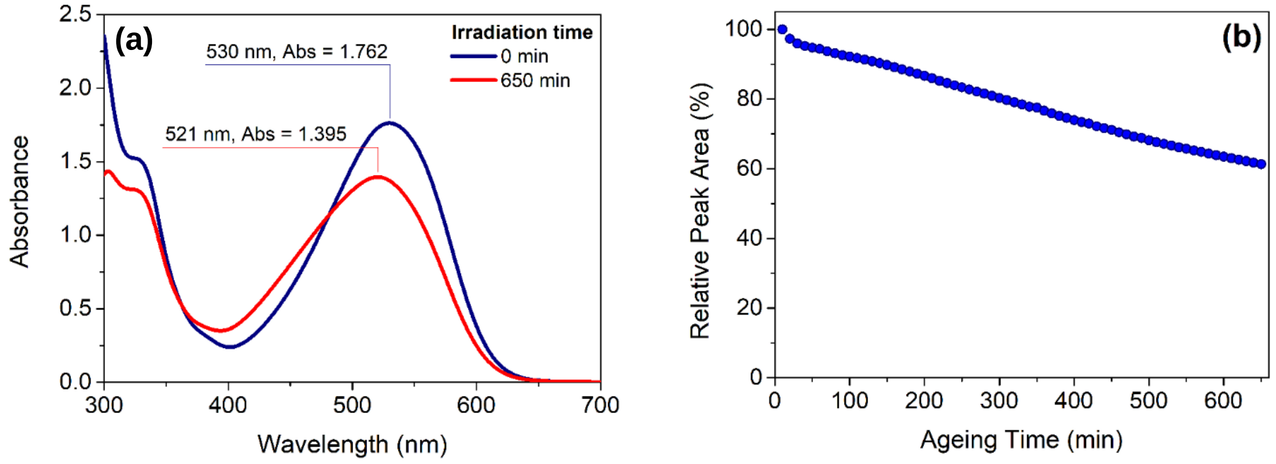

Figure S16. *(a) Absorption spectra of a **BDT-H2**-containing PMMA film (2.2 wt.%) at the beginning (blue) and at the end (red) of the accelerated ageing experiment; (b) Relative emission peak area as a function of irradiation time. Note that an initial delay time of 10 mins was considered to allow the film to reach the set temperature.*

To evaluate the real time aging period corresponding to the accelerated test, the method reported in the ASTM F1980 standard[8] was employed, which was based on the estimation of an accelerated ageing factor (*AAF*) by application of the following equation (Eq. S7).

$$AAF = Q_{10}{}^{[(T_{AA}-T_{RT})/10]} \qquad \text{(Eq. S7)}$$

Where Q_{10} is an acceleration factor indicating how many times the photodegradation rate increases for each 10 °C temperature increase, T_{AA} is the accelerated aging temperature (70°C) and T_{RT} is room temperature (22°C).

Here, we assumed a conservative (and widely accepted) value of 2 for Q_{10}, which equals to consider that the photodegradation rate doubles for each 10 °C temperature increase. By applying equation S7, a value of ca. 27.86 can be calculated for *AAF*. Then, the real time ageing period can be obtained from the product of the actual experiment duration and the *AAF*, according to Eq. S8.

$$Real\ Time\ (RT)\ ageing = Accelerated\ Ageing\ Time\ (AAT) \times AAF$$

$$= 650' \times 27.86 = 18109' = 301h\,49' \qquad \text{(Eq. S8)}$$

11. References

[1] M. Urbani, M. Medel, S. A. Kumar, M. Ince, A. N. Bhaskarwar, D. González-Rodríguez, M. Grätzel, M. K. Nazeeruddin, T. Torres, *Chem. – A Eur. J.* **2015**, *21*, 16252.

[2] T. M. Pappenfus, D. T. Seidenkranz, M. D. Lovander, T. L. Beck, B. J. Karels, K. Ogawa, D. E. Janzen, *J. Org. Chem.* **2014**, *79*, 9408.

[3] X. Cheng, M. Liang, S. Sun, Y. Shi, Z. Ma, Z. Sun, S. Xue, *Tetrahedron* **2012**, *68*, 5375.

[4] S. Zhen, S. Wang, S. Li, W. Luo, M. Gao, L. G. Ng, C. C. Goh, A. Qin, Z. Zhao, B. Liu, B. Z. Tang, *Adv. Funct. Mater.* **2018**, *28*, 1706945.

[5] M. G. Debije, R. C. Evans, G. Griffini, *Energy Environ. Sci.* **2021**, *14*, 293.

[6] C. Ceriani, F. Corsini, G. Mattioli, S. Mattiello, D. Testa, R. Po, C. Botta, G. Griffini, L. Beverina, *J. Mater. Chem. C* **2021**, *9*, 14815.

[7] ASTM G 154 (**2006**) - Standard Practice for Operating Fluorescent Light Apparatus for UV Exposure of Nonmetallic Materials, ASTM International, West Conshohocken, PA 9428-2959, United States, www.astm.org.

[8] ASTM F 1980 (**2002**) - Standard Guide for Accelerated Aging of Sterile Medical Device Packages, ASTM International, West Conshohocken, PA 9428-2959, United States, www.astm.org.
